# SEEG initiative estimates of Brazilian greenhouse gas emissions from 1970 to 2015

**DOI:** 10.1038/sdata.2018.45

**Published:** 2018-05-29

**Authors:** Tasso Rezende de Azevedo, Ciniro Costa Junior, Amintas Brandão Junior, Marcelo dos Santos Cremer, Marina Piatto, David Shiling Tsai, Paulo Barreto, Heron Martins, Márcio Sales, Tharic Galuchi, Alessandro Rodrigues, Renato Morgado, André Luis Ferreira, Felipe Barcellos e Silva, Gabriel de Freitas Viscondi, Karoline Costal dos Santos, Kamyla Borges da Cunha, Andrea Manetti, Iris Moura Esteves Coluna, Igor Reis de Albuquerque, Shigueo Watanabe Junior, Clauber Leite, Roberto Kishinami

**Affiliations:** 1 Observatório do Clima - Sistema de Estimativa de Emissões de Gases de Efeito Estufa (OC-SEEG), Rua Irmão Lucas, 101 ap3/4, São Paulo, SP 05418-060, Brazil; 2 Instituto de Manejo e Certificação Florestal e Agrícola (IMAFLORA), Estrada Chico Mendes, 185, Piracicaba, SP 13426-420, Brazil; 3 Instituto do Homem e Meio Ambiente da Amazônia (IMAZON), Ed. Zion Business - Tv. Dom Romualdo de Seixas, 1698, Belém, PA 66055-200, Brazil; 4 Instituto de Energia e Meio Ambiente (IEMA), Rua Ferreira de Araújo, 202, 10º andar, Conjunto 101, São Paulo, SP 05428-000, Brazil; 5 International Council for Local Environmental Initiatives (ICLEI), Rua Marquês de Itú, 70, 140 andar, São Paulo, SP 01223-903, Brazil; 6 CO2 Consulting, Rua Havaí, 533, 4B, São Paulo, SP 01259-000, Brazil; 7 IDEC, Rua Desembargador Guimarães, 21, São Paulo, SP 05002-000, Brazil; 8 Instituto Clima e Sociedade (ICS), Rua General Dionísio, 14, Rio de Janeiro, RJ 22271-050, Brazil

**Keywords:** Developing world, Agriculture, Forestry, Energy and society, Climate change

## Abstract

This work presents the SEEG platform, a 46-year long dataset of greenhouse gas emissions (GHG) in Brazil (1970–2015) providing more than 2 million data records for the Agriculture, Energy, Industry, Waste and Land Use Change Sectors at national and subnational levels. The SEEG dataset was developed by the Climate Observatory, a Brazilian civil society initiative, based on the IPCC guidelines and Brazilian National Inventories embedded with country specific emission factors and processes, raw data from multiple official and non-official sources, and organized together with social and economic indicators. Once completed, the SEEG dataset was converted into a spreadsheet format and shared via web-platform that, by means of simple queries, allows users to search data by emission sources and country and state activities. Because of its effectiveness in producing and making available data on a consistent and accessible basis, SEEG may significantly increase the capacity of civil society, scientists and stakeholders to understand and anticipate trends related to GHG emissions as well as its implications to public policies in Brazil.

## Background & Summary

The Paris Agreement of the United Nations Framework Convention on Climate Change (UNFCCC)^1^ from 2015 aims to hold the rise in global average temperatures by 2100 to “*well below 2 °C above pre-industrial levels and to pursue efforts to limit the temperature increase to 1.5 °C above pre-industrial levels*”. To that end, many countries—close to 200—voluntarily pledged to reduce their greenhouse gas (GHG) emissions for the agreement in their statements of Intended Nationally Determined Contributions to the UNFCCC^1^.

Current pledges reflect countries’ interests in mitigating climate change; however, priorities and capacities are limited to available data and technical options. Better understanding of the gaps will show where further investment and accelerated action are really needed^2^. Therefore, long term datasets can provide meaningful data for evaluating the performance and trends of economic sectors of a given country and increase capacity to evaluate risk management strategies^3^.

Brazil is one of the top 10 world’s biggest GHG emitters^4^ and its NDC pledges to reduce 43% of its 2005 emission level by 2030 (refs 5, 6). However, long term and up to date records are lacking for this country. Brazil has three National GHG inventories and four “update reports”^7^, but these were developed with no periodicity or international peer-review. The first national inventory was released in 2005 (ref. 8) covering the years of 1990 and 1994, the second came out in 2010 (ref. 9) covering the period of 1990-2005 and recently (2016) the third national GHG inventory was developed covering the period of 1990-2010 (ref. 10). These time gaps in generating inventories and a coverage delay of almost 5 years can miss capturing and understanding GHG emissions trends in this country and, ultimately, the design of effective policies for decarbonizing its economy and accomplishing with national and international pledges.

The System for Estimating Greenhouse Gas Emissions (SEEG)^11^ is an initiative to produce annual estimates of GHG emission in Brazil that can help in filling these gaps. The SEEG has covered 46 years (1970-2015) of GHG estimates, providing a unique dataset with more than 2 million up to date records at national and subnational levels for five sectors that are emission sources: Agriculture, Energy, Industrial Processes and Product Use, Land Use Change, and Waste^12^. The dataset also includes removals from land use change and international marine and aviation bunker emissions as well as not inventoried GHG emissions and removal sources, such as agricultural soil carbon stock variations^11,12^.

Since 2012 the SEEG has been fostered by the Climate Observatory^13^, an initiative comprising almost 40 non-governmental and civil society organizations in Brazil. In 2012, the OC selected four institutions with strong backgrounds in the major emitting sectors to coordinate the technical process of SEEG estimations production: Imazon (Land Use Change), Imaflora (Agriculture), IEMA (Energy and Industrial Processes and Product Use) and ICLEI (Waste). These organizations worked based on the guidelines of the Intergovernmental Panel on Climate Change^12,14^ embedding them with multiple sources: National Inventories, government reports, institutes, research centers, industry and other non-governmental organizations.

In 2013 the SEEG produced GHG estimates for Brazil for the 1990-2012 period. In 2014, the SEEG platform was launched, expanded and deepened. Data have been added covering the period from 1970 to 2013 (except for Land Use Change, which has data from 1990 to 2013) and also allocated by the 27 Brazilian States, allowing a new look at Brazilian emissions. Since 2015 the SEEG collection has been updated and revised^11^. The SEEG has inspired the creation of initiatives in other countries, such as Peru, which in early 2015 published estimates of GHG emissions between 1990 and 2013, and India, which published its first collection and estimates in June 2016 covering the period 2007 to 2012. Both are also available on a public Internet platform^15^.

Other products and collaborations have been derived from the SEEG, such as the Electric^16^ and Agricultural^17^ Monitors, the Brazilian Annual Land Use and Land Cover Mapping Project (MapBiomas)^18^ and the Climate Transparency Initiative (Climate Works Foundation) in Brazil (CTI-BRAZIL)^19^. The Electric^16^ and Agricultural^17^ Monitors have tools that estimate the timely emissions of these sectors constantly throughout the year. MapBiomas^18^ is a collaborative initiative involving more than twenty institutions to produce annual Brazilian soil cover maps, which are essential for assessing land use change, the main historical source of greenhouse gas emissions in the country. As for CTI-BRAZIL^19^, it uses the SEEG framework to adjust its tool for Project country emissions by 2030.

In addition, SEEG data support the production of analytical documents on the evolution of GHG emissions and an interactive platform portal to provide simple and clear methods for accessing data generated in the system^11^. Therefore, among the existing GHG emission data for Brazil known to the authors, the dataset presented here is probably the most comprehensive one in terms of transparency, number of records and time and geographic coverage.

## Methods

### SEEG system - general overview

SEEG estimations follow the latest Brazilian Inventory of Anthropogenic Emissions and Removals of Greenhouse Gases, published by Ministry of Science, Technology and Innovation (BIs)^10^. These BIs are based on the IPCC Guidelines^12,14^, but already embedded with several country specific data, which has enabled most estimations to have a Tier 2 level of precision^10,12^.

The higher the Tier used, the higher the precision in estimates^12^. In this way, it is important to notice that without the BIs and their reference reports it would not be possible to develop this initiative using Tier 2 level EFs, since the vast majority of specific emission factors were calculated in drawing up the inventory process by teams of dozens of institutions, and involving hundreds of researchers and experts^10^.

The SEEG estimations cover the GHG emissions from 1970 to 2015 for the Agriculture, Energy, Industrial Process and Waste sectors and from 1990 to 2015 for the Land Use Change sector in Brazil^11^. These emissions are further disaggregated in up to 5 new layers of sub-sectors according to each sector’s specificities, totaling 6,532 and 67,631 arrangements at national and sub-national levels, respectively.

Therefore, SEEG estimations include emission sources of all sectors and their respective gases foreseen in the IPCC Guidelines^12^ and in the BIs^10^ (Table 1), as follows:

- Agriculture. This sector covers estimations of all anthropogenic non-CO2 emissions (CH4 and N2O) from agricultural systems (livestock and cropping) soils, except for fuel combustion and sewage emissions, as follows: CH4 emissions from: i) enteric fermentation of ruminant animals, ii) animal manure management systems and, iii) burning of crop residues; N2O emissions from: i) synthetic and organic nitrogen fertilizer applied to soils, ii) cultivation of organic soils, iii) animal manure deposited directly on pastures, iv) animal manure management systems, v) decomposition of organic residues and vi) burning of crop residues.
- Land Use Change. In this sector the estimations are associated with land cover and user change mapped using satellite data. The changes in land cover represent the main levels of emissions and removals of CO2, and the changes in land use represent the level of intensity of these emissions and removals. This sector also estimates emissions from burned forest residues (CH4 and N20) and liming.
- Energy. GHG emissions occur through two different processes: (i) fuel combustion and (ii) fugitive emissions. In fuel combustion, fuel chemical energy content is converted into heat. End-use equipment direct consumption (ovens, heaters, dryers, etc.) and conversion into mechanical or electrical energy (thermal electricity generation and mobile sources) are two possible ways for this heat. During combustion, carbon (C) stored in the fuel is oxidized and released as carbon dioxide (CO_2_). There are also relatively minor emissions of other gases resulting (i) from incomplete fuel combustion - methane (CH_4_), carbon monoxide (CO) and non-methane volatile organic compounds (NMVOCs) -, and (ii) from nitrogen (N_2_) oxidation, mainly originating from air consumption in combustion, depending on process temperature - nitrogen oxides (NO_x_) and nitrous oxide (N_2_O). Fugitive emissions are intentional and unintentional releases that occur during processes of coal, oil and natural gas production. These emissions are related to fuel extraction, storage, processing and product transport activities. Inventoried gases for these activities are CO_2_, CH_4_ and N_2_O.
- Industrial Process and Product Use. GHG Emissions that occurred in chemical or physical material transformation in industrial activities. Inventoried gases are carbon dioxide (CO_2_), methane (CH_4_), nitrous oxide (N_2_O), carbon monoxide (CO), non-methane volatile organic compounds (NMVOC), nitrogen oxides (NO_x_), perfluorocarbons (CF_4_ and C_2_F_6_), hydrofluorocarbons (HFC-23, HFC-32, HFC-125, HFC-134a, HFC-143a, HFC-152a and sulphur hexafluoride (SF_6_). Industrial fuel combustion and waste disposal emissions are accounted for in the Energy and Waste sectors, respectively. The emission estimation groups are listed as follows: (i) Metal production: pig iron and steel, ferroalloys, aluminum, magnesium and other non-ferrous metal production; (ii) Mineral products: lime, cement and glass production and soda ash consumption; (iii) Chemical industry: adipic acid, phosphoric acid, nitric acid, acrylonitrile, ammonia, caprolactam, calcium carbide, vinyl chloride, ethylene, methanol, carbon black, ethylene oxide, calcined petroleum coke and other petrochemical production; (iv) Hydrofluorocarbons (HFCs) emissions; (v) Sulphur hexafluoride (SF_6_) use in electrical equipment; (vi) Non-energy products from fuel and solvent use.
- Waste. This sector covers estimations of GHG emissions from solid waste and wastewater treatment and discharge, except emissions related to the waste generated from agricultural and livestock activities, which are accounted for in the agriculture sector. The GHG emitted and their corresponding activities are: (i) CH4 emissions from solid waste disposed in landfills or dump sites and industrial and domestic wastewater treatment and discharge; (ii) N2O emissions from the incineration of clinical and hazardous waste and domestic wastewater treatment and discharge; and (iii) CO2 emissions from the incineration of clinical and hazardous waste.

All estimates are also expressed in terms of CO2 equivalents (CO2e) using GWP (Global Warming Potential) and GTP (Global Temperature Change Potential) conversion values of the second, fourth and fifth IPCC assessment reports^20–22^ (Table 2). The estimates include the emissions and removals of GEE which are presented separeted as well as Net Emissions which combine both.

### Calculation of the GHG emissions: step by step

**Figure 1:**
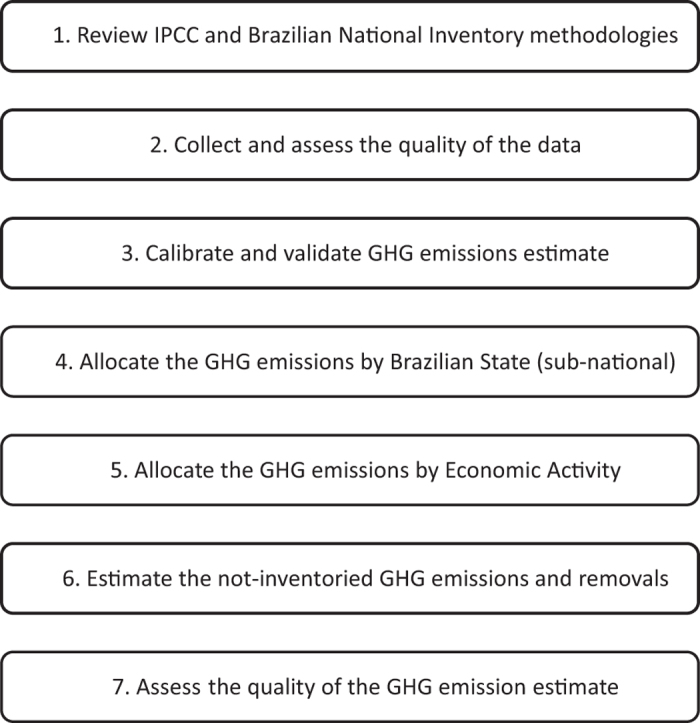
Major SEEG methodological steps.

In order to estimate GHG emissions in Brazil, SEEG assembled a calculation routine to reproduce the work of the 3 BIs, based on assessment of the original methodologies, activity data and emission factors sources^8–10^. Secondly, SEEG evaluated and validated its calculation routine and assessed the quality of results generated among relevant stakeholders and several public and private institutions. Finally, SEEG used this compiled methodology to estimate emissions beyond the period comprised in the BIs (1970-1989 and 2011-2015) and coupled them with other socio-economic indicators. These steps are showed in Figure 1 and described in details below.

#### Review IPCC and national inventory methodologies

Estimations of GHG emissions basically rely on the multiplication of an activity data and a respective emission factor^12^. Activity data is defined as data on the magnitude of human activity resulting in emissions or removals taking place during a given period of time, whereas the emission factor is the average emission rate of a given GHG for a given source of activity data^12^.

Therefore, the very first step of SEEG estimations was a literature review of the BIs^8–10^ and the IPCC Guidelines^12^. This review allowed SEEG to understand GHG emission calculations as well as to identify, verify and check activity data and evolution of emission factors, and formulas applied in published BIs.

#### Replicate and expand estimations

By assessing respective BI data sources, SEEG compiled all activity data and emission factors necessary to replicate BIs for 5 emitting sectors: Land Use Change, Agriculture, Energy, Industrial Process and Waste. To ensure estimation reproducibility, when cited activity data were not available, incomplete or private, various strategies were used to obtain the estimations, such as the search for benchmarks, trend lines and correlations with other activities (see supplementary material).

Following same data sources, SEEG also collected and updated components for emission factors to estimate GHG emissions for the period of 1970-1989 and after 2010 (not comprised in the BIs^8–10^). Eventual data gaps were also filled using mathematical approaches as cited above, which are described in details in the supplementary material.

#### Allocate GHG emissions in subnational regions

Since its second version (released in 2014) SEEG has allocated national estimates at sub-national level (Table 3), totaling 27 locations (including the Federal District) (Table 4). This allocation was made by using emissions-generating activities, such as deforestation rate, fuel sales for transportation and industrial production, or even recollecting available activity data and emission factors for specific sub-national locations (see details below).

#### Estimate emissions for production and economic activities

In order to visualize a more accurate picture of the sectors of the Brazilian economy and improve data correlation capacity, GHG estimations of all sectors are also associated to their respective economic activity and/or product output (Tables 5 and 6).

#### Review and assess the quality of the data

Finally, the quality of the methodology and dataset generated by SEEG are evaluated according to its capacity in 1) reproduce the BIs; 2) allocate total emissions at sub-national level and; 3) estimate GHG emissions beyond the BI coverage (Table 7).

These evaluations basically follow criteria regarding the nature and acquisition of activity data and emission factors (Table 5). Such analyses help SEEG to improve the transparency of its methodology as well as to inform the public, decision makers and institutions on areas of prioritization where data acquisition and/or updates (i.e. surveys, methods reviewing and expert consultations) could enhance estimation accuracy.

#### Estimate GHG emissions not yet inventoried

- Soil Carbon Stock Variation in Agricultural Areas: the variation in soil carbon stocks refers to CO2 emissions and removals related to the soil organic matter^12^. This variation is not reported in the national inventories due to the difficulty in obtaining the activities data and CO2 emission and removal factors for this calculation as well as factors related to carbon permanence^12^. However, due to the importance of soil carbon in the GHG emissions balance of the Agricultural Sector^23^ as well as to the fact the Brazilian NDC's success also relies on soil carbon sequestration (CO_2_ removal) in agricultural soils^5, 6, 24^, the fourth SEEG collection made an exercise of calculating this variation for soils used by Brazilian agriculture under: commercial forest plantations (for pulp and paper primarily), crops (conventional tillage and no-tillage), integrated production systems (combination of crop, livestock and forest) and pastures (degraded and improved) (see details below). However, it is important to note that the estimation of carbon stock variation of agricultural soils made by SEEG is merely an exercise and relies mostly on expert consultation and a few factors (CO2 emission and removal) available in literature for Brazilian conditions (see details below). Soil carbon stock variation is highly uncertain by nature. Soil type, agricultural management and land use history are pointed out as the main drivers of soil carbon stock variation overtime^23, 25^. And no scientific consensus has been reached on how much soil carbon actually varies under the combination of different natural and management factors^26, 27^ as well as the agricultural area under different management and its soil conditions in Brazil^18, 28^. Therefore, the estimates presented are likely to have high uncertainties and should be used with care. On the other hand, these estimates provide a framework able to calculate carbon stock variation of agriculture soils as well as to be improved as science advances on that issue.

### Calculation of the GHG emissions: Agriculture sector

#### Methane emissions from enteric fermentation

SEEG estimates CH_4_ emissions from enteric fermentation of dairy cattle, beef cattle, buffalo, sheep, goats, horses, mules, asses and swine, according to the following equation^12^:

$$E_{FE}=\sum_{A}PAxFE_{FE}x10^{-9}$$

Where: E_FE_=Emission of CH_4_ from enteric fermentation by animal type A (Gg CH_4_); PA=Animal herd of a given type A [heads]; FE_FE_=Emission factor of CH_4_ emissions from enteric fermentation by animal type A [g/CH_4_/animal/year]; 10^−9^=conversion factor from g to Gg.

### Activity data

#### Animal population (PA)

Animal herd population (bovine, buffalo, sheep, goats, horse, mule, asses and swine) was obtained in IBGE/SIDRA^29^ database at a state level. Eventual lack of data was filled using interpolations, extrapolations and comparisons, as discussed below.

IBGE/SIDRA^29^ makes available herd population data at state level of total bovines, milk cows, swine, buffalo, horses, sheep, goats, mules and asses for each Brazilian state for 1970 and for the period of 1974 to 2015. Beef cattle data are obtained by subtracting the number of milk cows from total bovines, and further split in three categories (male, female and calf), to fit Tier 2 emission factors, using the proportions defined in MCTI^30^.

For the period of 1971-1973 data were calculated using linear interpolation of 1970 and 2015,except for assess and mules for 2013-2015, whose populations were estimate by linear projections of the last five years (starting in 2008) due to lack of these data^29^. And for dairy cows, that state level for 1970 was obtained by correlations with milk production following 3 steps: 1) SEEG calculated the ratio of number of milk cows and milk production for the 1970’s using average data for the 70’s for each Brazilian State (in order to minimize possible unusual effects of a given year); 2) the calculated ratio was multiplied by the milk production of 1970 for estimating the number of dairy cows in this year; and 3) the number of dairy cows in each Brazilian State in 1971, 1972 and 1973 was calculated using linear interpolation.

#### Emission factor (EF_FE_)

The emission factors used for calculating the emission of CH_4_ emissions from enteric fermentation for each beef cattle type at a state level for the period of 1990-2010 are available in MCTI^30^. These emissions factor have a Tier 2 level^12^, reflecting specific Brazilian conditions better than Tier 1 emission factors. For the period of 1970-1989 and 2011-2015 SEEG used the emissions factor of 1990 and 2010, respectively. For emissions of CH4 from enteric fermentation of buffalo, sheep, goats, mules, asses and swine, MCTI^30^ as well as SEEG^11^, emission factor Tier 1 level^12^ was used, applied to the period of 1970 to 2015.

#### Methane emissions from manure management

SEEG estimates CH_4_ emissions from manure management enteric of dairy cow, beef cattle, buffalo, sheep, goats, horses, mules, asses, swine and poultry, according to the following equation^12^:

$$E_{MDA}=\sum_{A}PAxFE_{MD}x10^{-9}$$

Where, E_MDA_=emission of CH4 from manure management by animal type A (Gg CH_4_); PA=animal herd of a given type A (heads); FE_MD_=emission factor of CH_4_ from manure management of a given animal type A (g/CH_4_/animal/year); 10^−9^=conversion factor from g to Gg.

#### Activity data

SEEG used the same database compiled to calculate the CH_4_ emissions from enteric fermentation^30^. In addition, due to the level of detail provided by the Brazilian Inventory, it was necessary to include the poultry flocks of layers, broilers, roosters and quail. These last herd populations were obtained from IBGE/SIDRA^29^ for the period of 1974-2015 at state level and for total poultry for 1970. In order to obtain state level data for 1970-1973, SEEG used the following steps: 1) the average of the percentage of each state’s share in the Brazilian poultry population was calculated using data for the 70’s for each Brazilian State (in order to minimize possible unusual effects of a given year); 2) this percentage was multiplied by the total number of poultry population of the year of 1970 (data available), estimating the number of the poultry population by state and; 3) the poultry population of the years of 1971, 1972 and 1973 was calculated using linear interpolation.

In addition, following particularities of the Brazilian National Inventory^10^, it was necessary to separate the swine population raised by small and medium/large holders (given that they have different emissions factors) according to a proportion described in MCTI^30, 31^.

#### Emission factor (FE_MD_)

The emission factors used for calculating the emission of CH_4_ emissions from manure management for the period of 1990-2010 are available in MCTI^30^. These emissions factor have a Tier 2 level^12^ for beef and milk cattle and swine, reflecting specific Brazilian conditions better than Tier 1 emission factors. For the period of 1970-1989 and 2011-2015 SEEG used the emissions factors of 1990 and 2010, respectively.

Emissions of CH_4_ from manure management of buffalo, sheep, goats, mules, asses and swine, MCTI^30^ as well as SEEG used emission factor Tier 1 level^12^, which were applied to the period of 1970 to 2015.

#### Nitrous oxide emissions from manure management

SEEG estimates N_2_O emissions from manure management of dairy cow, beef cattle, buffalo, sheep, goats, horses, mules, asses, swine and poultry, according to the following equation^12^:

$$E_{DP}=\sum_{A}PAxN_{ex}xF_{tA}xFE_{3}xF_{c}x10^{-6}$$

Where: E_DP_=Emission of N_2_O from manure management (not left on pasturelands) by animal type A (Gg N_2_O); PA=animal herd of a given type A (heads); N_ex_=Quantity of nitrogen excreted (Nex) by animal type A (kg N/animal/year); F_tA_=fraction of the Nex that is managed by animal type A (%); FE_3_=emission factor of N2O from the manure management (kg N-N_2_O per kg Nex under management); ${\;F}_{c}\;\;$=conversion factor from N to N_2_O (44/28); 10^−6^=conversion factor from kg to Gg.

### Activity data

#### Animal population (PA)

SEEG used the same database compiled to calculate the CH_4_ emissions from manure management^30^. However, in order to follow the Brazilian National Inventory, the beef cattle population had to be further divided into younger than 1 year old, between 1 and 2 years old and older than 2 years; swine for breeding (younger than 6 months) and market (older than 6 months) types; goat and sheep younger and older than 1 year; and poultry into layers, broilers and, roosters. Percentages applied are given in MCTI^31^. An exception is applied to obtain state level population of poultry subgroups, such as chickens layers and broilers, roosters and quail, which are not provided by IBGE/SIDRA^29^ for the period 1970 to 1973. To estimate them the following steps were developed: 1) For each State, SEEG calculated the average proportion of the population of chickens layers and broilers, roosters and quail in relation to the total number of poultry in the 70’s; 2) For each State, SEEG multiplied these proportions for the total amount of poultry each state had in 1970, thus yielding the quantities of these three animal categories for each State that year and; 3) The number of animals of these three categories in each state in 1971, 1972 and 1973 was estimated by interpolating the values of the 1970’s (obtained in step 2) and 1974 using linear interpolation.

#### Fraction of N excreted (Nex) and subjected to treatment (F_tA_)

The amount of N excreted (kg N/animal / year) and the fraction of that amount receiving treatment (not left in pasture) (F_tA_) for each type of animal and Brazilian State are available in MCTI^31^, except for beef cattle, dairy cows and swine, whose fractions are described in MCTI^30^. These fractions were used for calculating the percentage of N excreted, which is managed under different manure management systems.

#### Emission Factor (FE_3_)

The N_2_O emission factor for the animal manure management systems (FE_3_), ranging from 0.1% to 2.0% of N contained in manure under management, is available in MCTI^31^.

#### Direct nitrous oxide emissions from synthetic nitrogen fertilizers applied to soils

SEEG estimates direct N_2_O emissions from synthetic nitrogen fertilizers applied to soils according to the following equation^12^:

$$E_{FS}=N_{FERT}x\left(1-FRAC_{gasf}\right)xFE_{1}xFcx10^{-6}$$

Where:E_FS_ =direct N_2_O emissions from synthetic nitrogen fertilizer applied to soil (Gg de N_2_O); N_FERT_=quantity of N in fertilizer applied to soil (kg); FRAC_gasf_=fraction of the N-fertilizer volatilized as NH_3_ and NOx (%); FE_1_=emission factor of direct N_2_O emissions from synthetic nitrogen fertilizer applied to soil (kg N-N_2_O per kg of N applied); Fc=conversion factor from N to N_2_O (44/28); 10^−6^=conversion factor from kg to Gg.

### Activity data

#### Quantity of N in synthetic fertilizer applied to soil (N_FERT_)

The amount of N applied to the soil as synthetic fertilizer were obtained at state level at the Brazilian National Association for the Promotion of Fertilizers (ANDA) yearbooks^32–62^ for the period 1986-2015, except for northern Brazilian states (Rondônia, Acre, Amazonas, Roraima, Pará and Amapá) from 1986 to 2004 whose data were aggregated in the ANDA yearbooks. Disaggregation was carried out by dividing the total quantity of N-fertilizer of the northern states by the states (Rondônia, Acre, Amazonas, Roraima, Pará and Amapá) according to their average N-fertilizer usage for the period of 2005-2010 (Rondônia, Acre, Amazonas, Roraima, Pará and Amapá). Data for the years of 1970-1985 were estimated based on data from 1980 to 2004 for Brazil^63^, following two steps: 1) SEEG calculated the average ratio of N fertilizer consumption by state in the period between 1986 and 1989 (data for the 1980’s) and; 2) SEEG multiplied the proportion of each state by the total amount of nitrogen synthetic fertilizers consumed by Brazil^63^. Subsequently, the amount of nitrogen synthetic fertilizers of each state and year was divided into two types: urea and "other" nitrogen fertilizers (i.e.: ammonium sulfate and ammonium nitrate). This division was based on the proportions described in MCTI^31^ and is due to different rates of volatilization of NH_3_ and NOx in these two types of fertilizers.

#### Factor of N losses from volatilization (FRAC_gasf_)

For calculating the nitrogen loss from volatilization of NH3 and NOx (FracGASF), a factor of 30% per kg of urea applied to the soil and 10% per kg of other nitrogen fertilizers was used^31^.

#### Emission factor of (FE_1_)

The N_2_O emission factor used was 1% per kg of urea and other fertilizers applied to the soil after discounting the N lost through volatilization of NH_3_ and Nox^31^. It is important to mention that the N_2_O emission factor adopted by the methodology used^31^ is less than the factor of 1.25% proposed by the IPCC^12–14^. According to MCTI^31^, this fact is due to the development of research in national conditions suggesting that N_2_O emissions by the application of nitrogen fertilizers are lower than proposed by the IPCC^12–14^.

#### Nitrous oxide emissions from animal manure applied to soil as fertilizer

SEEG estimates N_2_O emissions from animal manure applied to soil as fertilizer, according to the following equation^12^:

$$E_{AA}=\sum_{A}PAxN_{ex}x\left(1-FRAC_{gasm}\right)x\left(1-FRAC_{PRP}\right)xFE_{1}xF_{c}x10^{-6}$$

Where:E_AA_=direct emissions of N_2_O from animal manure applied to soil as fertilizer (Gg); PA=animal herd of a given type A (head); N_ex_=quantity of N excreted by animal type A (kg N/animal/year); FRAC_gasm_=fraction of N in manure applied to soil lost from volatilization of NH_3_ and NOx by animal type A (%); FRAC_PRP_=fraction of N in manure excreted and left on pastures by animal type A (%); ${\mathrm{FE}}_{1\;}$=emission factor of N_2_O from manure applied to soil as fertilizer by animal type A (kg N-N_2_O per kg of N applied); Fc=conversion factor from N to N_2_O (44/28); 10^−6^=conversion factor of kg to Gg.

### Activity data

#### Animal Population (PA)

Activity data for estimating direct N_2_O emissions from animal manure applied to soil as fertilizer are the same used for calculating the N_2_O emissions from manure management^31^.

#### N excreted (N_ex_) and the fraction left on pastures (FRAC_PRP_)

The amount of N excreted (kg N/animal/year) and the amount receiving treatment (not left on pasture) (FRAC_PRP_) for each type of animal and Brazilian State are available in MCTI^31^,except for beef cattle, dairy cows and pigs, whose data are described in MCTI^30^. These fractions were used to calculate the percentage excreted N in manure handled according to this manure management systems.

#### Factor of N losses from volatilization (FRAC_gasm_)

We used a factor of loss by volatilization of NH_3_ and NOx (FracGASM) 0.02 kg NH_3_ and NOx per kg of N-manure used as fertilizer^31^.

#### Emission factor (FE_1_)

For the calculation of direct N_2_O emissions from animal manure applied to soil as fertilizer, an emission factor of 0.01 kg N_2_O per kg N used as fertilizer was used, discounting the N lost from volatilization^31^.

#### Nitrous oxide emissions from vinasse (from ethanol production) applied to soil as fertilizer

For the estimation of direct N_2_O emissions from vinasse applied to soil as fertilizer (from ethanol production from sugarcane), SEEG replicated the methodology described in MCTI^31^ according to the following equation:

$$E_{V}=\sum_{A}\left(P_{E}xP_{EV}xN_{V}\right)xFE_{v}xF_{c}x10^{-6}$$

Where: E_V_=emissions of direct N_2_O from vinasse applied to soil as fertilizer (Gg); P_E_=ethanol (from sugarcane) production; P_VE_=ratio of vinasse: ethanol production (L/L); Nv=quantity of N in vinasse (kg N/L); ${\mathrm{FE}}_{v\;}$=emission factor of N_2_O from vinasse applied to soil as fertilizer (kg N-N_2_O per kg of N applied); Fc=conversion factor of N to N_2_O (44/28); 10^−6^=conversion factor of kg to Gg.

### Activity data

#### Vinasse production

The amount of vinasse, a byproduct of the process of producing ethanol from sugarcane that is applied as organic fertilizer was estimated based on the production of ethanol (PE) at state level using data from the Sugarcane Industry Association^64^, from 1981-2015. For the years 1970-1980, data were estimated based on the National Energy Balance (BEN) yearbook^65^. However, these data refer to the total produced in Brazil. Therefore, state level data was obtained by: 1) calculating the average ratio of the state ethanol production in the period of 1981-1990 in relation to the total amount consumed in Brazil; 2) multiplying this ratio by the total ethanol produced in Brazil between 1970-1980 (ref. 65).

#### Production proportion vinasse: ethanol (P_VE_) and N quantity in vinasse (N_V_)

The value of the ratio of vinasse production per liter of ethanol produced and the amount of N contained in vinasse were 13 L and 0.357 g/L, respectively^31^.

#### Emission factor (FE_V_)

For calculating direct emissions of N_2_O SEEG used an emission factor of (FEV) of 1.94% per kg of N applied to the field via vinasse^31^. In the application of vinasse to soil there is no significant loss of N from volatilization, but leaching and runoff only^31^.

#### Nitrous oxide emissions from animal manure excreted and left on pasturelands

SEEG estimates N_2_O emissions from animal manure excreted and left on pasturelands, according to the following equation^12^:

$$E_{DP}=\sum_{A}PAxN_{ex}xFRAC_{PRP}xFE_{P}xF_{c}x10^{-6}$$

Where:E_DP_=direct N_2_O emissions from animal manure excreted and left on pasture (Gg); PA=animal population A (head); N_ex_=quantity of N excreted by animal type A (kg N/animal/year); FRAC_PRP_=fraction of the total N excreted and left on pasture by animal type A (%); ${\mathrm{FE}}_{P\;}$=N_2_O emission factor from animal manure excreted and left on pasture by animal type A (kg N-N_2_O per kg of N applied); ${\;F}_{c}\;\;$=conversion factor of N to N_2_O (44/28); 10^−6^=conversion factor of kg to Gg.

### Activity data

#### Animal population (PA)

Key data for estimating direct N_2_O emissions from animal manure deposited and left on pastures is the same used for calculating the N_2_O emissions from manure management^31^.

#### N excreted (N_ex_) and fraction deposited and left on pasture (FRAC_PRP_)

The amount of N excreted (kg N / animal / year) and the fraction of that deposited and left on pasture (FRAC_PRP_) for each type of animal and Brazilian State are available in MCTI (2015b). Except for beef cattle, dairy cows and swine which fractions are described in MCTI^30^.

#### Emission factor (FE_p_)

For the calculation of direct N_2_O emissions SEEG used an emission factor (FE_1_) of 1.5% per kg of N from animal manure deposited and left on pastures^31^.

#### Nitrous oxide emissions from crop residues decomposition

SEEG estimates N_2_O emissions from crop residues, according to the following equation^12^:

$$E_{RC}=\sum_{A}P_{c}x{\mathrm{FRAC}}_{\mathrm{DM}}x\frac{\mathrm{RESDM}}{\mathrm{CROPDM}}x{\mathrm{FRAC}}_{\mathrm{NCR}}xF_{c\;}xFE_{1}\;x\;10^{-6}$$

Where: E_RC_=N_2_O emissions from crop residues; P_c_=production of the agriculture crop A (kg); FRAC_DM_=dry matter fraction of the crop product A; (RESDM)/(CROPDM)=ratio of crop residue and product for crop A; FRAC_NCR_=N content of the residue of crop A; FE_1_=emission factor of N_2_O (kg N- N_2_O per kg N in crop residue); $F_{c\;}$=converstion factor of N to N_2_O (44/28); 10^−6^=conversion factor of kg to Gg.

### Activity data

#### Crop production (P_C_)

SEEG estimates direct N_2_O emissions from nitrogen that returns and decomposes in the soil from crop residues of: soybeans, sugar cane, beans, rice, corn, cassava and "other crops". The latter comprises the crops: pineapple, cotton, peanuts, oat, sweet potatoes, potatoes, rye, barley, peas, beans, sunflower, linseed, castor beans, watermelon, melon, sorghum, tomatoes, wheat and triticale. The data source of these data was the IBGE/SIDRA^29^. For the years 1975 to 1989, information was obtained by request through the answering service of IBGE (e-mail: ibge@ibge.gov.br), since data for this period were not available on IBGE's website for consultation.

The values for the period of 1971-1974 were obtained by linear interpolation of 1970 and 1975 data. For the year 1970, data for sorghum, sunflower, triticale and linseed were not available on the IBGE/SIDRA^29^ and were obtained by linear projection of 1975-1979 values. However, it should be noted that the values were not designed when there was no production in 1975, for example, production was assumed to be equal to zero from 1970 to 1974 when production in 1975 was zero. The melon and watermelon data to 2000 and for pineapple (all available periods) are presented in "thousand units." To convert them to tons an average weight of 1.39 kg was adopted for melon, watermelon and 6.25 to 1.6 kg for pineapple^31^.

#### Crop (FRAC_DM_), N content (FRAC_NCR_) and ratio residue:product (dry matter) ($\frac{RESDM}{CROPDM})$

The necessary parameters for calculating N_2_O emissions from crop residues left on soil (FRAC_DM_, FRAC_NCR_ and $\frac{RESDM}{CROPDM})\;$ were obtained in MCTI^31^.

#### Emission factor

For calculation of the direct N_2_O emissions for agricultural crop residues, an emission factor of N_2_O emission (FE_1_) of 0.01 kg of N per kg of N in crop residues that return to soil was used^31^.

#### Nitrous oxide emissions from cultivation of organic soils

SEEG estimates N2O emissions from organic soils according to the following equation^12^:

$$E_{SO}=A_{SC}xFE_{2}xF_{C}x10^{-6}$$

Where: E_SO_=N_2_O emissions from cultivated organic soils (Gg); A_SC_=area of cultivated organic soils (ha); FE_2_=emission factor of N_2_O (kg of N-N_2_O per ha under cultivation); F_C_=conversion factor of N to N_2_O (44/28); 10^−6^=conversion factor of kg to Gg.

### Activity data

#### Area of organic soils under cultivation (A_SC_)

Using land use soil maps, MCTI^10^ estimated that Brazil has 1.59 million hectares of organic soils, located in 12 Brazilian states. The state division of these areas is shown in MCTI^31^. However, a portion of less than 8% of these areas covers the borders of three states (Paraná, Santa Catarina and Mato Grosso do Sul) that were not separated^31^. On the other hand, MCTI^31^ estimated that in 1990, 47.5% of the total organic soils was under agriculture cultivation, increasing to 51.5% in 2010 (ref. 31), but not at a state level. To deal with this limitation, SEEG took the following steps: 1) data over state borders was divided by the number of states involved equally; 2) it was assumed that areas of organosols for each State, provided by MCTI^31^, had the same proportions of agricultural use for Brazil as a whole (i.e., 47.5% and 51.5% for 1990 and 2010, respectively); 3) the ratio of use of organosols was calculated based on data from 1990 and 2010 (47.5% and 51.5% of use, respectively) ‒0.2% per year during that period and; 4) these proportions and ratio was applied to the area of organosols of each state, positively from 2007 to 2014 and negatively from 1970 to 1989.

#### Emission factor

For calculating direct emissions from organic soils, an emission factor (FE_2_) of 12 kg N_2_O per hectare of cultivated organosol was used^31^.

#### Indirect nitrous oxide emissions from atmospheric deposition of the nitrogen volatilized from synthetic fertilizer applied to soils

SEEG estimates indirect N_2_O emissions from atmospheric deposition of N volatilized from the application of synthetic fertilizer applied to soil according to the following equation^12^:

$$E_{DN}=N_{FERT}xFRAC_{gasf}xFE_{4}xF_{C}x10^{-6}$$

Where: E_FS_ =Indirect emissions of N_2_O from deposition of N volatilized from synthetic fertilizer applied to soil (Gg of N_2_O); N_FERT_=quantity of N in synthetic fertilizer applied to soil (kg); FRAC_gasf_=fraction of the N applied to soil volatilized as NH_3_ and NOx (%); FE_4_=emission factor of N_2_O (kg N-N_2_O per kg of N deposited); Fc=conversion factor of N to N_2_O (44/28); 10^−6^=conversion factor of kg to Gg.

### Activity data

#### Quantity of N from synthetic fertilizer applied to soil (N_FERT_)

Key data for estimating indirect emissions from synthetic fertilizers applied to soil is the amount of N applied as fertilizer^31^.

#### Factor of N losses from volatilization (FRAC_gasf_)

For the calculation of the nitrogen loss from volatilization of NH_3_ and NOx (FracGASF), a factor of 30% per kg of urea applied to the soil and 10% per kg of other nitrogen fertilizers was used^31^.

#### Emission factor (FE_1_)

For the calculation of indirect emissions of N_2_O, SEEG applied an emission factor (FE1) of 0.01 kg N-N_2_O per kg of volatilized N deposited on soil^31^.

#### Indirect nitrous oxide emissions from atmospheric deposition of the nitrogen volatilized from animal manure applied to soils

SEEG estimates indirect N_2_O emissions from atmospheric deposition of the nitrogen volatilized from animal manure applied to soils according to the following equation^12^:

$$E_{DA}=\sum_{A}PAxN_{ex}x\left(1-FRAC_{PRP}\right)xFRAC_{gasm}xFE_{4}xF_{C}x10^{-6}$$

Where: E_DA_=indirect N_2_O emissions from deposition of N volatilized from animal manure application to soil as fertilizer (Gg); PA=animal population A (head); Nex=quantity of N excreted by animal type A (kg N/animal/year); FRAC_PRP_=fraction of the total N excreted left on pastures by animal type A (%); FRAC_gasm_=fraction of the N applied to soil that volatilizes as NH_3_ and NOx (%); ${\mathrm{FE}}_{4\;}$=emission factor of N_2_O from soil deposition of N volatilized (kg N-N_2_O per kg of N deposited); ${\;F}_{c}\;\;$=conversion factor of N to N_2_O (44/28); 10^−6^=conversion factor of kg to Gg.

### Activity Data

#### Animal Population (PA)

SEEG used the same database compiled to calculate the N_2_O emissions from animal manure management^31^.

N excreted (*N*_*ex*_) and fraction deposited on pastures (*FRAC*_*PRP*_) by categories of animals

The amount of N excreted (kg N/animal/year) and the fraction of that amount receiving treatment (not left on pasture) (F_tA_) for each type of animal and Brazilian State^31^. Except for beef cattle, dairy cows and swine, whose fractions are described in MCTI^30^. These fractions were used for calculating the percentage of N excreted which is managed under different manure management systems.

#### Factor of N loss from volatilization (FRAC_GASM_)

SEEG used a N loss factor due to volatilization of NH_3_ and NOx (FracGASM) of 0.02 kg of NH3 and NOx per kg of N contained in the animal manure applied to soil as fertilizer^31^.

#### Emission factor (FE_1_)

For the calculation of indirect emissions of N_2_O, SEEG applied an emission factor (FE_1_) of 0.01 kg N-N_2_O per kg of volatilized N deposited on soil^31^.

#### Indirect nitrous oxide emissions from nitrogen leaching and runoff from synthetic fertilizer applied to soil

SEEG estimates indirect N_2_O emissions from N leaching and runoff from the application of synthetic fertilizer applied to soil according to the following equation^12^:

$$E_{LS}=N_{FERT}xFRAC_{leach}xFE_{5}xF_{C}x10^{-6}$$

Where: E_LS_ =Indirect emissions of N_2_O from N leaching and runoff from synthetic fertilizer applied to soil (Gg of N_2_O);N_FERT_=quantity of N in synthetic fertilizer applied to soil (kg); FRAC_leach_=fraction of the N that is leached and runoff from synthetic fertilizer applied to soil (%); FE_5_=emission factor of N_2_O (kg N-N_2_O per kg of N leached and runoff); Fc=conversion factor of N to N_2_O (44/28); 10^−6^=conversion factor of kg to Gg.

### Activity data

#### Quantity of N from synthetic fertilizer applied to soil (N_FERT_)

A key data item for estimating indirect emissions from synthetic fertilizers applied to soil is the amount of N applied as fertilizer. Therefore, we used the same database compiled to calculate direct N_2_O emissions for synthetic fertilizers^31^.

#### Factor of N losses from leaching and runoff (FRAC_leach_)

For the calculation of the nitrogen loss from leaching and runoff (Frac_leach_), a factor of 30% per kg of urea applied to the soil and 10% per kg of other nitrogen fertilizers were used^31^.

#### Emission factor (FE_5_)

For the calculation of indirect emissions of N_2_O, SEEG applied an emission factor (FE_5_) of 0.025 kg N-N_2_O per kg of N leached and runoff after a synthetic fertilizer application to soil^31^.

#### Indirect nitrous oxide emissions from nitrogen leaching and runoff from animal manure applied to soil as fertilizer

SEEG estimates indirect N_2_O emissions from leaching and runoff of the nitrogen from animal manure applied to soils according to the following equation^12^:

$$E_{LA}=\sum_{A}N_{ex}x\left(1-FRAC_{PRP}\right)xFRAC_{leach}xFE_{5}xF_{C}x10^{-6}$$

Where: E_LA_=indirect N_2_O emissions from leaching and runoff of N in animal manure applied to soil as fertilizer (Gg); PA=animal population A (heads); Nex=quantity of N excreted by animal type A (kg N/animal/year); FRAC_PRP_=fraction of the total N excreted left on pastures by animal type A (%); FRAC_leach_=fraction of the N applied to soil leached and runoff (%); ${\mathrm{FE}}_{5\;}$=emission factor of N_2_O from N applied to soil leached and runoff (kg N-N_2_O per kg of N leached and runoff); ${\;F}_{c}\;\;$=conversion factor of N to N_2_O (44/28); 10^−6^=conversion factor of kg to Gg.

### Activity Data

#### Animal Population (PA)

SEEG used the same database compiled to calculate the N_2_O emissions from animal manure management^31^.

N excreted (N_ex_) and fraction deposited on pastures (*FRAC*_*PRP*_) by categories of animals

The amount of N excreted (kg N/animal/year) and the fraction of that amount receiving treatment (not left on pasture) (F_tA_) for each type of animal and Brazilian State^31^, except for beef cattle, dairy cows and swine whose fractions are described in MCTI^30^. These fractions were used for calculating the percentage of N excreted, which is managed under different manure management systems.

#### Factor of N loss from leaching and runoff (FRAC_leach_)

SEEG used a N loss factor due to leaching and runoff of 30% of the N in manure applied as fertilizer^31^.

#### Emission factor (FE_5_)

For the calculation of indirect emissions of N_2_O, SEEG applied an emission factor (FE_5_) of 0.025 kg N-N_2_O per kg of N leached and runoff after manure application to soil^31^.

#### Indirect nitrous oxide emissions from nitrogen leaching and runoff from vinasse applied to soil as fertilizer

SEEG estimates indirect N_2_O emissions from leaching and runoff of the nitrogen in vinasse applied to soils according to the following equation^12^:

$$E_{VL}=\sum_{A}\left(P_{E}xP_{EV}xN_{V}\right)xFRAC_{leach}xFE_{5}xF_{c}x10^{-6}$$

Where:E_VL_=indirect N_2_O emissions from leaching and runoff of N in vinasse applied to soil as fertilizer (Gg); P_E_=ethanol production (L); P_VE_=vinasse:ethanol production ratio (L/L); Nv=quantity of N in vinasse (kg N/L); FRAC_leach_=fraction of the N applied to soil leached and runoff (%); ${\mathrm{FE}}_{5\;}$=emission factor of N_2_O from N applied to soil leached and runoff (kg N-N_2_O per kg of N leached and runoff); ${\;F}_{c}\;\;$=conversion factor of N to N_2_O (44/28); 10^−6^=conversion factor of kg to Gg.

### Activity data

#### Vinasse production

For these calculations SEEG used the same database compiled to calculate the N2O emissions from vinasse application to soil as fertilizer^31^.

#### Factor of N loss from leaching and runoff (FRAC_leach_)

SEEG used a N loss factor due leaching and runoff of 30% of the N in vinasse applied to soil as fertilizer^31^.

#### Emission factor (FE_5_)

For the calculation of indirect emissions of N_2_O, SEEG applied an emission factor (FE_5_) of 0.025 kg N-N_2_O per kg of N to vinasse leached and runoff after application to soil^31^.

#### Methane emissions from rice cultivation

For estimating CH_4_ emissions from rice cultivation, SEEG replicated the methodology described in IPCC^12^ according to the following equation:

$$E_{CA}=A_{C}xFE_{C}xSF_{0}xSF_{W}x10^{-9}$$

Where: E_CA_=CH_4_ emissions from rice cultivation (Gg/year); A_C_=area of rice cultivation under management C (m^2^); FE_C_=emission factor for rice cultivation fields permanently flooded without organic amendment (g/m^2^); SF_0_=factor applied to fields under organic amendment; SF_W_=factor applied for different types of water management; 10^−9^=conversion factor of g to Gg.

### Activity data

#### Area of rice cultivation (A_c_)

Data needed to estimate methane emissions from rice cultivation are the rice area under: continuous flooding, intermittent with multiple water drainage events, intermittent with single water drainage event and lowland rice floodplain. Rice cultivation area from 1990-2010 are presented at MCTI^66^, the source of which is the Economics Data Center of the Embrapa Rice and Beans^67^. This last data source also presents the up-to-date rice area.

Data for the period of 1970-1985 was obtained only for the state of Rio Grande do Sul, which has close to 60% of the rice production area in flood conditions in Brazil, from the yearbook of the Rio Grande State Rice Institute^68^.

Thus, to overcome the data limitation for the other states, SEEG used the proportion of rice area under flood condition and the total area of rice cultivation in Brazil (flooded + rainfed) according to the following steps:

Step 1: SEEG calculated the percentage (%) of rice area and their respective cultivation systems for each state (except RS) on the total area of rice (flooded + rainfed) grown in Brazil in the 1990s (1990-1999, values available in IBGE/SIDRA^29^).

Step 2: SEEG multiplied such proportions by the total area of rice (flooded + rainfed) for each state for the years 1970 to 1985, thereby estimating the state area of rice cultivated by flood systems.

#### Emission factors: integrated (FE_C_) and scale (SF_0_eSF_w_)

For the calculation of methane emissions from rice cultivation, except for the State of Rio Grande do Sul (RS), we used the integrated emission factor for the states with fields continuously flooded without organic amendments (FE_C_) of 20 g/m2, a scaling factor that varies with the applied organic amendment (SF_0_) of 1.5 and a scaling factor to take into account differences in ecosystems and water management systems (SFw)^66^.

For Rio Grande do Sul state, its specific emission factors show two integrated emission factors, one for the conventional and early soil tillage systems, with values of 41.7 g/m2 and 31.7 g/m2 CH_4_, respectively. These factors require the use of scaling factors, since they were obtained in field trials with full maintenance of organic material on top of soil during the fall and winter seasons^66^.

Additionally, due to the development of specific emission factors for the state of Rio Grande do Sul, the area cultivated with flooded rice was divided into two farming systems, conventional and early soil preparation, according to proportions defined by MCTI^66^ for the period from 1990/91 to 2009/10 crop season. For the periods of 1970-1984 and 2011-2014, SEEG used same proportions of the years of 1990 and 2010, respectively.

#### Emissions of methane, nitrous oxide, carbon monoxide and other nitrogen oxides from the burning of crop residues

To estimate of CH_4_, CO, N_2_O and NO_x_ emissions from burning crop residues (sugarcane and cotton) by state, there was a replication of the Tier 2 methodology described in MCTI^69^, given by the following equation:

$$E_{RA}=\sum^{\;}PxR_{pc}xF_{qc}xF_{C}x\left(F_{gasCH4}+F_{gasN2O}+F_{gasCO}+F_{gasNOx}\right)x10^{-6}$$

Where: *E*_*RA*_=emission of a given gas from the burning of crop residues; *P*=crop production A (kg); *R*_*pc*_=ratio straw/stalk of the crop A; *F*_*qc*_=% of the crop area A that is burned; *F*_*C*_=combustion factor; *F*_*gas*_=emission factor of methane (CH_4_), nitrous oxide (N_2_O), carbon monoxide (CO) and (other nitrogen oxides NO_x_); 10^−6^=conversion factor from kg to Gg.

### Activity data

#### Crop production and harvested area under crop residues burning

The source of data used for obtaining the sugarcane and cotton crop production and harvested area under crop residue burning for the period of 1970 and 1990-2014 was the IBGE/SIDRA^29^ database. For the period of 1975-1989, information was obtained by direct request to the IBGE (through the e-mail address: ibge@ibge.gov.br),whereas the information for the 1971-1974 period (not available at IBGE/SIDRA^29^) was obtained by linear interpolation of the 1970-1975 data. State level data of the percentage of sugarcane and cotton harvested after crop residue burning for the period of 1990-2012 area is found in MCTI^69^.

For sugarcane cropping in 2013 and 2014 the same percentages reported for 2012 (last year reported by the Brazilian government) were assumed, except for São Paulo State (SP) (the main sugarcane producer), where this information was found at the Secretariat of the Environment of the State of São Paulo^70^. On the other hand, since in 1990 all sugarcane areas were submitted to the burning process before harvesting in Brazil, we also considered 100% of crop residue burning for the period of 1970-1989 for all Brazilian states.

In the case of cotton growing, burning of crop residues was mandated by law in order to avoid crop diseases. However, technology improvement in cotton crop management over the years made it possible to prevent disease. Because of that, in the 90’s the burning of cotton residues began to decrease, coming to an end in 1995 (ref. 69).

Therefore, the MCTI^69^ considered a decrease from 50% to zero as a fraction of cotton area burned after harvesting in the period of 1990-1995. For the SEEG, the same rationale was applied to estimate these burned areas for the period of 1970-1989, but in a reverse fashion, according to the following steps:

Step 1: in 1990, 50% of the cotton harvested area was burned by the Brazilian state producers, with uniform reduction to 0% until 1995.

Step 2: the SEEG used the same decreasing ratio in a reverse order to the years before 1990 until getting 100% of the cotton area burned after harvesting (1985).

Therefore, until 1994 the emissions of GHG from the burning of crop residues of sugarcane and cotton were considered. After that, there were only GHG emissions from burning of sugarcane residues.

#### Crop fractions and emission factors of CH_4_, N_2_O, CO and NOx

The straw/stalk ratio of the sugarcane and cotton crops, the combustion factors of their residues and the CH_4_, N_2_O, CO and NOx emissions factors are available in MCTI^69^.

#### Emissions and removals of carbon dioxide from soil carbon stocks variation (not inventoried emissions and removals)

The variation in soil carbon stocks refers to the emissions and removals (sequestration) of CO_2_ from the soil organic matter. As commented above, this variation is not reported in the national inventories^8–10^. Therefore, the SEEG made an exercise of calculating this variation for soils used by Brazilian agriculture and categorized them as Non-National Inventory (NCI).

For the estimation of the variation of soil carbon stocks (C_soil carbon stock_), CO_2_ emissions and removals in SEEG were calculated according to the formula below, which is a function of soil use and management soil and their respective factors of emission and removal.

$$C_{soilcarbonstock}=\sum^{\;}\left[\left(A_{pastdegrad}xF_{CO2emission})+(A_{pastwellmanag}xF_{CO2removal})+(A_{ilpf}xF_{CO2removal})+(A_{croppingCT}xF_{CO2emission})+(A_{croppingNT}xF_{CO2removal})+(A_{plantedforest}xF_{CO2removal}\right)\right]$$

Where: $C_{soilcarbonstock}=$ soil carbon stock variation; $A_{pastdegrad}$=area of degraded pasture (ha); $A_{pastwellmanag}$=area of well managed pasture (ha); $A_{ilpf}$=area of crop-livestock-forest integrated systems (ha); $A_{croppingCT}$=cropping area under conventional tillage (ha); $A_{croppingNT}$=cropping area under no-tillage (ha); $A_{plantedforest}$=area of planted forest (ha); $F_{CO2emission}$=emission factor of carbon dioxide (tCO_2_ ha-1); *F*_*CO*2* removal*_=removal factor of carbon dioxide (tCO_2_ ha-1).

### Activity data

The basic activity data for the calculation of the variation of soil carbon stocks is the area used by agriculture, its uses and management. Due to the high variation and uncertainties of these data, as well as their respective emission and removal factors, the SEEG was limited here to presenting an exercise for this estimate, based on consultations with specialists in several areas and a bibliographic survey. After this survey, the SEEG divided the Brazilian agricultural land into the following uses and managements:

1. Crops under conventional tillage (SPC) and no-tillage (SPD) systems: it was considered that the soil of the conventional plantation crops emits CO_2_ and the soil of the no-till crops removes and sequesters CO_2_^71, 72^. These CO_2_ emission and removal estimations were made for the years 1990 to 2015 at the national level based on data from CONAB^73^ and FEBRAPDP^74^ as follows (Supplementary Table S1):
2. 1.1 CONAB^73^ presents data for the size of the agricultural area in Brazil (1990-2015) and FEBRAPDP^74^ provides the agricultural area under SPD in Brazil from 1973-2006 and 2012.
3. 1.2 The area under SPD for 2007-2011 was obtained by interpolating the 2006 and 2012 data. And the historical correlation between the total and under SPD area of 1990-2012 was used to project the SPD area of 2013- 2015.
4. 1.3 It was assumed that the SPC area is the difference between the SPD area of the total agricultural area.
5. Commercial Planted Forests: The soil of commercial planted forest areas has been considered to remove (sequester) CO_2_. These CO_2_ removals estimate were made for the years 1990 to 2015 for national and state levels based on data from IBA^75^ and ABRAF^76, 77^ as follows (Supplementary Table S2):
6. 2.1 IBA^75^ provides state data on commercial planted forest area from 2006 to 2015, while ABRAF^76, 77^ presents estimations for the area planted forests in Brazil in 1990, 2000, 2004 and 2005.
7. 2.2 The total Brazilian area values for 1991-1999 and 2001-2005 were obtained by interpolating the data for 1990-2000 and 2000-2006, respectively. Subsequently, this total estimated area was allocated in the Brazilian states by the historical state proportion in the area of national planted forests of the years 2006-2009. There was also an area with planted forests that could not be allocated (named NA) in the states, because the activity data itself has not been allocated in the IBA^75^.
8. Pastures: due to lack of information (even from consulting specialists), CO_2_ emission and removal estimations were only made for the year 2015 at the national level. After consulting specialists, the total area of pasture was estimated at 175 mi ha, which was divided into 3 conditions: Stable, Degraded and Well Managed (Supplementary Table S3). The soil of degraded pastures is considered to emit CO_2_; the soil of improved pastures removes CO_2_ and stable pastures present soil carbon change equal to zero (emission equals removal).
9. Forest-livestock-forest integration (ILPF): due to lack of information (even from consulting specialists), an estimate was made only for the year 2015 at the national level, based on the ILPF area published by Embrapa^78^. The soil of the areas under ILPF was considered to remove CO_2_ (Supplementary Table S3).

#### Emission and removal factors

All CO_2_ emission and removal factors used in SEEG for the four agriculture use categories described above are shown in Supplementary table S3.

### Calculation of the GHG emissions: Energy Sector

Energy production and consumption GHG emissions occur through two different processes: (i) fuel combustion and (ii) fugitive emissions.

In fuel combustion, fuel chemical energy content is converted into heat. End-use equipment direct consumption (ovens, heaters, dryers, etc.) and conversion into mechanical or electrical energy (thermal electricity generation and mobile sources) are two possible ways for this heat. During combustion, carbon (C) stored in the fuel is oxidized and released as carbon dioxide (CO_2_). There are also relatively minor emissions of other gases resulting (i) from incomplete fuel combustion - methane (CH_4_), carbon monoxide (CO) and non-methane volatile organic compounds (NMVOCs) -, and (ii) from nitrogen (N_2_) oxidation, mainly original from air consumption in combustion, depending on process temperature - nitrogen oxides (NO_x_) and nitrous oxide (N_2_O)^79, 80^.

Fugitive emissions are intentional and unintentional releases that occur during processes of coal, oil and natural gas production. These emissions are related to fuel extraction, storage, processing and product transportation activities^81, 82^.

Coal geological formation process, which occurs over millions of years, generates methane (CH_4_) which remains stored within the solid mineral. Fugitive emissions occur when coal is subjected to lower pressure, which happens during mine excavation. Furthermore, there are also CO_2_ emissions resulting from spontaneous combustion in coal deposits and dump piles.

In the oil and natural gas industry, fugitive emissions occur in three activity sets as listed below^82^:

- *Oil and natural gas extraction and production*: venting, flaring, methane flash tanks, glycol dehydration process, CO_2_ removal (MEA and DEA columns), pig passage through pipelines, fugitive emissions in pipeline components such as connectors, valves and others, drilling activities, oil spills in channels, depressurization and tanks and vessels cleaning;
- *Oil refining*: fluid catalytic cracking (FCC) unit regenerators, hydrogen generation units (GHU), fugitive emissions in pipeline components such as connectors, valves and others, flaring, venting, glycol dehydration process and pig passages in pipelines;
- *Natural gas transport in pipelines*: depressurizing lines, fugitive emissions in pipeline components such as connectors, valves and others, leaks in pipelines, flaring, methane flash tanks and pig passage through pipelines.

#### Energy sector emissions estimation scope and structure in SEEG

Energy Sector emissions scope in SEEG is the same as recommended by the Intergovernmental Panel on Climate Change (IPCC) in its guidelines for GHG emissions^12^.

As for emission-generating activities, they include primary energy sources exploration and extraction; energy conversion processes (oil refineries, biofuels production units, power generation units, etc.) and energy end-use in mobile or stationary applications. Emission estimation methods defined a structure for results presentation in five levels:

- Emission type: fuel combustion and fugitive emissions;
- Energy source: primary energy sources whose production is responsible for emissions and energy sources for end-use;
- Activity level 1: energy final consumption sectors as defined by the Brazilian National Energy Balance – BEN (examples: transport, industry, agriculture), electricity generation and fuel production;
- Activity level 2: previous level breakdown;
- Activity level 3: previous level breakdown.

Supplementary Figure S1 shows the structure used and the contents of each level in the structure. It also represents branches or combinations occurring for each element or group of elements.

Emissions from the following fuel consumption in iron and steel, ferroalloys, non-ferrous metal and other metallurgy production are not accounted for in the Energy Sector: coal coke, petroleum coke, steam coal 6000, steam coal 5900 and charcoal. Such fuels are reactants in a thermal reduction process, not a simple burning; therefore, related emissions are considered in Industrial Processes and Product Use^12^.

CO_2_ emissions from biomass combustion (wood, charcoal, crop residues, alcohol, sugarcane bagasse, biogas and other biomass products) are not accounted for in the Energy Sector as these emissions are offset by CO_2_ absorption during photosynthesis process. Other greenhouse gases emissions, are accounted for, as has been done for fossil fuels^12^.

#### Emissions estimation methodology: National emissions

SEEG’s national fuel combustion emission estimations use two general methods, one for CO_2_ and another for the other gases (CH_4_, N_2_O, CO, NO_x_ and NMVOC) for all activities other than road and air transport. Emission estimation for these two transport modals required more detailed information and more specific methods. This section will explain these four methods and the three specific methods for fugitive emission estimation (CH_4_ from coal mining, CO_2_ from coal mining and GHG from oil and natural gas industry).

#### CO_2_ fuel combustion emissions: general method, bottom-up approach

CO_2_ emission estimation used the bottom-up approach. This approach allows the results obtained to be detailed by energy end-use sectors or energy transformation processes and by energy source consumed.

CO_2_ annual emissions for of the energy sources are estimated from the following equation:

$$E_{CO_{2},f,a}=Cons_{f,a}*Ef_{CO_{2},f}$$

Where: *E*_*CO*2,*f*,*a*_=CO_2_ annual emissions, detailed by fuel (f) and activity levels (a) 1, 2 and 3 (kgCO_2_/year); *Cons*_*f*,*a*_=Fuel annual final energy consumption in each sector, or fuel amount used in transformation centers, detailed by fuel (f) and activity levels (a) 1, 2 and 3 (TJ/year); *Ef*_*CO*2,*f*_=Carbon dioxide emission factor per energy unit contained in fuel, detailed by fuel (f) (kgCO_2_/TJ).

As for activity data, BEN^65^ was the data source for Cons variables. The key activity variable, Cons, is fuel final energy consumption and its use in energy transformation processes, in line with the classification adopted^65^. This data is available as a spreadsheet at the Brazilian Ministry of Mines and Energy website.

The emission factors applied (Fe_CO2_) are the same published by the Brazilian Ministry of Science, Technology and Innovation in its fuel combustion GHG emissions – bottom-up approach reference report^79, 80^. The Supplementary figure S2 represents the data processing used sequence.

#### CH_4_, N_2_O, CO, NO_x_ and NMVOC fuel combustion emissions: general method

Carbon dioxide (CO_2_) emission estimation depends on only a few fuel properties to have reasonable accuracy. Methane (CH_4_), nitrous oxide (N_2_O), carbon monoxide (CO), nitrogen oxides (NO_x_) and non-methane volatile organic compounds (NMVOCs) emission estimations, on the other hand, are more dependent on combustion process characteristics. Therefore, to estimate these emissions, the fuel combustion process must be detailed by end-use (process heat, driving force, direct heating or illumination) and by technology (heaters, boilers, engines, ovens and dryers). For that purpose, the following methodology defines "allocation coefficients", as described below:

$$E_{f,a,u,t}^{g}=Ef_{f,a,u,t}^{g}*C_{f,a}*X_{f,a,u}*Y_{a,u,t}$$

Where: $\;E_{f,a,u,t}^{g}=$Gas g emission from fuel f consumption, in activity a, by end-use u, using technology t (kg_gas_/year); $Ef_{f,a,u,t}^{g}=$Gas g emission factor from fuel f consumption, in in activity a, by end-use u, using technology t (kg_gas_/TJ); *C*_*f*,*a*_ =Fuel f consumption, in activity a (TJ/year); *X*_*f*,*a*,*u*_ =End-use allocation coefficient for fuel f consumption, in activity a, by end-use u (%); $Y_{a,u,t}=$Technology allocation coefficient which stands for how much energy in end-use u, for activity a, is attended by technology t (%).

Thus, as in CO_2_ emissions estimations, the data source for variable fuel consumption (C) was BEN^65^.

The fuel combustion CO_2_ emissions – bottom-up approach reference report published by the Brazilian Ministry of Science, Technology and Innovation^79, 80^ was also the information source for this set of emission factors. Brazilian Useful Energy Balance (BEU)^83^, contains end-use allocation coefficients (X). BEU has information for these variables for 1983, 1993 and 2003. The following simplifications were made to obtain values for the remaining years between 1970 and 2015:

- For periods 1984-1992, 1994-2002, coefficients were obtained from linear interpolation considering available data for 1983, 1993 and 2003;
- Between 1970 and 1982, the same coefficients as for 1983 were used;
- Between 2004 and 2015, the same coefficients as for 2003 were used.

Technology allocation coefficients (Y) are the same published by the Brazilian Ministry of Science, Technology and Innovation in its fuel combustion CO_2_ emissions – bottom-up approach reference report^79, 80^. This reference report contains information up to 2010, for further periods (2011-2015), the methodology applied does not consider any change in technology allocation coefficients. represents the data processing used sequence.

#### Road transport fuel combustion emissions

Road transport energy consumption data available in the Brazilian National Energy Balance^65^ is detailed only by fuel type. Therefore, using this data set and general methods presented above, it is not possible to estimate emissions detailed by vehicle type. Also, engines technological development, vehicles usage profile by category and age, and emission factor increase because of deterioration during vehicle use are some of the very important characteristics when estimating CH_4_, N_2_O, CO, NO_x_ and NMVOCs emissions from road transport.

SEEG estimations for this activity reproduce the Brazilian Ministry of Environment road transport atmospheric emission inventory methodology^84^. This inventory estimates atmospheric emissions by road vehicles across the country, from 1980 to 2012. The Inventory estimates CO, NO_x_, NMVOC (non-methane hydrocarbons and aldehydes), CO_2_, CH_4_ and N_2_O emissions^84^.

The methodological procedure of this inventory will not be described in detail in this article because of its extent; however, it is consistent with IPCC Guidelines Tier 2 methodology^12^ and the two most important general equations applied will be shown below.

i) *Fuel consumption* data is the most important variable for calculating *CO*_*2*_ *emissions* as presented in a previous section. Thus, for CO_2_ emission estimation to be detailed by vehicle type, fuel consumption must be as well. For each fleet vehicle type, in a specific calendar year, fuel consumption is estimated from the following equation:

$$C_{t}=\sum_{m}Fl_{t,m}\times U_{t,m}/Fe_{t,m}$$

Where: $C_{t}=$Annual fuel consumption for vehicle type t (L/year); $Fl_{t,m}=$Current fleet in the calendar year, for vehicle type t, produced in model year m (number of vehicles); $U_{t,m}=$Vehicle usage in terms of distance travelled in the calendar year, for vehicle type t, produced in model year m (km/year); $Fe_{t,m}=$Fuel economy for vehicle type t, produced in model year m (km/L).

Fuel consumption estimated by the inventory is adjusted for each fuel type, based on road transport fuel consumption published in BEN^65^, through annual correction factors of the usage variable (U). Therefore, the total CO_2_ road emission estimation (totaling all vehicle types) matches the estimation using the method described in the previous section “*CO*_*2*_ *fuel combustion emissions: general method, bottom-up approach*”.

ii) To estimate *non-CO*_*2*_ *gas emissions*, the following equation is applied for each fleet vehicle type, in a specific calendar year:

$$E_{t}^{g}=\sum_{m}Fl_{t,m}\;\times U_{t,m}\;\times Ef_{t,m}^{g}$$

Where: $E_{t}^{g}=$Gas g emission annual rate for vehicle type t (g_gas_/year); $Ef_{t,m}^{g}=$Gas g emission factor, for vehicle type t, produced in model year m (g_gas_/km).

#### Aviation fuel combustion emissions

Applying the equation described in previous section “*CO*_*2*_ *fuel combustion emissions: general method, bottom-up approach*”, it is possible to estimate CO_2_ emissions from aviation. However, SEEG’s methodology applies a different method for estimating CH_4_, N_2_O, CO, NO_x_ and NMVOC emissions.

Brazilian National Civil Aviation Agency (ANAC) published its civil aviation atmospheric emission inventory with an IPCC Tier 3A approach level of detail for CO, NO_x_ and NMVOC emissions and for fuel consumption during the period between 2005 and 2013 (ref. 85). The dataset results obtained from this inventory made it possible to estimate an implicit emission factor for these gases during this period. Combining the aviation fuel consumption reported by the Brazilian National Energy Balance^65^ and these implicit emission factors, it was possible to estimate CO, NO_x_ and NMVOC emissions for the 2005-2013 period. The 2005 implicit emission factor was applied for the 1970-2004 period and the 2013 one for 2014 and 2015.

CH_4_ and N_2_O emission factors applied are the same published in the fuel combustion CO_2_ emissions – bottom-up approach reference report published by the Brazilian Ministry of Science, Technology and Innovation^79, 80^. The equation below describes estimation for each gas g and fuel type f:

$$Emission_{g,f}=FuelConsumption_{f}\times EmissionFactor_{g,f}$$

#### Coal mining CH_4_ fugitive emissions

The methodology applied in SEEG to estimate CH_4_ fugitive emissions from coal mining is the same presented in the reference report on coal mining and treatment for GHG fugitive emissions published by the Brazilian Ministry of Science, Technology and Innovation^81^. The following equation represents this methodology:

$$E_{CH_{4}}=\sum_{m}\sum_{u}\left(P_{m,u}\times Ef_{m}\right)$$

Where: $\;E_{CH_{4}}=$Annual CH_4_ emissions (tCH_4_/year); $P_{m,u}=$Run-of-mine (ROM) coal production in federative unit u and by coal mine type m (t coal/year); $Fe_{m}=$CH_4_ emission factor in terms of coal production, by coal mine type m (tCH_4_/t coal).

The MCTI^81^ presents ROM coal production data between 1990 and 2011. For the 2012-2015 period, the Brazilian Coal Association website was the information source. By pooling ABCM ROM coal (1990-2015) and BEN^65^ processed coal (1970-2015) production data, it was possible to estimate ROM annual coal production between 1970 and 1989 by assuming the ratio between ROM coal and processed coal production in 1990 to be the same during 1970-1989 period.

Like the estimations in MCTI^81^, the same mine profile of coal production mines by state in 2011 was applied for the following years (2012-2015). The 1990 mine profile was used for previous years (1970-1989).

The MCTI reference report^81^ is also the information source for CH_4_ emission factors. These factors are: 10.90 m^3^ of CH_4_ per coal ton for underground mines and 0.35 m^3^ of CH_4_ per coal ton for surface mines. Methane density adopted was 670 g/m^3^.

#### Coal mining CO_2_ fugitive emissions

For coal mining CO_2_ fugitive emissions, the methodology adopted in the reference report on coal mining and treatment for GHG fugitive emissions reference report published by MCTI^81^ is not entirely available.

Therefore, to estimate emissions in periods 1970-1989 and 2013-2015, SEEG applied the methodology presented MCTI^10^. This method applies the following linear correlation:

$$E_{CO_{2},t}=0,1783P_{ROM,t}-514126$$

Where: $\;E_{CO_{2},t}=$CO_2_ emissions in year t (tCO_2_/year); *P*_*ROM*_ =ROM coal production in year t (t coal/year).

For the period between 1990 and 2012, SEEG presents the emission data published in MCTI reference report^81^.

#### Oil and natural gas industry fugitive emissions

The dataset needed to reproduce the methodology presented in the oil natural gas industry GHG fugitive emissions reference report published by the MCTI^82^ is not currently publicly available to the public. Therefore, SEEG applied the linear correlations presented by MCTI^10^ to estimate emissions in 1970-1989 and in 2013-2015.

Oil and natural gas extraction and production CO_2_ fugitive emissions:

$$E_{CO_{2},t}=47,452P_{t}-359744$$

Where: $E_{CO_{2},t}=$CO_2_ emissions in year t (tCO_2_/year); $P_{t}=$Oil and natural gas production in year t (ktoe/year)

Oil refining CO_2_ fugitive emissions:

$$E_{CO_{2},t}=93,948P_{t}-1255841$$

Where: $E_{CO_{2},t}=$CO_2_ emissions in year t (tCO_2_/year); $P_{t}=$Oil processed in oil refineries in year t (ktoe/year)

CH_4_ and N_2_O emissions between 1970 to 1989 were estimated maintaining the ratio between the CO_2_ emissions and the specific gas emissions, as shown by the equation below, and adopting 1990 as the base year.

$$E_{g}^{t}=E_{CO_{2}}^{t}\times \frac{E_{g}^{1990}}{E_{CO_{2}}^{1990}}$$

Where: $E_{g}^{t}=$Gas g emissions (N_2_O or CH_4_) in year t; $E_{CO_{2}}^{t}=$CO_2_ emissions in year t; $E_{g}^{1990}=$Gas g emissions (N_2_O or CH_4_) in 1990; $E_{CO_{2}}^{1990}=$CO_2_ emissions in 1990

The equation above was also applied to 2013-2015 emission estimations, but adopting 2012 as the base year instead of 1990.

#### Emissions estimation methodology: Bunker emissions

SEEG database provides information regarding international aviation and water-borne navigation emissions associated with Brazilian fuel combustion. The 2006 IPCC Guidelines^12^ consider these as bunker fuel emissions.

International water-borne navigation emissions were estimated by the methodology described in sections CO_2_ fuel combustion emissions: general method, bottom-up approach and CH_4_, N_2_O, CO, NO_x_ and NMVOC fuel combustion emissions: general method given that the Brazilian National Energy Balance contains data regarding fuel consumption for international water-borne navigation (bunker diesel oil and bunker fuel oil).

The methodology presented in section *Aviation fuel combustion emissions* given that the Brazilian National Energy Balance contains data regarding fuel consumption for international aviation (jet kerosene bunker) and the civil aviation atmospheric emission inventory published by ANAC^85^ also has data regarding emissions related to international aviation.

#### Emissions estimation methodology: Subnational emissions

Energy sector emissions in SEEG database are also detailed by federative units. The procedure adopted by SEEG methodology was to distribute national emissions by considering official data regarding the most important emission drivers detailed by federative units, whenever possible (Supplementary Table S4). The proposed methodology allocated 91.7% of energy sector emissions in 2015.

Coal mining fugitive emissions estimations are already detailed by federative unit, since activity data and emission factors are also detailed by them. Therefore, national emissions reported from this activity are the total amount of subnational emissions.

All other energy sector emission estimations at subnational level (fuel combustion and oil and natural gas industry fugitive emissions) resulted from the following equation:

$$e_{g,f,a,u,t}=X_{f,a,u,t}\times E_{g,f,a,t}$$

Where: $e_{g,f,a,u,t}=$Gas g emission from fuel f consumption, in activity a, in federative unit u, in year t (kg_gas_/year); $X_{f,a,u,t}=$Allocation factor from fuel f consumption, in activity a, in federative unit u, in year t (%); $E_{g,f,a,t}=$Gas g emission from fuel f consumption, in activity a, in year t (kg_gas_/year).

For most fuel combustion activities, X is the ratio between fuel f consumption, in activity a, in federative unit u, in year t and national fuel f consumption, in activity a, in year t (C_f,a,u,t_/C_f,a,t_). Information sources for subnational fuel consumption data are detailed in the Table 1. For Fuel Production related to Oil and Natural Gas Industry, other drivers were applied to allocate subnational emissions: (i) annual oil volume processed in oil refineries and (ii) annual oil and natural gas production. The oil and natural gas industry fugitive emissions allocation method also used these two variables.

#### Calculation of the GHG emissions: Industrial Processes and Product Use (IPPU) sector

Industrial activities can generate air emissions from fuel combustion (heat or electricity generation), for waste disposal (industrial wastewater treatment and waste incineration) and by chemical and/or physical materials processing.

For each of these three processes types, emissions occur under a wide variety of specific features, such as the product itself, raw material consumption, technological route used in production, industrial plant equipment and efficiency levels.

SEEG adopts Intergovernmental Panel on Climate Change (IPCC) recommendations^12^, in which "Industrial Processes and Product Use (IPPU)" category estimations take into account only emissions that occurred in chemical or physical material transformation. Thus, fuel combustion emissions are accounted for in "Energy Sector", and waste disposal emissions in "Waste Sector".

The emission estimation groupings are listed below:

- Metal production: pig iron and steel, ferroalloys, aluminum, magnesium and other non-ferrous metal production;
- Mineral products: lime, cement and glass production and soda ash consumption;
- Chemical industry: adipic acid, phosphoric acid, nitric acid, acrylonitrile, ammonia, caprolactam, calcium carbide, vinyl chloride, ethylene, methanol, carbon black, ethylene oxide, calcined petroleum coke and other petrochemical production;
- Hydrofluorocarbons (HFCs) emissions;
- Sulphur hexafluoride (SF_6_) use in electrical equipment;
- Non-energy products from fuel and solvent use.

#### Industrial processes and product use emissions estimations scope and structure

Supplementary Figure S4 shows the emissions estimation tree structure from industrial processes and product use containing industrial activities, industrial processes and product uses, products and input groupings and emitted gases.

SEEG’s latest version contains annual IPPU sector emissions between 1970 and 2015, at national and subnational levels. Inventoried gases are carbon dioxide (CO_2_), methane (CH_4_), nitrous oxide (N_2_O), carbon monoxide (CO), non-methane volatile organic compounds (NMVOC), nitrogen oxides (NO_x_), perfluorocarbons (CF_4_ and C_2_F_6_), hydrofluorocarbons (HFC-23, HFC-32, HFC-125, HFC-134a, HFC-143a, HFC-152a and sulphur hexafluoride (SF_6_).

#### Emissions estimation methodology: Metal production

*Pig iron and steel production*

Pig iron and steel production emissions occur in blast furnaces by fuel consumption (charcoal, petroleum coke, coal coke and coal) as reducing agents and by carbonate flux consumption (limestone and dolomite). A simplified representation of the pig iron and steel production process and CO_2_ emissions accounted in IPPU sector is shown in Supplementary Figure S5.

#### Reductant fuel consumption

SEEG methodology adopted the same procedure presented in the reference report published by the Brazilian Ministry of Science, Technology and Innovation on GHG emissions in metal production^86^. Emissions of CO_2_ occur in a similar way to fuel combustion in furnaces, but discounting the stored carbon in the metals produced. Other gas emission estimations use the same procedure applied for fuel combustion in furnaces described in the energy sector methodology. The following formulations represent the method applied for each gas:

$$E_{CO_{2}}=\left(C*Ef_{CO_{2}}\right)-\left(P*Sto*\frac{44}{12}\right)$$

Where: $E_{CO_{2}}=$CO_2_ emissions (CO_2_ tons/year); C=Fuel consumption (TJ/year); $Ef_{CO_{2}}=$CO_2_ emission factor by fuel energy unit (CO_2_ tons/TJ); P=Pig iron or steel production (tons/year); Sto=Carbon mass percentage stored in pig iron or steel (%C); $\frac{44}{12}=$CO_2_ and C molar masses relation.

$$E_{g}=C*Ef_{g}$$

Where: $E_{g}=$Gas *g* – CO, CH_4_, NO_x_, N_2_O or NMVOC – emissions (kg/year); C=Fuel consumption (TJ/year); $Ef_{g}=$Gas *g* – CO, CH_4_, NO_x_, N_2_O or NMVOC – emission factor (kg/TJ).

The Brazilian National Energy Balance (BEN)^65^ was the information source for fuel consumption data. The emission factors applied are the same published by MCTI^79, 80^. Stored carbon mass percentages in steel (4%) and pig iron (1%) were also obtained from the reference report on GHG emissions in metal production^86^.

Steel production data for 1970 and 1971 were obtained from spreadsheets provided by the Ministry of Mines and Energy. For the period between 1972 and 2009, production was provided by the Brazilian Steel Institute on request. For between 2010 and 2015, this dataset is available at the IABr website.

Concerning charcoal consumption, CO_2_ emissions are not accounted for in Industrial Processes and Product Use Sector, as it is considered that these emissions are offset by CO_2_ absorption in photosynthesis, which generated biomass, as recommended by IPCC^12^. Other GHG emissions are estimated as usual, such as for fossil fuels.

Carbon content on fossil reductant fuel was the driver for subnational level CO_2_ emission allocation. Five federative units represented 96% of Brazilian steel production in 2015; for these units (MG, RJ, SP, ES and RS) it was possible to allocate emissions. Therefore, a small amount of emissions remains not allocated.

#### Carbonates consumption

Limestone and dolomite are fluxing agents employed in blast furnaces to make slag more fluid and remove impurities in the alloy produced. As shown in Supplementary Figure S5, in blast furnaces at high temperatures, these minerals decarbonize, which generates CO_2_ emissions.

As published by the Brazilian Ministry of Science, Technology and Innovation on its GHG emissions related to mineral products reference report^86^, emission estimation follows the equation below:

$$E_{i}=C_{i}*Ef_{i}$$

Where: $E_{i}=$Carbonate *i* consumption CO_2_ annual emissions in blast furnaces (tCO_2_/year); $C_{i}=$Carbonate *i* annual consumption in blast furnaces (carbonate tons/year); $Ef_{i}=$Carbonate *i* consumption CO_2_ emission factor (tCO_2_/carbonate tons).

Emission factors are based on carbonates calcination reaction stoichiometry: 0.440 tCO_2_/t limestone and 0.477 tCO_2_/t dolomite^86^. These values consider that the ores are composed exclusively by CaCO_3_ (limestone) and CaCO_3_.MgCO_3_ (dolomite).

This reference report was also the information source for consumption of each mineral between 1990 and 2012 (ref. 86). Dolomite consumption in blast furnaces in 2013 was obtained from the Metallurgical Sector Statistical Yearbook 2015 (ref. 87). The statistical yearbook also presents the consumption of limestone in the steel industry, which includes the amount intended for captive lime production. To estimate this mineral consumption only as a flux in blast furnaces, it was assumed that the captive production of lime in 2012 remained constant. Limestone amount consumed in lime production was obtained from the stoichiometric ratio 100.09 g CaCO_3_/56.08 g CaO; discounting the consumption data reported by the statistical yearbook^87^. Therefore, it was possible to estimate consumption exclusive in blast furnaces.

For carbonate consumption between 1970 and 1989 the estimation methodology used the relationship between limestone or dolomite consumption and steel production for 1990 as the following equation describes (the *i* index indicates the carbonate type and the *X* index indicates the estimation year).

$$C_{i}^{X}=\frac{C_{i}^{1990}}{P_{steel}^{1990}}*P_{steel}^{X}$$

Where: $\;C_{i}^{X}=$Carbonate *i* consumption in blast furnaces, in year *X* (carbonate tons/year); $P_{steel}^{X}=$Steel production, in year *X* (steel tons/year).

Given that there were no publicly available data regarding dolomite and limestone consumption between 2013 and 2015, 2012 data were applied in calculations for these years. Also, there were no publicly available data regarding subnational consumption; thus these emissions were not allocated.

#### Aluminum production

CO_2_ emissions from aluminum production are those resulting from electrolytic reduction of alumina (Al_2_O_3_) in metallic aluminum (Al) using a carbon anode (C), the latter generally derived from petroleum coke. Furthermore, the electrolytic solution contains cryolite and aluminum fluoride (fluxes), substances that, through a phenomenon called anode effect, generate fluorocarbon emissions (CF_4_ and C_2_F_6_). Supplementary Figure S6 represents these greenhouse gas emissions.

The Brazilian Ministry of Science, Technology and Innovation in its metal industry GHG emissions reference report^86^ presents aluminum production emission results from different methodologies (depending on available information from each production site in the country).

The MCTI reference report presents specific implicit emission factors for each production route (Prebaked Anode or Soderberg) which would represent the national production emission intensity. SEEG estimations applied these factors as shown in the following equation.

$$E_{g,p,i}=P_{p,i}*Ef_{g,i,t}$$

Where: $\;E_{g,p,i}=$Aluminum production gas *g* emissions, in plant *p*, in year *i* (t gas/year); $P_{p,i}=$Aluminum production, in plant *p*, in year *i* (t aluminum/year); $Ef_{g,i}=$Aluminum production gas *g* emission factor, in year *i* by technological route *t* (t gas/t aluminum).

Emission factors used for the period between 1990 and 2010 are the same as those presented by the MCTI reference report for the three gases: CO_2_, CF_4_ and C_2_F_6_. For the 1970-1989 period, factors reported for 1990 were used and, similarly, 2010 factors were used for the 2011-2015 period.

For the entire estimation time frame (1970-2015), production from each aluminum plant was obtained from personal communication with the Brazilian Aluminum Association. Therefore, it was possible to estimate subnational level emissions.

#### Magnesium production

Brazilian metallic magnesium production is based on a thermal reduction process and uses dolomite as an input material. All production is performed by Rima Industrial SA in Minas Gerais. Rima’s activities began in 1987, but it was not possible to collect data related to magnesium production for the period prior to 1990. Thus, the emissions between 1987 and 1989 were not estimated.

#### Dolomite consumption

During the thermal reduction process, dolomite suffers a chemical reaction similar to calcination, generating CO_2_ emissions. As presented by MCTI^88^, the emission estimation is made by multiplying magnesium production and default emission factor 5.13 tCO_2_/t magnesium^12^.

The MCTI reference report is the information source for production between 1990 and 2011. For period 2012-2015, see Rima’s reports to the United Nations Framework Convention on Climate Change under the Clean Development Mechanism (CDM)^89^ Project 2486.

#### Sulphur hexafluoride consumption

At the end of thermal reduction process, magnesium is liquid. To avoid metal oxidation, a "cover gas" is used as protection. This gas usually leaks into the atmosphere and all the gas used in the process is emitted^88^.

Rima used to utilize sulphur hexafluoride (SF_6_) as cover gas and the MCTI reference report shows the emissions from this consumption between 1990 and 2009. Due to a greenhouse gas emissions control program, metal protection began to be made with sulfur dioxide (SO_2_). Therefore, this activity of SF_6_ emissions stopped in 2010.

#### Ferroalloy and other non-ferrous metal production

Ferroalloy and other non-ferrous metal (except aluminum and magnesium) production emissions are also from consumption of fuels as reducing agents in blast furnaces (charcoal, petroleum coke, coal coke and mineral coal). Therefore, methodologies and information sources used to produce these estimations are the same as those presented in the pig iron and steel production section.

Stored carbon amount is negligible both in ferroalloys and in other non-ferrous metal. Thus, production of these metals does not provide data necessary for emissions estimations^88^.

It should be noted that petroleum coke use related emissions in other non-ferrous metal production is not estimated from all consumption reported by Brazilian National Energy Balance, given that part of this consumption is for carbon (C) anode production in metallic aluminum production. Consumption actually intended for other non-ferrous metal production furnaces between 1990 and 2012 was obtained from the metal industry GHG emissions reference report^88^. For years between 1970 and 1989, the following equations represent how petroleum coke consumption in other non-ferrous metal production furnaces was estimated.

$$C_{coke,Al}^{X}=\frac{C_{coke,Al}^{1990}}{P_{Al}^{1990}}*P_{Al}^{X}$$

$$C_{coke,other}^{X}=C_{coke,total}^{X}-C_{coke,Al}^{X}$$

Where: $C_{coke,Al}^{X}=$Annual petroleum coke consumption in aluminum anode production in year X (ktoe/year); $P_{Al}^{X}=$Aluminum production in year X (t aluminum/year); $C_{coke,other}^{X}=$Annual petroleum coke consumption in other non-ferrous metal production in year X (ktoe/year); $C_{coke,total}^{X}=$Annual petroleum coke consumption in BEN’s "Non-ferrous and Other Metallurgy Products" category in year X (ktoe/year).

SEEG methodology assumed that the ratio between petroleum coke consumption as the anode in aluminum production and aluminum production remained constant in the years for which this value is not known. To estimate consumption for 2013-2015, the procedure adopted was the same, but the terms related to 1990 in the equation were replaced for 2012 as the reference year.

The majority of subnational emissions related to these activities was not estimated. The only exception was ferroalloy production emissions in São Paulo and Minas Gerais federative units.

#### Emissions estimation methodology: Mineral products

This section presents methodology and data necessary for CO_2_ emissions estimations related to four cases involving mineral products: cement production, lime production, glass production soda ash and consumption.

The carbon emitted as CO_2_ in these activities was present as the carbonate anion (*CO*_3_^2−^) in substances like limestone, dolomite and soda ash, for example. The following chemical reactions (limestone and dolomite thermal calcination) illustrate these emissions:

CaCO_3_ + heat → CaO + CO_2_

MgCO_3_.CaCO_3_ + heat → MgO.CaO + 2CO_2_

#### Cement production

Cement production related emissions are associated with limestone and dolomite calcination (CaCO_3_ and CaCO_3_.MgCO_3_, respectively) in cement kilns where these minerals are transformed into lime (a mixture of CaO and MgO) that is part of clinker, a raw material for cement production. Carbon dioxide (CO_2_) is another product in this reaction.

Emissions from fuel combustion in clinker kilns are not accounted are reported in Energy Sector in Cement Industry subsector. Supplementary Figure S7 illustrates emissions accounted and represents cement production process.

CO_2_ emission estimation used emission factors based on clinker calcium oxide (CaO) and magnesium oxide (MgO) contents, on additives amount used in cement production (slag, fly ash, pozzolan and CKD) and on organic carbon content in carbonates.

Since these data are not available on MCTI reference report, a simplified methodology based on emission factors regarding clinker production was adopted. The following equation represents SEEG’s methodology to estimate emissions for each year in the timeframe:

$$E_{CO_{2}}=Prod_{cement}*X_{clinker}*Ef_{clinker}$$

Where: $E_{CO_{2}}=$Cement production annual CO_2_ emissions (tCO_2_/year); $Prod_{cement}=$Cement annual production (t cement/year); $X_{clinker}=$Cement clinker content in related year (t clinker/t cement); $Ef_{clinker}=$Clinker production CO_2_ emission factor in related year (tCO_2_/t clinker).

Two activity data sets are needed in applying the above equation: cement production and clinker content in cement evolution along the estimations timeframe (1970-2015). Cement production (national and subnational) has been obtained through direct communication with the Brazilian National Cement Association (SNIC) and in its annual report 2013 (ref. 90). In this way, cement annual production for period between 1971 and 2013 was compiled. For 1970, 2014 and 2015 national production was obtained by Ministry of Mines and Energy page.

Cement clinker content was obtained through cement and clinker production data published in the MCTI reference report for the period between 1990 and 2010; for years prior to 1990 and subsequent to 2010, the proportion for closest years available were used.

Emission factors were obtained in a similar manner to cement clinker content: for the period 1990-2010 were used CO_2_ emissions and clinker production presented in the MCTI reference report to obtain an implicit emission factor for each year. To fill the remaining gaps in the historical series the procedure was the same adopted for obtaining the clinker content.

#### Lime production

Calcium and magnesium carbonate (MgCO_3_ and CaCO_3_) calcination is part of the lime production process. Both minerals (calcite and dolomite limestone) emit CO_2_ when heated in lime production kilns. The energy sector comprises emissions from fuel combustion in production kilns. Supplementary Figure S8 shows quicklime (CaO and MgO mixture) and hydrated lime (a mixture of Ca(OH)_2_ and Mg(OH)_2_) production process.

SEEG used the methodology published by the Brazilian Ministry of Science, Technology and Innovation for its GHG emissions related to the reference report on mineral products^88^. The emission estimation applies specific emission factors for each lime type chemical composition: calcite, dolomitic and magnesite.

$$E_{i}=Prod_{i}*Ef_{i}$$

Where: $\;E_{i}=$Lime type *i* production annual CO_2_ emissions (tCO_2_/year); $Prod_{i}=$Lime type *i* annual production (t lime/year); $Ef_{i}=$Lime type *i* production CO_2_ emission factor (tCO_2_/t lime).

All quicklime is calcite and hydrate lime is divided into 20% calcite, 30% dolomitic and 50% magnesite. To obtain information on the evolution of national quicklime and hydrated lime production evolution for the period between 1990 and 2012 we used data from the reference report^88^.

Total lime production data between 1970 and 1989 was obtained from the reference report of the geology mining and mineral processing plan on lime profile^91^. Lime production profile (quicklime and hydrated lime) from 1990 was applied to detail these data. Quicklime and hydrated lime production data in 2013 and in 2014 have been obtained through direct communication with the Brazilian Lime Producers Association. During preparation of this document, there was no public information available on lime production for 2015, therefore, lime production was considered constant between 2014 and 2015.

The ABPC lime production dataset also made it possible to allocate emissions between three main federative units regarding this activity (PR, MG and SP) during the period 2005-2015. Each lime type emission factor depends on its chemical composition, which is directly related to limestone and dolomite consumption in the production kiln.

Lime type chemical composition and consequent emission factors used are the same as in MCTI^88^. There were no available data for evolution of lime chemical composition. Therefore, the same composition was applied throughout the SEEG timeframe.

#### Glass production

Glass production kilns consume, among other minerals, limestone and dolomite, which emit CO_2_ due to a calcination reaction that occurs at high temperatures. Emissions accounted for in this section are related only to this consumption. Soda ash consumption emissions are estimated as described in section below (soda ash consumption) and fuel combustion emissions in kilns are estimated in the energy sector. Glass production process and associated emissions can be represented by Supplementary Figure S9.

SEEG uses the same methodology presented by the MCTI^88^. Glass production from raw materials (difference between glass total production amount and recycled glass production amount) is the main driver for emissions as shown by the following equation:

ECO2,i=Prodraw material glass*Xi*EfCO2,i

Where: $\;E_{CO_{2},i}=$Carbonate *i* consumption annual emissions in glass production from raw materials (tCO_2_/year); Prodraw material glass=Annual glass production from raw materials (t glass/year); $X_{i}=$Carbonate *i* specific consumption in glass production from raw materials (t carbonate/t glass); $Ef_{CO_{2},i}=$Carbonate *i* CO_2_ emission factor in glass production from raw materials (t CO_2_/t carbonate).

Reference report contains glass production evolution. Regarding production for period prior to 1990, an exponential function obtained through the available values for the period 1990-2011 was used. The recycled glass percentage provided by the reference report (11%) was applied to obtain glass production from raw materials for 1970-1989 (ref. 88).

Since glass production data for years between 2012 and 2015 was not available in the latest version of the statistical yearbook of the non-metallic transformation sector^92^, emissions in 2011 were repeated for these years. It was not possible to gather subnational level publicly available information regarding glass production.

Specific carbonate consumption applied was the same presented in the reference report: 10% for limestone and 2% for dolomite^88^. Emission factors are the same applied in estimations for carbonate consumption in blast furnace emissions: 0.440 tCO_2_/t limestone and 0.477 tCO_2_/t dolomite.

#### Soda ash consumption

Soda ash (Na_2_CO_3_) consumption is responsible for GHG emissions related to pulp and paper industries, soaps and detergents production, water treatment and glass production. SEEG uses the same methodology presented by the Brazilian Ministry of Science, Technology and Innovation on its GHG emissions related to mineral products reference report^88^. CO_2_ emissions are the product between soda ash consumption in tons and emission factor in terms of tCO_2_/tNa_2_CO_3_. Emission factor based on soda ash consumption reaction stoichiometric ratio is 0.415tCO_2_/tNa_2_CO_3_^88^.

For years between 1990 and 2011 soda ash consumption was obtained in the MCTI reference report, for remaining years, data was obtained in versions of chemical industry yearbook published by the Brazilian Chemical Industry Association (Abiquim)^93^ as listed in Supplementary Table S5. It was not possible to gather subnational level publicly available information regarding soda ash consumption.

#### Emissions estimation methodology: Chemical industry

Chemical industry emissions in IPPU occur when estimated gases are by-products of other chemical production processes. Supplementary Table S6 summarizes chemical substances whose production processes emissions have been estimated and related GHG.

In some processes, CO_2_ emissions are from carbon stored in biomass used as a raw material. SEEG assumes these emissions have been offset by CO_2_ absorption occurring in the photosynthesis process, which generated the biomass. CO_2_ emissions associated with biomass combustion or use are reported in Agriculture and Land Use Change sectors^12^. Regarding other GHG, biomass emissions must also be accounted for.

Emissions were estimated based on two data sets: emission factors and chemical substance production. Following equation represents applied methodology.

$$E_{g,p}=Prod_{p}*Ef_{g,p}$$

Where: $\;E_{g,p}=$Gas *g* annual emissions from chemical substance *p* production (t gas/year); $Prod_{p}=$Chemical substance *p* annual production (t substance/year); $Ef_{g,p}=$Gas *g* emission factor from chemical substance *p* production (t gas/t substance).

The dataset information sources for chemical substance production are the chemical industry yearbooks published by the Abiquim^93^ available editions and the reference report on chemical industry GHG emissions published by the MCTI^94^. Supplementary Table S5 summarizes information sources for production of most chemicals.

For some products, these information sources were not sufficient to fill data needed in SEEG estimations, adjustments made are described in the following sections.

#### Ammonia production

Hydrogen production (an input in the ammonia production process) from natural gas, asphalt residue, refinery gas and naphtha emits CO_2_. It is also possible to obtain hydrogen from ethanol, but in that case, net CO_2_ emissions associated are considered zero as explained above. Although part of this CO_2_ is used as input in urea and methanol production in integrated plants, as refrigerant in liquid carbonation or as inert gas, in all these cases it ends up being released to atmosphere in the short term^94^.

The emission factor used in estimations represents an average of measurements made by each of the producing companies, since emissions are dependent on the raw material consumed in hydrogen production. For the entire SEEG timeframe, the emission factor was 1.46 tCO_2_/t ammonia^94^.

The subnational level emissions driver is federative unit installed capacity evolution obtained in the latest published version of Abiquim^93^.

#### Nitric acid production

Traditional nitric acid (HNO_3_) production is based on ammonia catalytic oxidation with air, followed by product oxidation with water (Ostwald process), these reactions release NO_x_ emissions during processing. Furthermore, ammonia also participates in undesirable side reactions; in one of them, nitrous oxide (N_2_O) is a by-product. Reactions mentioned are shown below:

Ammonia catalytic oxidation reaction sequence and HNO_3_ production:

4 NH_3_ + 5 O_2_ → 4 NO + 6 H_2_O

2 NO + O_2_ → 2 NO_2_ → N_2_O_4_

3 NO_2_ + H_2_O → 2 HNO_3_ + NO

Undesirable side reactions:

4 NH_3_ + 4 O_2_ → 2 N_2_O + 6 H_2_O

4 NH_3_ + 3 O_2_ → 2 N_2_ + 6 H_2_O

2 NO →N_2_ + O_2_

4 NH_3_ + 6 NO → 5 N_2_ + 6 H_2_O

Some Brazilian nitric acid plants use high-pressure conditions during the process; this technological route does not emit NO_x_ and N_2_O. Thus, production used in estimations should represent only the amount related to emitting plants. For the period between 1990 and 2010, the MCTI reference report was the information source for this dataset. For periods prior to 1990 and subsequent to 2010, SEEG methodology applied the closest available years proportions between nitric acid emitter production and total nitric acid production (70% in 2010 and 75% in 1990).

Subnational level emissions driver is federative units installed capacity evolution obtained in latest published version of Abiquim^93^.

N_2_O emissions from plants that do not use high-pressure technological route, actual emission actual measurements were made through emission control projects or the default emission factor shown in IPCC 2006 Guidelines by Tier 1 method^12, 94^. Since this reference report does not present the emission factors, an implicit emission factor was calculated through nitric acid production by emitting plants and emissions reported in the reference report for period between 1990 and 2010. Prior to 1990, emissions were estimated by factor calculated up to 1990 (6.12 kgN_2_O/tHNO_3_), subsequent to 2010, by a factor calculated for 2010 (2.22 kgN_2_O/tHNO_3_). GHG emissions control projects occurring after 2010 were not considered and will be incorporated in SEEG’s future versions.

NO_x_ emissions were estimated by the factor shown in the MCTI^94^, specific to national production conditions, which consider GHG emissions control in the country: 1.75 kgNO_x_/tHNO_3_.

#### Adipic acid production

There is only one plant in Brazil responsible for all national adipic acid production. It performs a two-stage process: (i) cyclohexane oxidation to produce a mixture cyclohexanone/cyclohexanol, (ii) cyclohexanol oxidation by nitric acid; step (ii) emits nitrous oxide (N_2_O). An installation for thermal decomposition of N_2_O into N_2_ was built through an emissions control project, dramatically reducing GHG emissions from 2007.

N_2_O emissions were estimated using emission factors obtained during the emission control project: 0.27 tN_2_O/t adipic acid for the period from 1990 to 2006 and ranging between 0.0064 and 0.00155 tN_2_O/t adipic acid for the period 2007-2010 (ref. 94). The 2010 factor has been set for the later years and the 1990 factor has been set for prior years.

CO and NO_x_ emission factors take into account GHG emission control in the Brazilian plant. As published in MCTI reference report, these factors are 16 kgCO and 5 kgNO_x_/t adipic acid. This substance production has same information sources presented in Supplementary Table S5, except for the period between 2011 and 2015, for which no data were available and 2010 production was repeated for simplification. All emissions were allocated in São Paulo (federative unit where the plant is installed).

#### Caprolactam production

The only caprolactam production plant closed down in 2010. This production was performed by benzene hydrogenation to cyclohexane, followed by oxidation of this compound to cyclohexanone and cyclohexanol by HNO_3_ (N_2_O emissions occur at this stage); finally, cyclohexanol is dehydrogenated and reacts with sulfate.

An emission factor from 2006 IPCC Guidelines Tier 3 methodology^12^ (direct measurements) was applied for the entire temporal scope: 6 kgN_2_O/t caprolactam^94^. This factor has been used in SEEG estimations. All emissions were allocated in Bahia (federative unit where the plant was installed).

#### Calcium carbide production

Calcium carbide national production is carried out by reducing quicklime (CaO) with petroleum coke or charcoal (C). Emissions related to lime production (limestone calcination) are counted in the Mineral Products subsector. Lime reduction through petroleum coke emissions should be allocated as a chemical industry process. These emissions are associated with the following chemical reactions:

CaO+3C→CaC_2_+CO(+½O_2_→CO_2_)

Neither calcium carbide production data nor the associated emission factors are published by the only manufacturer in Brazil, which classifies them as confidential in the MCTI reference report. Thus, for period between 1990 and 2010, emissions reported by SEEG are the same presented by MCTI reference report. 2010 emissions were repeated for the period 2011-2015 for simplification. Emissions began to take place in 1995, previous emissions were considered zero^94^. It was not possible to gather subnational level publicly available information regarding calcium carbide production as well.

#### Methanol production

Methanol production in Brazil is performed by high and low pressures synthesis, using natural gas as raw material (methane is the major component in this fuel mixture) and carbon dioxide. The main GHG emissions resulting from this process are the raw materials themselves (CH_4_ and CO_2_).

Emission factors applied for the entire period are those presented by MCTI^94^: 0.267 tCO_2_ and 2,3 kgCH_4_/t methanol. It was not possible to gather subnational level publicly available information regarding methanol production.

#### Ethylene production

Typically, petrochemical substance cracking is an ethylene production process. This production route also generates propylene, butadiene and aromatics. In Brazil, naphtha is used, in general, in cracking reaction; in 2004 national production started to use natural gas started as another source through pyrolysis process. This route emits CO_2_, CH_4_ and NMVOC.

CO_2_ and CH_4_ emission factors used are the same presented by MCTI reference report. According to it, they are Tier 1 factors^12^, with appropriate corrections to existing steam cracking process in South America; furthermore, they contemplate temporal variations in methane emission factor.

Until 2005, factors used were 1.73 kgCO_2_/t ethylene and 3 kgCH_4_/t ethylene. Since 2006, CO_2_ factor became 1.74 kgCO_2_/t ethylene. Methane factor became 3.54 kgCH_4_/t ethylene for 2006-2009 and 3.25 kgCH_4_/t ethylene for subsequent years. Non-methane volatile organic compounds (NMVOCs) emission factors are IPCC’s 1996 Guidelines 1.4 kgNMVOC/t ethylene^94^. It was not possible to gather subnational level publicly available information regarding ethylene production.

#### Dichloride ethylene and vinyl chloride production

Dichloride ethylene and vinyl chloride (VCM - vinyl chloride monomer) production plants can operate through a balanced process between two products, by the direct chlorination and ethylene oxychlorination technological route. Since ethylene conversion is not 100% and the unreacted fraction is converted to CO_2_ before being emitted to the atmosphere in order to meet environmental control requirements, it generates NMVOC, CO_2_ and CH_4_ emissions. It was not possible to gather subnational level publicly available information regarding dichloride ethylene and VCM production.

CH_4_ and CO_2_ emission factors applied are based on vinyl chloride production through IPCC 2006 Guidelines Tier 1 methodology^12^: 0.294 tCO_2_/tVCM and 0.0226 kgCH_4_/tVCM^94^. NMVOC emissions are estimated separately for each product according to MCTI reference report: 8.5 kgNMVOC/tVCM and 2.2 kgNMVOC/t dichloride ethylene.

#### Ethylene oxide production

In Brazil, ethylene oxide is produced by ethylene direct oxidation with air. In this process, there are carbon dioxide and methane emissions. Production before 2011 was obtained as shown in Supplementary Table S2. From 2011 on, this substance production was not disclosed in Abiquim yearbooks. Thus, 2010 production was kept constant for the 2011-2015 period. It was not possible to gather subnational level publicly available information regarding ethylene oxide production.

Carbon dioxide emission factor was estimated by the 2006 IPCC Guidelines Tier 2 method (carbon mass balance in production process); methane emissions already used the default factor^12^. The following values were applied in SEEG methodology: 0.52 tCO_2_/t ethylene oxide and 1.79 kgCH_4_/t ethylene oxide.

#### Acrylonitrile production

Acrylonitrile production process uses Sohio catalytic reaction technology between propylene, ammonia and air. This process also produces acetonitrile and hydrocyanic acid (by-products). This propylene ammoniation does not have a 100% yield in acrylonitrile production. Thus, a portion is converted to carbon dioxide or to other hydrocarbons (methane and NMVOC) by direct oxidation.

Information sources for acrylonitrile production are the same presented before regarding ethylene oxide: 2011-2015 emissions were estimated applying 2010 production due to data unavailability. All emissions were allocated in Bahia (federative unit where the plant is installed).

CO_2_ and methane emission factors are also very similar to the ones applied for ethylene oxide (Tier 1 and Tier 2, respectively). NMVOC emissions were estimated using Tier 1 factors presented in the 1996 IPCC Guidelines^12^. Values used in SEEG estimations are 0.2325 tCO_2_/t acrylonitrile; 0.18 kgCH_4_/t acrylonitrile and 1 kgNMVOC/t acrylonitrile.

#### Calcined petroleum coke production

Alumina electrolysis for metallic aluminum production uses calcined petroleum coke as an input. This calcined coke is produced from petroleum green coke anode grade through a thermal process that reduces volatile matter content in the original coke. This volatile matter consists mainly of methane, which is emitted to the atmosphere (Petroleum green coke anode grade → calcination → Calcined petroleum coke + CH_4_).

Production data was obtained as shown in Supplementary Table S5 for period 1990-2015. 1990 production was kept constant for period 1985-1989, for simplification caused by data unavailability. All emissions were allocated in São Paulo (federative unit where the plant is installed). Emission factor information source is IPCC 1996 Guidelines: 0.5 kgCH_4_/t coke^14^.

#### Carbon black production

Aromatic residues and heavy fuel oil (hydrocarbon sources) partial oxidation is the production route for carbon black in Brazil. This process produces a purge gas and its consumption generates CO_2_, CH_4_ and NO_x_ emissions.

CO_2_ emission factors were estimated by Tier 2 method, CH_4_ through Tier 1 and NO_x_ through specific emission factor by Abiquim: 1.618 tCO_2_/t carbon black, 0.06 kgCH4/t carbon black and 0.14 tNO_x_/kg carbon black^94^.

Production data survey follows Supplementary Table S2 for the period until 2010; from 2011, 2010 production was fixed by simplification due to data unavailability. It was not possible to gather subnational level publicly available information regarding carbon black production.

#### Phosphoric acid production

Phosphoric acid production is made by reacting phosphate rock and sulfuric acid. This reaction makes calcium carbonate in the rock react with acid-forming CO_2_ and plaster. CO_2_ emission factors were developed based on national phosphate rocks chemical composition and are presented in MCTI^94^ as 0.02 kgCO_2_/t phosphate rock.

Production data was obtained in MCTI reference report for period between 1990 and 2012. As a simplification, 2012 production was kept constant for 2013-2015. For years prior to 1990, it was estimated by the trend of production temporal evolution in the period 1990-2005, as a simplified way of estimating this variable in the absence of more accurate information for the period. It was not possible to gather subnational level publicly available information regarding phosphoric acid production.

#### Other chemical products production

A significant number of other products had their NMVOC emissions production estimated using emission factors presented in the IPCC 1996 Guidelines^14^: ABS resins, phthalic anhydride, styrene-butadiene rubber (SBR), styrene, ethylbenzene, formaldehyde, polyvinyl chloride (PVC), polystyrene, polyethylene (HDPE, LDPE, LLDPE), polypropylene and propylene.

MCTI^94^ also presents the European emission inventory as an information source for emission factors (phthalic anhydride, polyvinyl chloride and polystyrene) and Abiquim^93^ (styrene-butadiene rubber - SBR).

These product production information sources are the same presented in Supplementary Table S2. The following exceptions used simplifications in the absence of data (linear regressions or production fixed for the period known):

- ABS Resins: production between 1985 and 1989 estimated by linear interpolation of 1984 and 1990 values, production in 2011 kept constant for the period between 2012 and 2015;
- Phthalic anhydride, butadiene styrene and ethylbenzene: production in 2011 kept constant for the period between 2012 and 2015;
- Polyethylene disaggregated by type: for years 1970, 1971, and 2012-2015 polyethylene total production was the only available data. Type breakdown was carried out by the proportions in closest available years (1972 and 2011 respectively).

It was not possible to gather subnational level publicly available information regarding production of these substances.

#### Emissions estimation methodology: HFC emissions

Some halogenated hydrocarbons or halocarbons are used as refrigerant fluids in refrigeration equipment or as gas in aerosols. These are compounds containing chlorine (Cl) and fluorine (F). Hydrofluorocarbons (HFCs) began to be used as replacement for chlorofluorocarbons (CFCs) and hydrochlorofluorocarbons (HCFCs), after the restriction use on the latter ones by the Montreal Protocol in 1987. HFCs emissions occur during halocarbons production process and during assembly, use or disposal of products containing HFCs.

#### Halocarbons production

There is no HFCs production in Brazil, but HCFC-22 production has HFC-23 as a by-product. As published by the MCTI on HFCs and SF_6_ production and consumption related GHG emissions, the HFC-23 emission factor in terms of HCFC-22 production is 0.04 tHFC-23/tHCFC-22 (ref. 95).

HCFC-22 production between 1990 and 1999 was obtained in the MCTI^95^. From 2000, this gas was no longer produced in Brazil and it was not possible to obtain reliable data about its production for periods prior to 1990. Also, it was not possible to gather subnational level publicly available information regarding HCFC-22 production.

#### Halocarbons consumption

The MCTI reference report on HFCs and SF_6_ production and consumption-related GHG emissions estimates emissions from halocarbon consumption, i.e. emissions that occur during the assembly, operation (use) steps of products containing HFCs through Tier 1b methodology^12^. This methodology estimates emissions from a "potential emissions" approach, considering "bulk" gases import and export flows and gases contained in imported and exported equipment flows.

SEEG reported emissions of HFC-32, HFC-125, HFC-134a, HFC-143a and HFC-152a for the period between 1990 and 2010 are the same as the potential emissions presented by the MCTI^95^. For years prior to 1990, it was assumed that consumption of these gases was not relevant; thus, the emissions reported for these years are zero. In the absence of HFC import information for period between 2011 and 2015, SEEG methodology was the same presented by the MCTI^10^: emissions related to this period were estimated from extrapolation of linear functions as shown in the following equations, in which *X* indicates the estimation year emissions.

$$E_{\mathrm{HFC}-32}^{X}=X*14.75-29,549$$

$$E_{\mathrm{HFC}-125}^{X}=X*46.31-92,656$$

$$E_{\mathrm{HFC}-134a}^{X}=X*412.55-823,371$$

$$E_{\mathrm{HFC}-143a}^{X}=X*43.564-87.174$$

In particular, SEEG reported emissions are zero for HFC-152a during 2007-2015, as MCTI reference report^95^ shows 2006 as the last year in which they occurred. It was not possible to gather subnational level publicly available information regarding HFC consumption.

#### Emissions estimation methodology: SF6 in electrical equipment

Besides fused magnesium protection application, sulphur hexafluoride (SF_6_) is a gas used as an insulator in electrical equipment (switches and large breakers) and emissions of this gas are due to losses during maintenance and disposal of such equipment.

Emissions reported by SEEG for the period between 1990 and 2005 are the same as published by the MCTI^9^. According to this inventory, these emissions were estimated using a default emission factor for equipment and installed capacity was obtained through a survey coordinated by the Brazilian Electricity Regulatory Agency and MCTI^9^.

For the period between 2006 and 2010, emissions were the same presented by the MCTI^10^. According to the methodology used in this report, emissions for the period between 2011 and 2015 were estimated through linear regression of emissions between 2001 and 2005.

In the absence of more detailed information about the SF_6_ installed capacity in electrical equipment, reported emissions between 1970 and 1989 are zero. It was not possible to gather subnational level publicly available information regarding SF_6_ consumption in electrical equipment.

#### Emissions estimation methodology: Non-energy products from fuel and solvent use

The Brazilian National Energy Balance published by MME presents fuel non-energy use data, designating them as "Non-Energy Final Consumption". In detailed worksheets (Matrix 49×47), this data is further broken down into two categories: (i) raw material for the chemical industry and (ii) other uses. Emissions associated with fuel non-energy consumption as raw material in the chemical industry have been accounted for and described in the “Chemical industry" category^94^. Consumption associated with "Other uses" is responsible for two emissions-generating activities^94^:

- Lubricant energy consumption CO_2_ emissions in two-stroke engines: estimated considering that 20% of the final non-energy consumption lubricant flow corresponds to that end and through methodology and emission, factors presented by the Brazilian Ministry of Science, Technology and Innovation in its fuel combustion GHG emissions – Bottom-Up approach reference report^80^.
- Anhydrous and hydrous ethanol, other non-energy oil products and solvents non-energy consumption NMVOC emissions: it is considered that all consumption of these energy sources is transformed into non-methane volatile organic compounds; thus, the volume flows were only converted to mass using density from MME’s BEN^65^.

Subnational level emissions from these activities were not allocated for data unavailability.

### Calculation of the GHG emissions: Waste sector

The SEEG project estimated CH_4_ emissions from solid waste disposal, CO_2_ and N_2_O emissions from incineration and open burning of waste and CH_4_ and N_2_O emissions from wastewater treatment and discharge. In general, the methodology adopted by SEEG initiative follows the methodology MCTI^10^, which in turn is based on the IPCC guidelines^12, 96^.

The analysis of the waste sector is characterized by the low availability of reliable data from public or open sources. The activity data were obtained directly from open sources, such as the annual panoramas of the *Brazilian Association of Public Cleaning and Special Waste Companies (ABRELPE)98* or relevant studies at national level. In specific cases, where data were not available, we used information detailed in the waste sector reference report of the BIs^97^ and mathematical correlations.

The methodology uses Tier 1 and 2 emission factors provided in the reference report of the MCTI^97^ and in the IPCC^12, 96^. Overall, the sector is characterized by the use of default values for the emissions factors and the incorporation of a country specific activity data. As for the software utilized, all database management and estimates were entirely performed using the Microsoft Excel tool.

#### Methane emissions from solid waste disposal sites (SWDS)

The methodology adopted in the BIs^97^ was not replicable due to limitations in the data available. The SEEG approach for estimating CH_4_ emissions is based on the Methane Commitment method, presented in the following equation, which assumes that all the degradable organic content (DOC) in a specific year will decay and produce methane immediately^96^.

$$CH_{4}emissions=\Sigma [MSW_{t}\times MSW_{f}\times L_{0}\times \left(1-f_{rec}\right)\times \left(1-OX\right)]$$

Where: *CH*_*4*_ *emissions*: CH_4_ emitted in year T (tons); *MSW*_*t*_: Amount of waste disposed (tons); *MSW*_*f*_: Fraction of MSW disposed at SWDS; *L*_*O*_: Methane generation potential (CH_4_ tons/tons of MSW); *F*_*rec*_: Fraction of methane recovered at the landfill (flared or energy recovery); *OX:* Oxidation factor

### Activity data

Total municipal solid waste (MSW_t_) and the fraction of MSW sent to SWDS (MSW_f_)

The amount of waste disposed was calculated by multiplying the urban population by the collection rate per capita of each Brazilian region. These values were obtained via the Brazilian Institute of Geography and Statistics (IBGE); for historical data on collections rate we used the national inventory and mathematical correlations. Finally, for recent years data from ABRELPE^98^ were used.

The historical assessment of the Brazilian solid waste management observed in the period of 1970-2015 allowed us to obtain the fraction of MSW sent to SWDS and the management practice at these disposals sites. Brazil has introduced sanitary landfill practices, more significantly, in the 1990s (ref. 99). In 1991, only about 4.3% of the MWS was properly disposed of at managed landfills^99^.

Currently, about 58.7% of the MSW collected is sent to sanitary landfills, 24.1% to unmanaged (≥5 m deep) and 17.2% to dumps^98^. Other waste management practices, such as composting and anaerobic digestion are still negligible at national scale. Furthermore, it is important to remark that this panorama presents high regional discrepancies, where the Southeast region of Brazil presents the highest access to basic services, in contrast with the North region that presents the lowest indices (Supplementary Figure S10). This characteristic has been incorporated at the SEEG estimate.

#### Fraction of methane recovered (Frec)

The fraction of methane recovered for each state was obtained, for the period of 2003-2010, through the reference report and extrapolated for the subsequent years. According to MCTI^97^, before 2003, measures of recovering and burning CH_4_ in a flare or an energy device were not significant in Brazil.

#### Oxidation factor (OX)

The OX reflects the quantity of methane that is oxidized in the soil or other material covering of the SWDS^12^. Well-managed sites tend to have a higher oxidation rate than unmanaged dump sites. For unmanaged landfills, the SEEG project has utilized the default value of zero and for managed landfills, the oxidation value of 0.1 has been applied.

#### Methane generation potential (L_0_)

The basis for the calculation of the methane generation potential is defined in the following equation.

$$L_{0}=MCF\times DOC\times DOC_{f}\times F\times \frac{16}{12}$$

Where: *L*_*0*_: CH_4_ generation potential (tons CH_4_/tons waste); *MCF:* CH_4_ correction factor (fraction); *DOC*: Degradable organic carbon in the year of deposition (tons C/tons MSW); *DOC*_*f*_: Fraction of DOC that can be decomposed (fraction) - Based on the recommendations of the IPCC^12^ and MCTI^10^ the fraction of degradable organic carbon which decomposes (DOC_f_) default value is 0.5.; *F*: Fraction of CH_4_ in generated landfill gas (volume fraction)-The composition of the gas generated in SWDS is approximately 50 percent methane. The default value used was 0.5 (ref. 5–27).

#### MCF (methane correction factor)

The MCF refers to the fraction of waste degraded anaerobically, this factor takes into account the fact that unmanaged SWDS produce less CH_4_ than anaerobic managed sites. According to the IPCC^12^, the MCF for each category is:

- Managed=1.0
- Unmanaged (≥ 5 m deep)=0.8
- Unmanaged (< 5 m deep)=0.4

DOC (degradable organic content)

The degradable organic content (DOC) was calculated using a linear regression provide in the reference report of the latest Brazilian inventory. It considered various combinations of gravimetric composition of the waste generated in different cities around Brazil, covering the period of 1970-2010. As result, the following equation and coefficients described in Supplementary Table S7 were obtained. In our study, the function presented was extrapolated to subsequent years.

DOC(t)=a×t+b

Where: DOC (t): degradable organic content; a: angular coefficient; b: linear coefficient; *t:* inventory year

#### DOC_f_ (degradable organic content)

The value of the fraction of DOC that can be decomposed (*DOC*_*f*_) used in SEEG is the default value 0.5 (50%), which is based on the recommendations of the IPCC^12^ and MCTI^10^.

#### F (Fraction of CH_4_ in generated landfill gas)

The value of fraction of CH_4_ in generated landfill gas (F) (volume fraction) used in SEEG is the default value 0.5 (50%), which is based on the recommendations of the IPCC^12^ and MCTI^10^.

To estimate the contribution of distinct types of management practices at SWDS, the SEEG project estimated three sequences of L_0_ for the period of 1970-2015. The following Supplementary figure S10 indicates the outcome obtained by the initiative, where each Brazilian region has a distinct methane generation potential, associated with its unique context and waste composition characteristic.

#### Carbon dioxide and nitrous oxide emissions from incineration

Incineration is a technological methodology that is not largely applied in Brazil; is it used mainly for the treatment of Clinical Waste (CW) and Hazardous Waste (HW)^97^. This process may generate emissions of CO_2_, CH_4_, N_2_O and other traditional air pollutants from combustion. The third national communication only estimated emissions of carbon and nitrogen dioxides, according to the following equations:

$$CO_{2}\mathrm{emissions}=\Sigma_{i}(IW_{i}\times CCW_{i}\times FCF_{i}\times EF_{i}\times \frac{44}{12})$$

Where: *CO*_*2*_ *emission*: CO_2_ emissions in inventory year (tons/yr); *IW*_i_: Amount of waste incinerated (tons/yr); *CCWi*: Fraction on total carbon content (fraction); *FCF*_i_: Fraction of fossil carbon in the total carbon (fraction); *EF*_*i*_: Burn-out efficiency of combustion of incinerators (fraction); *i*: Type of waste incinerated (CW or HW)

N2O emissions=Σi(IWi×EFi)×10−3

Where: *N*_*2*_*O emission*: N_2_O emissions in inventory year (tones/yr); *IW*_i_: Amount of waste incinerated (tons/yr); *EF*_*i*_: N_2_O emission factor for (kg N_2_O/ tons of waste); *i:* Type of waste (CW or HW).

### Activity data

The major limitation associated with the incineration subsector was the acquisition of activity data on the amount of waste incinerated, either in the historical context or for recent years. For HW, we could not obtain consistent information from sources that were public or free of charge. Given the scenario presented, in the SEEG project we used data provided in the reference report. The state allocation of hazardous material routed to incineration treatment, not described the MCTI^97^, was estimated from the Diagnosis of Industrial Waste, drafted by the Institute for Applied Economic Research^100^.

In relation to CW, it was possible to obtain a more robust literature, mainly as of 2010, with the annual panoramas elaborated by ABRELPE^98^,^101–105^. However, the data presented by the national association of residues and the one showed in the reference report were incoherent. We opted to use data from the first source and applied mathematical correlations to fill the gaps^106^.

#### Emission factor

The methodology uses Tier 1 emissions factors, as well as default values for the fraction of total carbon content, fraction of fossil content and efficiency of the incinerator provided in the IPCC^12^. The estimation is in conformity within the Tier 1 method and considering the low representative of the subsector, it is therefore a good practice.

#### Methane and nitrous oxide emissions from wastewater treatment/pathways

Wastewater will produce CH_4_ if it degrades anaerobically, during which the methane production depends on the temperature, type of treatment and primarily on the amount of degradable organic matter in the wastewater. This attribute is usually represented by parameters such as Biochemical Oxygen Demand (BOD) for domestic and Chemical Oxygen Demand (COD) for industrial wastewater, whilst the production of N_2_O is associated with the degradation of nitrogen components in the wastewater^12^.

#### Domestic Wastewater

The methodology used for estimating the emissions of domestic wastewater in the third national inventory was developed by IPCC and can be defined according to the following equation.

$$CH_{4}\mathrm{emissions}=[Pop\times D_{dom}\times B_{0}\times \sum_{x}(WS_{i,x}\times MCF_{x})]-R$$

Where: *CH*_*4*_ *emissions*: methane emissions in inventory year; *Pop:* Population; *D*_*dom:*_ Degradable organic component (kg BOD/persons/yr); *B*_*0*_: Maximum methane producing capacity (kg Ch4/kg BOD or Kg Ch4/kg COD); *WS*_*i,x*_ *x MCF*_*x*_: weighted methane correction factor (fraction); *WS*_*i,x*_ *x MCF*_*x*_: weighted methane correction factor (fraction); *R*: amount of CH_4_ recovered in inventory year (kg CH_4_/yr).

In the SEEG project it was not possible to replicate the methodology for estimating emissions of CH_4_ applied by the MCTI due to the limitation on data available. The major constraining factor was the absence of data regarding wastewater treatment systems and discharge pathways adopted by different municipalities in Brazil, whether in the historic context or even for recent years.

Default values to quantify maximum methane production capacity (B_0_) and national data to establish an average per capita degradable organic component (D_dom_) were used in the BIs. The MCTI assessment also showed fractions of effluents treated for specific types of technology (WSi, x), as well as the default values of the methane conversion factors (MCF) for different treatment systems commonly adopted in Brazil^97^. However, the calculation of emissions from treatment of domestic effluents is marked by the lack of consolidated information at state level.

For the SEEG project, we estimated the emission per capita obtained in the BIs for different years of the inventoried period and apply this rate to the population with sewer systems^97^ of each state. This calculation enabled the attainment of CH_4_ emissions values in a statewide perspective.

Although the MCTI estimates the emissions based on the methodology proposed by the IPCC^12^, the lack of information in the reference report generates a method that is difficult to replicate. The methodology used by the SEEG initiative to quantify emissions of methane, is marked by the low quality and a strong need for improvement.

The N_2_O emissions of domestic liquid effluents were quantified from the replication of the methodology applied in the BIs^97^, described in the following equation.

N2O emissions=PopxCPxFracNPRxEFefluentx4428

Where: *N*_*2*_*O emissions:* N_2_O emissions in inventory year (kg N_2_O/yr); *Pop:* Total population with sewer system (hab); *CP:* Annual per capita protein consumption (kg/person/yr); *Frac*_*NPR*_: Fraction of nitrogen in protein (fraction); *EF*_*efluent*_: Emission factor for N_2_O emissions from discharged to wastewater in kg N_2_O-N into kg N_2_O.

#### Activity data

The population considered in this analysis, as well as the one used for estimate CH_4_ emissions, is composed by the fraction of households with sewage. The IBGE data were described in the BIs^97^, while the information gaps were filled from mathematical interpolation and extrapolation relations. For the annual per capita protein consumption, the values were based on Tier 2 data, sourced from the reference report of the waste sector and interpolated for gap years.

#### Emission factor

The emission factor and fraction of nitrogen in protein were indicated as Tier 1 default values in the IPCC^12^ and reproduced by the SEEG initiative.

#### Methane emissions from industrial wastewater

To quantify emissions associated with treatment and discharge systems of industrial effluents for the period of 1970-2015, the SEEG initiative reproduced the methodology proposed by the MCTI, which in turn, is based on the methodology developed by the IPCC^12, 96^. The nine strategic industrial sectors selected in the national communications are described below, as the sum of their contributions represents more than 97% of the organic load generated in Brazil^97^: (i) Sugar Production; (ii) Alcohol Production; iii) Beer production, (iv) Meat Production (bovine, swine and poultry), (v) Milk Production (raw and pasteurized) and vi) Pulp and Paper production

$$CH_{4}emissions=\left[P_{i}\times D_{ind}\times B_{0}\times \Sigma \left(WS_{i,x}\times MCF_{x}\right)\right]-R$$

Where: *CH*_*4*_ *emissions:* CH_4_ emissions in inventory year (kg CH_4_/yr); *i:* industrial sector ; *P*_*i*_: total industrial product for industrial sector i (tons/yr); *D*_*ind*_: BOD per ton of industrial production [kg BOD](tons production)^−1^ ; *B*_*0*_: maximum CH_4_ producing capacity (kg CH_4_/kg BOD); *WS*_*i,x*_ *x MCF*_*x*_: weighted methane correction factor (fraction); *R*: amount of CH_4_ recovered in inventory year (kg CH_4_/yr).

#### Activity data

The production data for the analyzed industrial sectors were obtained from different sources, specified below:

- Sugar: Sugarcane Industry Association^64^
- Alcohol: Sugarcane Industry Association^64^
- Raw milk: Municipal Livestock Research (IBGE/SIDRA)^29^
- Pulp and Paper: data from the Brazilian Tree Industry (IBÁ)^75^.
- Beer: Data from 2006 to 2011 are from the Annual Industrial Survey available in the database of the IBGE/SIDRA^29^. The data for 2012-2014 are from the Beverage Production Control System (SICOB). Data on the national production of 2015 were provided by Brazilian Beer Industry Association^107^.
- Cattle Slaughter: IBGE Quarterly Survey of Animal Slaughter^29^
- Poultry Slaughter: IBGE Quarterly Survey of Animal Slaughter^29^
- Swine Slaughter: IBGE Quarterly Survey of Animal Slaughter^29^
- Pasteurized milk: the official data of the IBGE^29^ refer to the period from 1989 to 1996. Due to the non-location of other production data and the lack of linear growth for the allocation of production to each State, we used correlations between population and official data^29^.

The analysis of technologies for treatment of industrial effluents, percentages of treatment and launching in water bodies carried out by MCTI^97^ for each sector, made it possible to obtain the fraction of industrial effluent treated anaerobically and the MCF weighted for each activity for 2010. With defaults values proposed by the IPCC^12^ and mathematical correlations of interpolation, the weighted MCF values were obtained for the period of 1990-2010 and extrapolated to 1970-1989 and 2011-2015.

#### Emission Factor

The emission factor of organic load per unit produced is available in the reference report, for each industrial sector, as well as data for the maximum CH_4_ producing capacity, default from the IPCC^96^, and information on the methane recuperated for the period of 1991-2010.

### Calculation of GHG emissions: Land use change and forest sector

#### Carbon dioxide emissions from Land use change and forest sector

The method for estimating emissions due to land and forest use change for SEEG used the same methodology used by the MCTI^107^. That methodology was adopted due to the lack of data on land cover with sufficient characteristics for reproducing the approach of the MCTI^10^, which covered the period from 1994-2002 for all biomes, 2002 and 2010 all biomes except Amazon biome, and 2002-2005 and 2005-2010. The methodology adopted implies that the annual emissions were calculated using the following equation as example:

$$E_{CO2e}\left(b,t\right)=\frac{D(b,t)}{\bar{D}(b,\left[1994,2002\right])}x{\bar{E}}_{CO2e}\left(b,\left[1994,2002\right]\right),t=2006,\ldots 2012$$

Where: $E_{CO2e}\left(b,t\right)$: raw emissions of CO_2_ equivalent in year t, t=2006, ..., 2012; D(b,t): area deforested in year t, t=2006, ..., 2012.; $\bar{D}(b,\left[1994,2002\right])$: annual deforestation rate in the period from 1994 to 2002; ${\bar{E}}_{CO2e}\left(b,\left[1994,2002\right]\right)$: average annual raw emissions of CO_2_ equivalent in the period from 1994 to 2002.

That equation assumes that the emissions are proportional to the area annually deforested. It was verified that during the period from 1994 to 2002, there was a high correlation between the total area deforested and the total emissions of CO_2_ equivalent (Supplementary Figure S11). That result showed good compatibility with the results of the inventory^107^. This methodology^107^ used for calculating the emissions consisted of the following steps using as example the period 1994-2002:

1. Separation of raw emissions from net emissions from the 3rd Brazilian Inventory for the period of 1994 to 2002. The tables from the Inventories that contain the net emissions of each one of the Brazilian biomes were used as reference. To calculate the raw emissions of each biome, we considered the sum of positive values. For example, for the Amazon biome, the sum of raw emissions from 1994 (deforestation measured from August 1993 to July 1994) and 2002 (deforestation measured from August 2001 to July 2002) was 8,465,225,000 tCO2, with an annual average of 1,058,153,250 tCO2 (Supplementary Table S8).
2. Calculation of the average annual deforestation based on the National Inventory data^10^. In this step we used the tables that contained the area of change for each inventory period. For each inventory period, we calculated the average deforestation per year. For example, for the Amazon biome the average annual deforestation from 1994-2002 was 19,141 km^2^ (Supplementary Table S9).
3. Calculate the proportional deforestation. The proportional deforestation was calculated by dividing the annual deforestation from a secondary source by the average annual deforestation estimated based on the National Inventory data^10^. For example, in the case of the Amazon biome, the annual data from PRODES^108^ between 1994-2002 was divided by the average annual deforestation calculated based on the Inventory. Supplementary Table S10 presents the years and the source of deforestation data that were used by SEEG for the Brazilian biomes, and we have also included an analysis of the quality of the data available (scale of 1 to 3). Supplementary Table S11 shows the proportion calculated for the Amazon between 1994-2002 as an example.
4. Calculate the gross carbon emissions. The gross carbon emission was calculated by multiplying the proportion calculated between the annual deforestation from secondary sources and the average annual deforestation from the Inventory, by the average gross emission from the Inventory. For example, for the Amazon the average annual gross emission was 1,058,153,250 tCO2. Supplementary Table S12 has an example calculation based on the 1994-2002 Amazon’s data.

#### Methane and nitrous oxide emissions from burning of forest residues

Emissions of methane (CH_4_) and nitrous oxide (N_2_O) are associated with the carbon emitted by burning firewood and extracted timber, as well as the charcoal produced^107^. Data from BEN^65^ for carbon from firewood were used to estimate emissions of those gases. The following equation was utilized:

$$CO_{2residues}=CxEF_{residues}$$

Where: CO_2_ residues=CO_2_ emissions from burning of residues (tCO_2e_); C: Carbon stock (tC); EF residues: emission factor associated with burning of residues.

#### Carbon dioxide removals in conservation units and indigenous lands

Removals of emissions for the land use change sector are calculated using the protected areas (conservation units and indigenous lands), considered to be areas of managed forests^107^. The private natural heritage reserves are not in the calculation. Removals in the UCs and TIs are estimated as an average of 0.62 tCOe ha^−1^y^−1^. For the calculation the conservation units and indigenous lands created up to 1990 and annually from 1990 to 2014 were considered, information that is available in the georeferenced digital file base (shapefiles) of the National Indian Foundation^109^ and the Chico Mendes Institute for Biodiversity Conservation^110^.

#### Carbon dioxide emissions from soil liming

Emissions of carbon dioxide from liming were calculated using the following equation:

$$ECO_{2e}=M_{lime}x0.44$$

Where: $M_{lime}=$quantity of lime applied in agricultural soils (Mt) and 0.44 is the proportion of CO_2_ in pure lime (kg CO_2_/kg CaCO_3_)^111^.

The data from lime were generated by the Brazilian Association of Lime Producers^112^ and the emission factor of 0.44 tCO_2_/tCaCO_3_ was used to estimate emissions of carbon dioxide from liming^12^.

#### Activity data

All of the data available are spatially explicit (Tier 3, or Approach 3, of the IPCC). However, except for the Amazon biome, there are no data for the entire period for the other biomes. In those cases, we used data from the last year available for estimating emissions for the other years.

#### Emission factors

The emissions factors for the land use change sector are represented by increments or average losses of carbon stocks per hectare of area undergoing change. We utilized data from the reference report^9^ to obtain emissions factors for the transition of forest due to deforestation for each biome. The average emissions factors were calculated based on all of the raw emissions generated in MCTI^9^ (Table 4), according to the procedure described above.

The emissions from the other land use transitions were assumed to be proportional to the emissions from transition of forest due to deforestation. That procedure also implies the assumptions that the current average biomass stocks in current deforestation areas have been invariable over time, and that the contributions related to each transition towards total emissions have remained constant. Those assumptions can the avoided with the use of detailed land use change data, when these are available.

#### Means for receiving data

The spatially explicit activity-level data were obtained directly from IBAMA^112^ and PRODES^108^ and are available for free access in a shapefile format (.shp). Additionally, we used deforestation data published by the SOS Atlantic Forest Foundation^113^.

#### Data sequencing treatment

The deforestation data were organized into a geographical information system environment (GIS) for calculating the area. For each biome the projection system indicated by IBAMA^112^ was utilized.

#### Software utilized

ArcGis software was used to obtain summaries of the total area deforested for each biome. Microsoft Excel was used to tabulate and calculate the total emissions. Additionally, Microsoft Access was used to develop a data bank for reproducing the methodology used in the inventory, which may be used to improve the estimations when more data become available.

## Data Records

**Figure 2:**
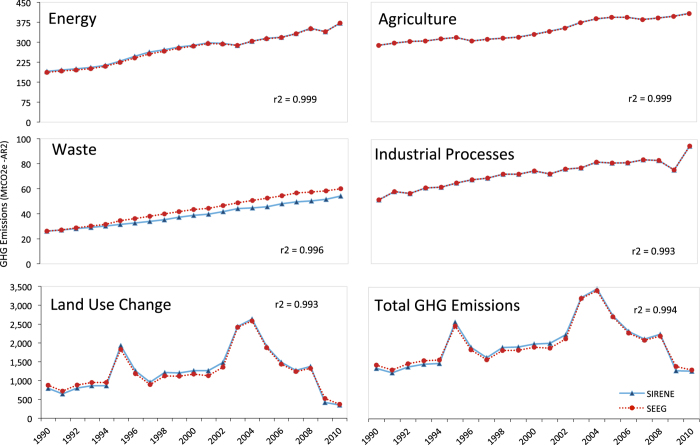
SEEG data validation against the 3^rd^ Brazilian National Inventory (MCTI, 2016), according to Global Warming Potential (GWP) of the IPCC’s Second Assessment Report (AR2 – IPCC).

**Figure 3:**
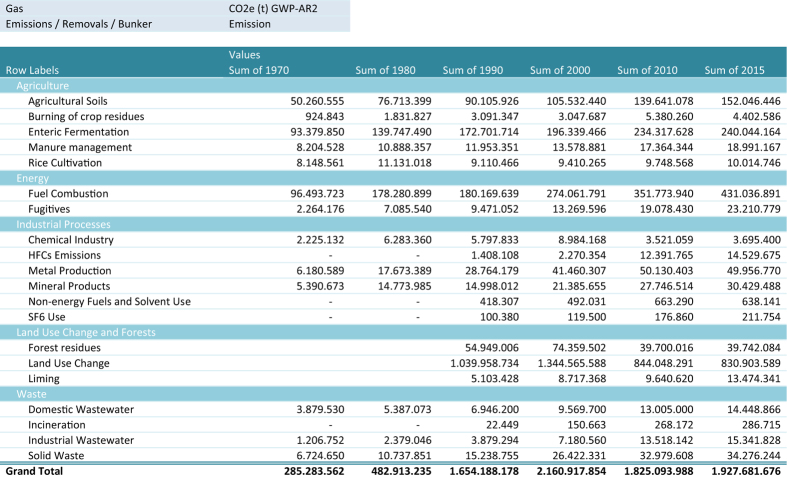
Example of national level data record available in SEEG.

The data language of this resource (Data Citation 1) is English and it is freely available through Figshare (http://figshare.com/). However, this dataset can also be found in Portuguese, freely available through the SEEG Platform (http://seeg.eco.br). As SEEG estimates and products are also constantly revised and improved, updated versions can be found in its website^11^. Data can be downloaded in one archive file (SEEG_GHG_dataset_1970_2015_ENG.xlsx). This file contains several metadata, separated by tabs of a spreadsheet, describing the structure of the data set as follows:

- *READ ME*: provides instructions for users to comprehensively navigate in the spreadsheet database.
- Dataset version history: keep records of all SEEG versions, their date of release, estimations timeframe and scope (national and/or sub-national levels) (Table 3).
- GEE Brazil (national level): SEEG national level dataset contains 293,807 data records organized in 6,352 rows and 57 columns for GHG emission and removal estimations described by sources and sinks, GHGs, geographical coverage (national level), economic activity, product and years (1970-2015) (Table 8).
- GHG Brazilian sub-national level: SEEG sub-national level dataset contains 3,087,226 data records organized in 67,632 rows and 57 columns for GHG emission and removal estimations described by sources and sinks, GHGs, geographical coverage (Brazilian states), economic activity, product and years (1970-2015) (Table 9).
- National level pivot-table: national data records were grouped in pivot-tables in order to allow the user to better visualize, separate, focus and/or make new data record combinations (Figure 2).
- Sub-national level pivot table: sub-national data records were grouped in a pivot-table in order to allow the user to better visualize, separate, focus and/or make new data record combinations (Figure 3).
- Logical tree: a logical-tree illustratively displays sectors and subsectors considered in the database.
- GWP and GTP values: contains Global Warming Potential (GWP) and Global Temperature Potential (GTP) values used in SEEG and their references (Table 2).
- GHG considered by sector: shows which GHG was estimated in each sector considered in the SEEG database (Table 1).
- States and non-allocated data: displays acronyms used in the SEEG database for Brazilian states and federal district (27 locations), non-allocated and international (bunker) emissions data (Table 3).
- Economic activity-related acronyms: describe economic activities acronyms used in the SEEG database (Table 4).
- Product-related acronyms: describe products acronyms used in the SEEG database (Table 5).

## Technical Validation

In order to assure the quality of the data generated, SEEG annually submits its methodology for peer-reviewing (consisting of members and non-members of the Climate Observatory) and holds a public seminar with relevant stakeholders of all sectors to disseminate and debate estimations.

In addition, SEEG records are also validated by comparing their estimates to the estimates reported by the BIs^8–10^, which covers the period of 1990-2010.

**Figure 4:**
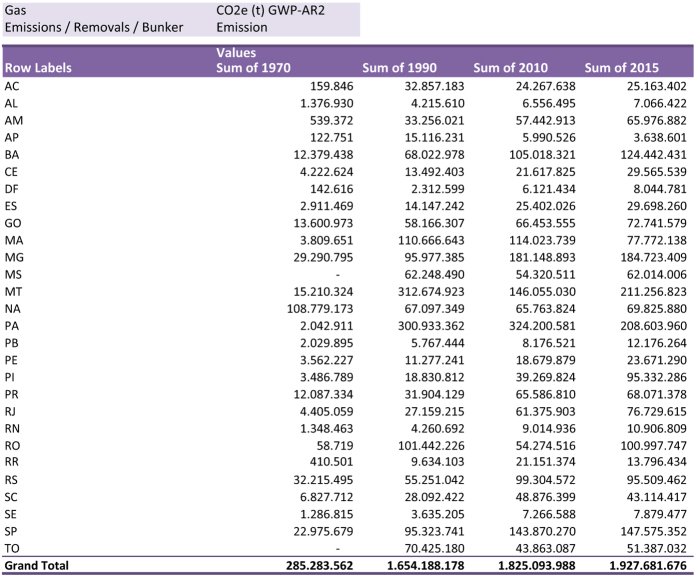
Example of sub-national level data record available in SEEG.

Yearly validations show less than 7% difference in total emissions (Figure 4) during this period. Among sectors, differences in GHG emissions between SEEG and BIs for the same time period are within 1% for the Agriculture sector, 3% for the Energy and Industrial Processes sectors, 15% for the Waste sector and 10% for LULUCF (with a25% outlier in 2009) (Figure 4).

For the waste sector, SEEG^8^ and the latest BI^10^ estimate divergences are mainly associated with utilization of different methodologies for estimating CH4 emissions from Solid Waste Disposal Sites (SWDS) and also with distinct values of activity data obtained for the amount of Municipal Solid Waste (MSW) generated, the amount of Clinical Waste incinerated and the industrial production observed for the period 1970-2010. The waste sector management in Brazil is marked by low data availability, which directly impacts the consistency and quality of the GHG emissions estimate.

### Overview on data quality

Between 1990 and 2015 gross GHG emissions in Brazil went from 1.62 to 1.93 GtCO2, an increase of almost 15%. In 2015, the Land Use sector emitted 46% of total emissions, followed by the Energy (23.6%), Agriculture (22.1%), Industrial Processes (5.2%) and Waste (3.3%) sectors (Figure 4).

Close to 80% of total SEEG GHG estimates are generated using Tier 2 emission factors and 86% of activity data used to generate these estimate came from accessible and available sources. This condition was able to provide 94% of overall estimates with good quality for 2015 and 97% of accumulated historical GHG emissions. Among sectors, estimates with good quality range from 100% to 77.1% for the Agricultural and Waste sectors, respectively. The most recent SEEG estimations allocated 96.5% of Brazilian national emissions in sub-national regions, which range from 77.8 to 100% depending on the sector (Table 10). Therefore, we judge SEEG system provides a reliable, robust and comprehensive long term dataset of GHG estimates in Brazil.

### Non-inventoried GHG emissions and removals

Removal of GHG by vegetation growth in the LUC sector represented 92% of the total removal recorded in the inventory and in the 2010 estimations^10^. In fact, unprotected forests can capture CO2, if they are in a natural renewal process, whereas forests in protected areas may emit CO2 if they are undergoing a process of degradation. Given the sheer volume that can represent – almost 400 million tons of CO2, or 30% of current emissions – this setting creates a distortion in the emissions data. From a conservative approach, the OC decided to prioritize the dissemination of SEEG data with gross emissions.

Thus, unless otherwise mentioned, all data presented here refer to gross emissions of GHG. In the query in the internet database the removal estimations according to the criteria used in the 2nd Brazilian emission inventory are also available. With this data on removals it is possible to estimate the net GHG emissions in Brazil. The estimations of SEEG did not incorporate the discount by emission reduction certificates originating from the Clean Development Mechanism^89^. The total in Brazil in the period 2005-2014, amounted to about 370 million tons of CO2e (accumulated period).

Regarding the soil carbon stock variation (emissions and removals of CO2) in agricultural areas, first-hand SEEG estimates show emissions in the agricultural sector can actually be 7% higher, a number that would impact total country GHG emissions by an additional 1.5%. This is mainly driven by the large area of degraded pasturelands in Brazil, estimated at 50 million hectares or 30% of the total Brazilian agricultural area.

## Usage Notes

This data set can be used to investigate GHG emissions in Brazil over more than 4 decades at the national and sub-national level for the following sectors: Agriculture, Energy, Industrial Processes and Product Use, Land Use Change and Waste. It may help to improve the understanding as well as anticipate trends related to GHG emissions and implications for public policies in Brazil.

## Additional information

**How to cite this article**: Azevedo, T. R. *et al.* SEEG initiative estimates of Brazilian greenhouse gas emissions from 1970 to 2015. *Sci. Data* 5:180045 doi: 10.1038/sdata.2018.45 (2018).

**Publisher’s note**: Springer Nature remains neutral with regard to jurisdictional claims in published maps and institutional affiliations.

## Supplementary Material



Supplementary Information

## Figures and Tables

**Table 1:** GHG emissions estimated by sector in SEEG.

	Gas	Sector				
		Agriculture	Energy	Industrial Processes	Waste	Land Use Change
Greenhouse Gases	CO2		X	X	X	X
	CH4	X				X
	N2O	X	X	X	X	X
	C2F6			X		
	CF4			X		
	HFC 125			X		
	HFC 143a			X		
	HFC 152a			X		
	HFC 23			X		
	HFC 134a			X		
Indirect Greenhouse Gases	CO	X	X	X		
	NMVOC		X	X		
	NOx	X	X	X		

**Table 2:** Global warming potential (GWP) and global temperature potential (GTP) values based on IPCC reports.

Gas	AR2 - Second Assessment Report (IPCC 1996)		AR4 - Fourth Assessment Report (IPCC 2007)		AR5 - Fifth Assessment Report (IPCC 2013)	
	GTP-100	GWP-100	GTP-100	GWP-100	GTP-100	GWP-100
CO2	1	1	1	1	1	1
CH4	5	21	5	25	4	28
N2O	270	310	270	298	234	265
HFC-23	12,700	11,700	12,700	14,800	12,700	12,400
HFC-32	94	650	94	675	94	677
HFC-125	1,113	2,800	1,113	3,500	967	3,170
HFC-134a	55	1,300	55	1,430	201	1,300
HFC-143a	4,288	3,800	4,288	4,470	2,500	4,800
HFC-152a	0	140	0	124	19	138
CF4	10,052	6,500	10,052	7,390	8,040	6,630
C2F6	22,468	9,200	22,468	12,200	13,500	11,100
SF6	40,935	23,900	40,935	22,800	28,200	23,500

**Table 3:** SEEG dataset version history.

Version	Date	Period Covered	Abrangency
SEEG 1.0	November 2013	1990–2012	National
SEEG 1.1	January 2014	1990–2012	National
SEEG 1.2	February 2014	1990–2012	National
SEEG 2.0	November 2014	1970–2013	National and sub-national allocation
SEEG 2.1	February 2015	1970–2013	National and sub-national allocation
SEEG 2.2	March 2015	1970–2013	National and sub-national allocation
SEEG 2.3	April 2015	1970–2013	National and sub-national allocation
SEEG 3.0	November 2015	1970–2014	National and sub-national allocation
SEEG 3.1	September 2016	1970–2014	National and sub-national allocation
SEEG 4.0	October 2016	1970–2015	National and sub-national allocation

**Table 4:** Acronyms of Brazilian federal units.

Initials	State
AC	Acre
AL	Alagoas
AP	Amapá
AM	Amazonas
BA	Bahia
CE	Ceará
DF	Distrito Federal
ES	Espírito Santo
GO	Goiás
MA	Maranhão
MT	Mato Grosso
MS	Mato Grosso do Sul
MG	Minas Gerais
PA	Pará
PB	Paraíba
PR	Paraná
PE	Pernambuco
PI	Piauí
RJ	Rio de Janeiro
RN	Rio Grande do Norte
RS	Rio Grande do Sul
RO	Rondônia
RR	Roraima
SC	Santa Catarina
SP	São Paulo
SE	Sergipe
TO	Tocantins
NA	Not allocated
INT	Intenacional
NA: non-allocated and bunker emissions used in SEEG.	

**Table 5:** Economic activities associated to GHG estimation in SEEG.

INITIALS	Economic Activity
AGROPEC	Agriculture
PEC	Livestock
AGR	Crops
MET	Metallurgy
CIM	Cement
TRAN_CARGA	Transport - Freigth
TRAN_PASS	Transport - Passenger
ENE_ELET	Power
COM	Buildings - Comercial
PUB	Buildings - Public Sector
RES	Buildings - Residential
PROD_COMB	Fuel Production
SANEAMENTO	Waste treatment
Outra_IND	Other Industries
Conservação	Conservation
HFC	Use of HFCs

**Table 6:** Product outputs associated to GHG estimations in SEEG.

Initials	Product
ALU	Aluminum and other non-iron based metals metallurgy
ACO	Iron and Steel
CAR	Beef
LEI	Milk
ALIM_BEBIDAS	Other Food and Beverages
ENE_ELET	Electricity

**Table 7:** Criteria for assess data quality of the GHG estimations of SEEG.

Aspect	National Estimates Replication (1990-2015)	
TIER (IPCC definition)	1	Tier 1
	2	Tier 2
	3	Tier 3
Activity data existence	1	activity data enable to assess level of calculation accuracy Tier 2
	2	data incomplete
	3	data do not exist
Activity data availability	1	open-source data
	2	partial open-source data (some restrictions may apply, i.e. needs to be purchased)
	3	data do not exist
Emission factor	1	explicit fator - with references
	2	implicit fator - correlation R2 equal or higher than 0,7
	3	implicit fator - correlation R2 lower than 0,7
Necessity for improvement	1	no need for improvement
	2	improvement is needed in the method OR in the activity data obtention
	3	improvement is needed in the method AND in the activity data obtention
General quality of data allocation	1	reliable data - able to reproduce the national inventory
	2	reliable data, however, some discrepancies may occur
	3	data with low reliability
Aspect	Data Subnational Allocation	
Data allocation ocurrence	1	Allocation of all GHG emission results was possible
	2	Allocation partially possible. Some of the GHG emission results could not be allocate
	3	Allocation of the GHG emission could not be allocated.
Allocation criteria	1	Allocation criteria direct linked to the related emission factor
	2	Allocation criteria uses indirect factors strongly linked to direct factors.
	3	Allocation criteria uses indirect factors poorly linked to direct factors.
Activity data existence	1	activity data enable to assess level of calculation accuracy Tier 2
	2	data incomplete
	3	data do not exist
Activity data avaiability	1	open-source data
	2	partial open-source data (some restrictions may apply, i.e. needs to be purchased)
	3	data do not exist
Emission factor	1	explicit fator - with references
	2	implicit fator - correlation R2 equal or higher than 0,7
	3	implicit fator - correlation R2 lower than 0,7
Necessity for improvement	1	no need for improvement
	2	improvement is needed in the method OR in the activity data obtention
	3	improvement is needed in the method AND in the activity data obtention
General quality of data allocation	1	reliable data - able to reproduce the national inventory
	2	reliable data, however, some discrepancies may occur
	3	data with low reliability
Aspect	General Aspect of Historical Estimates (pre-national inventory - 1970-1989)	
General quality of data allocation	1	reliable data - able to reproduce the national inventory
	2	reliable data, however, some discrepancies may occur
	3	data with low reliability

**Table 8:** Example of national level data record available in SEEG.

Level 1	Level 2	Level 3	Level 4	Level 5	Level 6	Emission / Removal / Bunker	Gas	State/Province	1970	...	2015
Energy	Fuel Combustion Emissions	Transport	Road	Diesel Oil	Trucks	Emission	CO2 (t)	BR	7,739,379	...	83,993,746
Energy	Fuel Combustion Emissions	Transport	Road	Diesel Oil	Light Commercials	Emission	CO2 (t)	BR	18,241	...	5,518,895
Energy	Fuel Combustion Emissions	Transport	Road	Diesel Oil	Buses	Emission	CO2 (t)	BR	4,292,582	...	19,507,832
Energy	Fuel Combustion Emissions	Transport	Road	Motor Gasoline	Cars	Emission	CO2 (t)	BR	17,160,466	...	54,493,607

**Table 9:** Example of sub-national level data record available in SEEG.

Level 1	Level 2	Level 3	Level 4	Level 5	Level 6	Emissions / Removals	Gas	State/Province	Economic Activity	Produto	1970	...	2015
Agriculture	Enteric Fermentation	Direct	Others	Animal	Beef Cattle	Emission	CH4 (t)	RO	PEC	CAR	1.026.40	...	577.612.70
Agriculture	Enteric Fermentation	Direct	Others	Animal	Beef Cattle	Emission	CH4 (t)	AC	PEC	CAR	3.031.10	...	130.996.40
Agriculture	Enteric Fermentation	Direct	Others	Animal	Beef Cattle	Emission	CH4 (t)	AM	PEC	CAR	11.287.30	...	61.083.50
Agriculture	Enteric Fermentation	Direct	Others	Animal	Beef Cattle	Emission	CH4 (t)	RR	PEC	CAR	10.474.20	...	33.435.10
Agriculture	Enteric Fermentation	Direct	Others	Animal	Beef Cattle	Emission	CH4 (t)	PA	PEC	CAR	47.576.70	...	935.226.00
Agriculture	Enteric Fermentation	Direct	Others	Animal	Beef Cattle	Emission	CH4 (t)	AP	PEC	CAR	2.919.40	...	7.777.70
Agriculture	Enteric Fermentation	Direct	Others	Animal	Beef Cattle	Emission	CH4 (t)	TO	PEC	CAR	-	...	365.450.10
Agriculture	Enteric Fermentation	Direct	Others	Animal	Beef Cattle	Emission	CH4 (t)	MA	PEC	CAR	77.268.20	...	357.749.20
Agriculture	Enteric Fermentation	Direct	Others	Animal	Beef Cattle	Emission	CH4 (t)	PI	PEC	CAR	60.396.10	...	76.674.80
Agriculture	Enteric Fermentation	Direct	Others	Animal	Beef Cattle	Emission	CH4 (t)	CE	PEC	CAR	78.487.00	...	95.676.10
Agriculture	Enteric Fermentation	Direct	Others	Animal	Beef Cattle	Emission	CH4 (t)	RN	PEC	CAR	27.631.30	...	34.958.30
Agriculture	Enteric Fermentation	Direct	Others	Animal	Beef Cattle	Emission	CH4 (t)	PB	PEC	CAR	38.704.80	...	45.965.50
Agriculture	Enteric Fermentation	Direct	Others	Animal	Beef Cattle	Emission	CH4 (t)	PE	PEC	CAR	55.984.50	...	73.378.60
Agriculture	Enteric Fermentation	Direct	Others	Animal	Beef Cattle	Emission	CH4 (t)	AL	PEC	CAR	24.101.40	...	56.198.60
Agriculture	Enteric Fermentation	Direct	Others	Animal	Beef Cattle	Emission	CH4 (t)	SE	PEC	CAR	30.038.70	...	50.914.70
Agriculture	Enteric Fermentation	Direct	Others	Animal	Beef Cattle	Emission	CH4 (t)	BA	PEC	CAR	275.917.40	...	430.762.40
Agriculture	Enteric Fermentation	Direct	Others	Animal	Beef Cattle	Emission	CH4 (t)	MG	PEC	CAR	613.633.50	...	865.214.40
Agriculture	Enteric Fermentation	Direct	Others	Animal	Beef Cattle	Emission	CH4 (t)	ES	PEC	CAR	56.423.50	...	88.384.80
Agriculture	Enteric Fermentation	Direct	Others	Animal	Beef Cattle	Emission	CH4 (t)	RJ	PEC	CAR	48.163.60	...	93.953.90
Agriculture	Enteric Fermentation	Direct	Others	Animal	Beef Cattle	Emission	CH4 (t)	SP	PEC	CAR	398.934.30	...	431.682.80
Agriculture	Enteric Fermentation	Direct	Others	Animal	Beef Cattle	Emission	CH4 (t)	PR	PEC	CAR	214.738.50	...	417.624.50
Agriculture	Enteric Fermentation	Direct	Others	Animal	Beef Cattle	Emission	CH4 (t)	SC	PEC	CAR	83.392.50	...	182.678.00
Agriculture	Enteric Fermentation	Direct	Others	Animal	Beef Cattle	Emission	CH4 (t)	RS	PEC	CAR	631.956.30	...	753.710.10
Agriculture	Enteric Fermentation	Direct	Others	Animal	Beef Cattle	Emission	CH4 (t)	MS	PEC	CAR	-	...	1.008.752.60
Agriculture	Enteric Fermentation	Direct	Others	Animal	Beef Cattle	Emission	CH4 (t)	MT	PEC	CAR	463.836.30	...	1.390.512.20
Agriculture	Enteric Fermentation	Direct	Others	Animal	Beef Cattle	Emission	CH4 (t)	GO	PEC	CAR	340.710.30	...	924.350.10
Agriculture	Enteric Fermentation	Direct	Others	Animal	Beef Cattle	Emission	CH4 (t)	DF	PEC	CAR	1.244.60	...	3.852.10

**Table 10:** Total Brazilian GHG emissions and percentage able to be allocated sub-nationally in SEEG for 2015.

Sector	Total GHG emissions MtCO2e	Total GHG emissions allocated sub-nationally %
Energy	454.2	91.7
Industrial Processes	99.5	77.8
Waste	64.4	99.9
Agriculture	425.5	100.0
Land Use Change and Forests	884.1	99.9
Total	1927.7	96.5
