# Supplementary material for: SEEG initiative estimates of Brazilian greenhouse gas emissions from 1970 to 2015
Source: Sci Data. 2018 May 29;5:180045. doi: 10.1038/sdata.2018.45 (PMC5972695; doi:10.1038/sdata.2018.45)
Supplement: Supplementary Information [file sdata201845-s2.docx]

**Supplementary File 1**

**Contents**

**List of Figures**

| S1 | Greenhouse gas (GHG) emissions estimates structure by emission type, energy  source and emission-generating activities………………………………………………………………………. | 2 |
| --- | --- | --- |
| S2 | Data processing sequence used in CO_2_ emissions estimates from fuel combustion………… | 3 |
| S3 | Data processing sequence used in CH_4_, N_2_O, CO, NO_x_ and NMVOC emissions estimates… | 3 |
| S4 | Greenhouse gas (GHG) emissions estimates structure for industrial processes and product uses, products and inputs and emitted gases according to IPCC (2006)…………….. | 4 |
| S5 | Pig iron and steel production simplified representation and emitting processes……………. | 5 |
| S6 | Metallic aluminum production and related emissions……………………………………………………. | 5 |
| S7 | Cement production and related greenhouse gas (GHG) emissions………………………………… | 5 |
| S8 | Lime production and related greenhouse gas (GHG) emissions………………………………………. | 6 |
| S9 | Glass production and related greenhouse gas (GHG) emissions……………………………………… | 6 |
| S10 | Regional methane generation potential (*L_0_*) for each type of solid waste disposal  sites (SWDS) in Brazil……………………………………………………………………………………………………… | 7 |
| S11 | Relation between deforestation and emissions of CO_2_ from 1990 to 2002…………………….. | 8 |

**List of Tables**

| S1 | Activity data for estimation of soil carbon variation under conventional (CT)  and no-tillage cropping systems (NT) in Brazil from 1990 to 2015………………………………….. | 9 |
| --- | --- | --- |
| S2 | Activity data for estimation of the soil carbon variation of commercial  planted forests in Brazil from 1990 to 2015……………………………………………………………………. | 10 |
| S3 | Activity data and emission and removal factors for estimating soil carbon  variation under different soil use and management in agriculture in Brazil for 2015……… | 11 |
| S4 | Information sources for subnational emission estimations…………………………………………….. | 12 |
| S5 | Chemical production data sources……………………………………………………….………………………. | 13 |
| S6 | Chemical substances and emitted GHG in its production processes....……………………………. | 14 |
| S7 | Coefficients for estimating DOC(t)………………………………………………………………………………….. | 15 |
| S8 | Raw emissions of carbon dioxide per biome………………………………………………………….……….. | 15 |
| S9 | Average annual deforestation for the period of from 1994 to 2002……………………..………… | 15 |
| S10 | Deforestation data available for the period of 2006 to 2015………………………………..………… | 16  16 |
| S11 | Proportion of the annual deforestation from PRODES (period 1994-2002) based  on the average deforestation for the same period from the Brazilian  National Inventory…………………………………………………………………………………………………………. | 16 |
| S12 | Example of estimated gross emissions based on the National Inventory data  for the Amazon biome based on 1994-2002 data…………………………………………………………... | 17 |


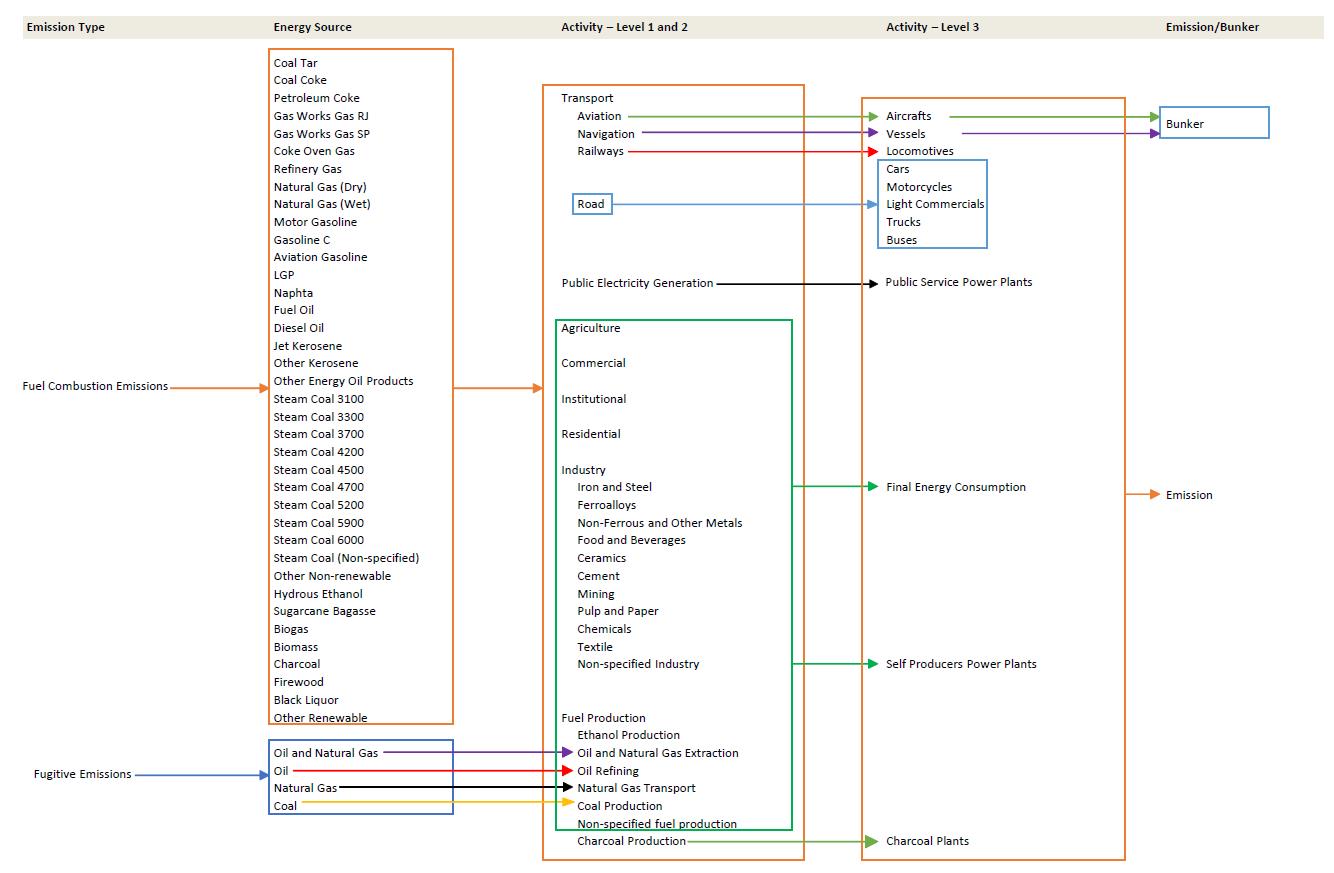


Figure S1. Greenhouse gas (GHG) emissions estimates structure by emission type, energy source and emission-generating activities


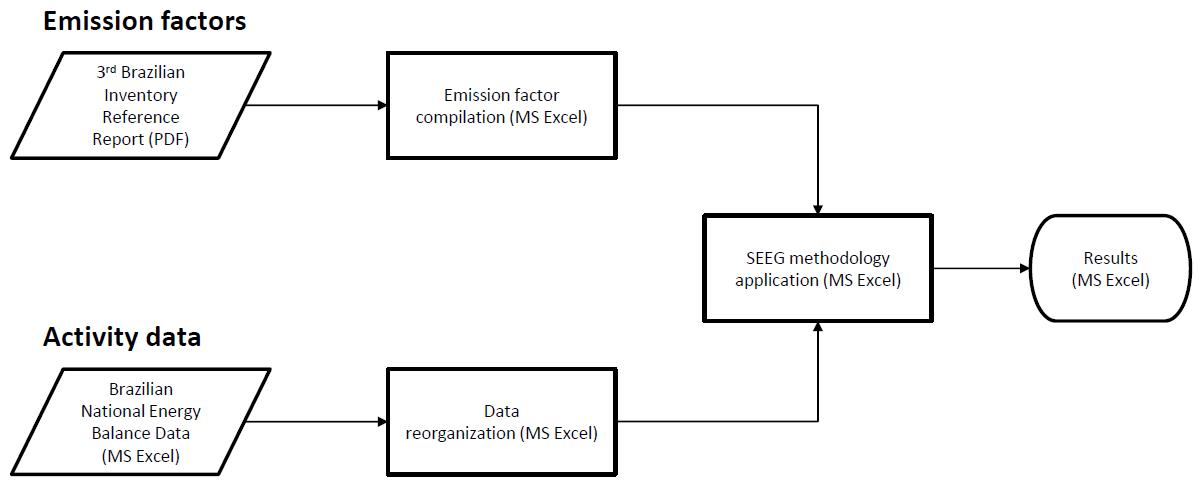


Figure S2. Data processing sequence used in CO_2_ emissions estimates from fuel combustion


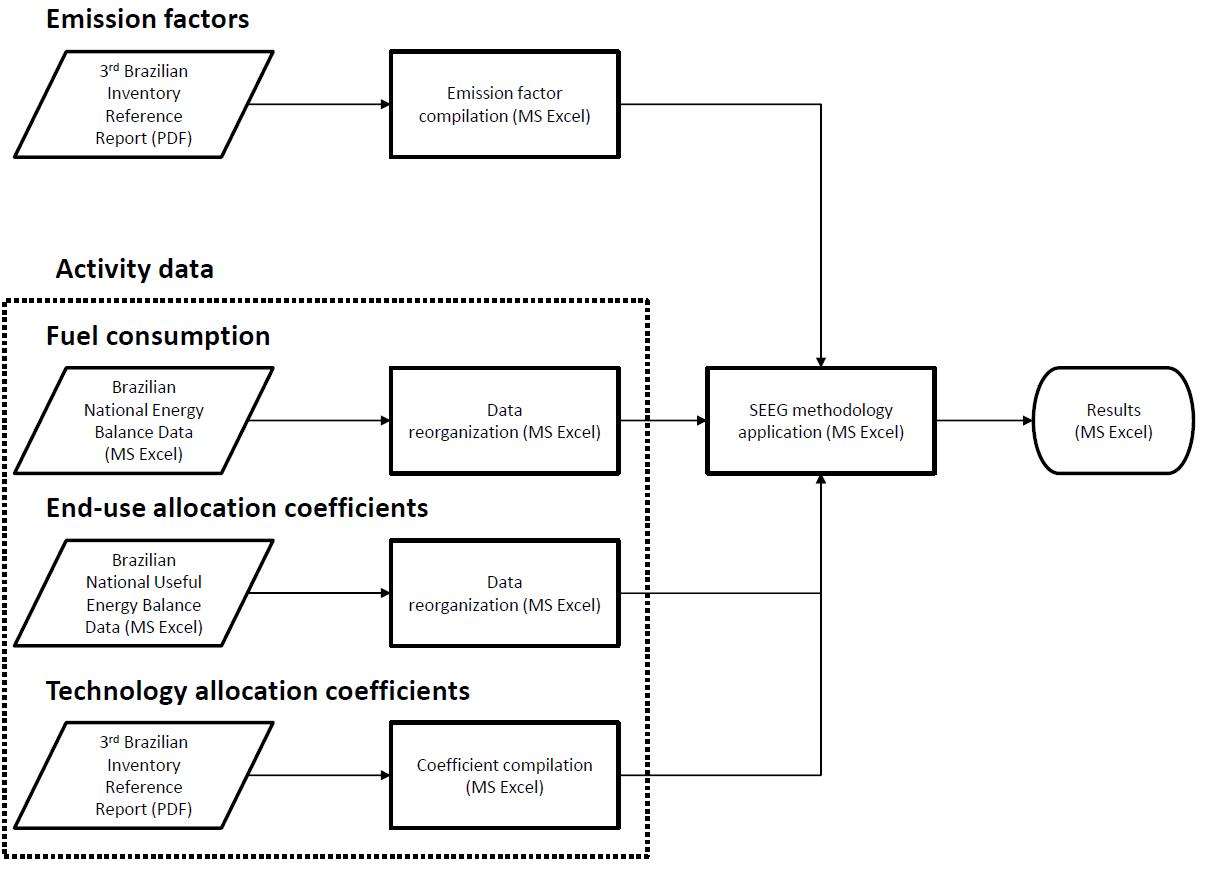


Figure S3. Data processing sequence used in CH_4_, N_2_O, CO, NO_x_ and NMVOC emissions estimates


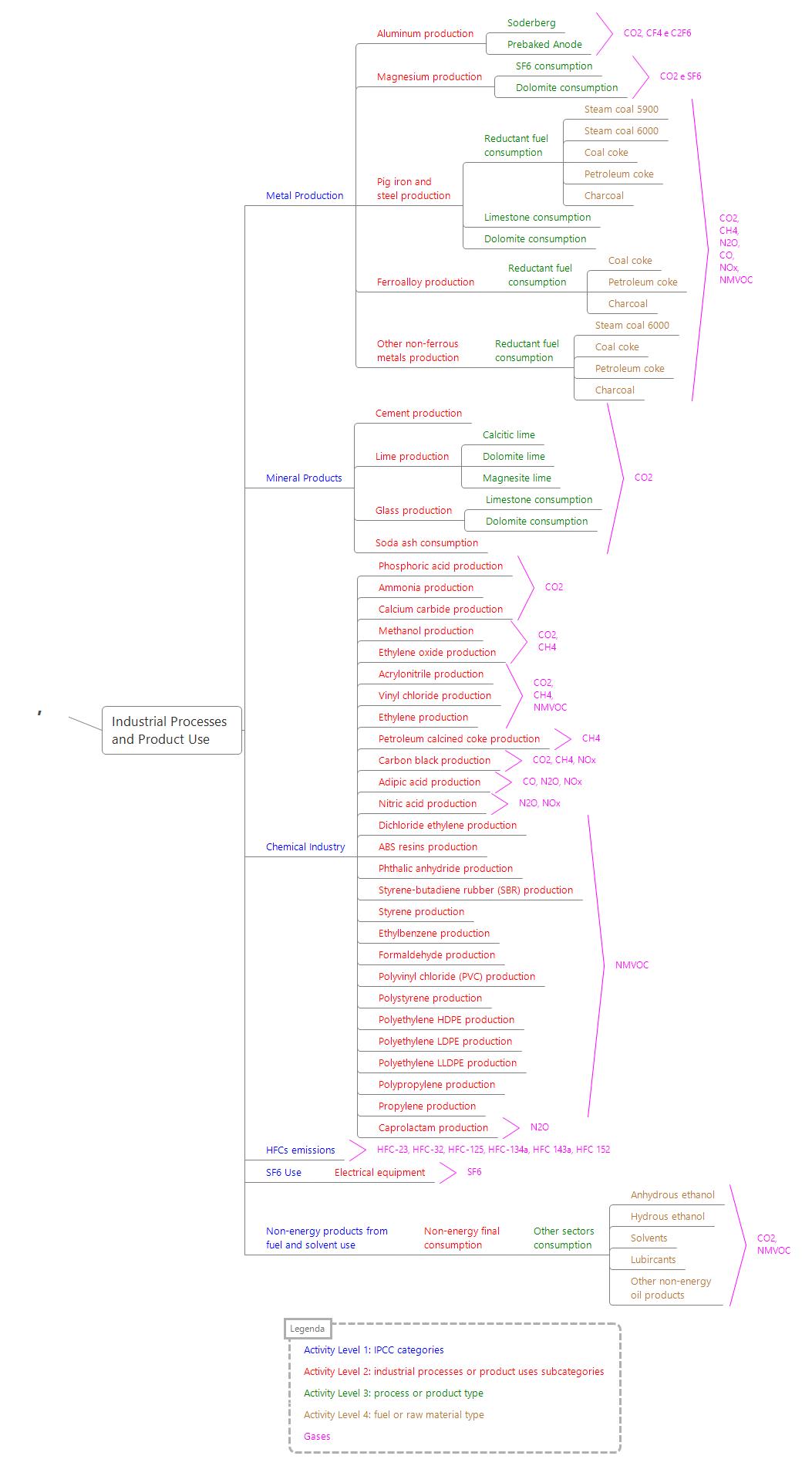


Figure S4. Greenhouse gas (GHG) emissions estimates structure for industrial processes and product uses, products and inputs and emitted gases according to IPCC^1^


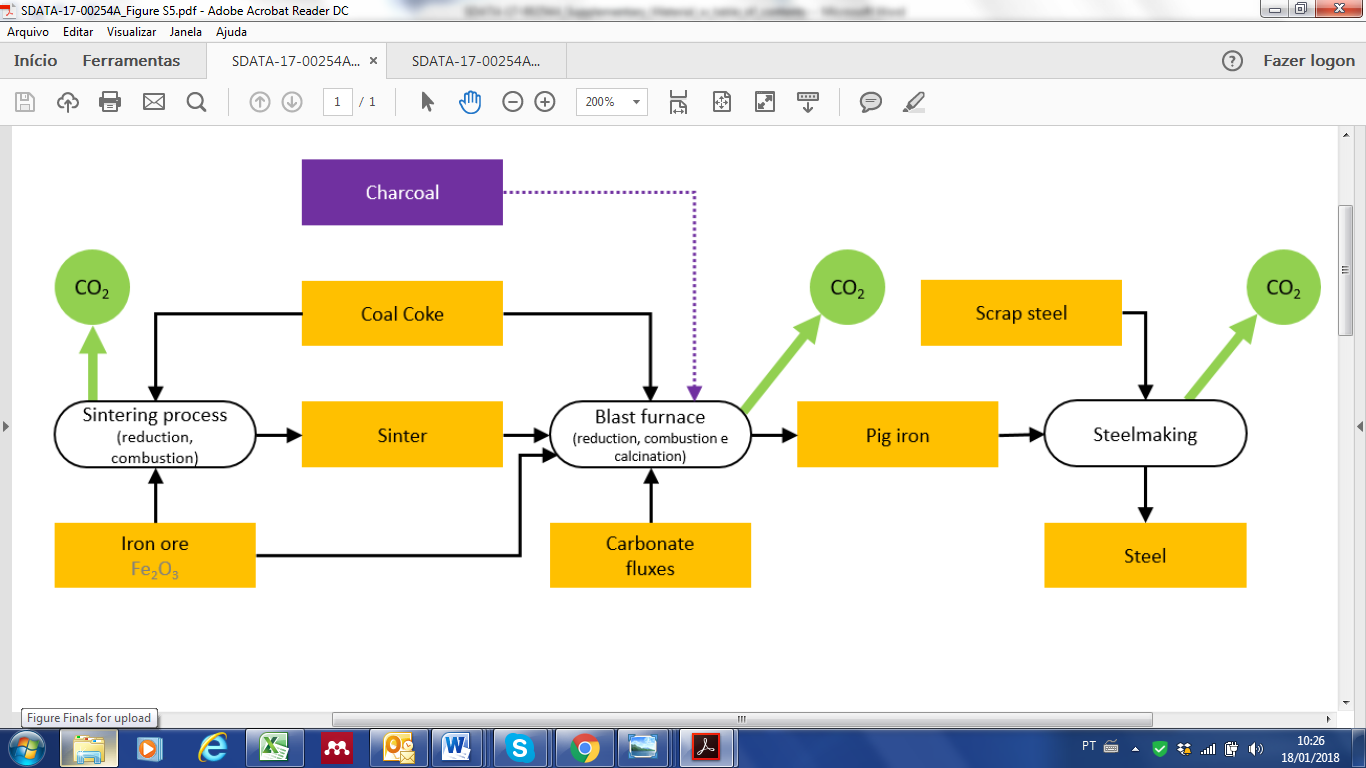


Figure S5. Pig iron and steel production simplified representation and emitting processes


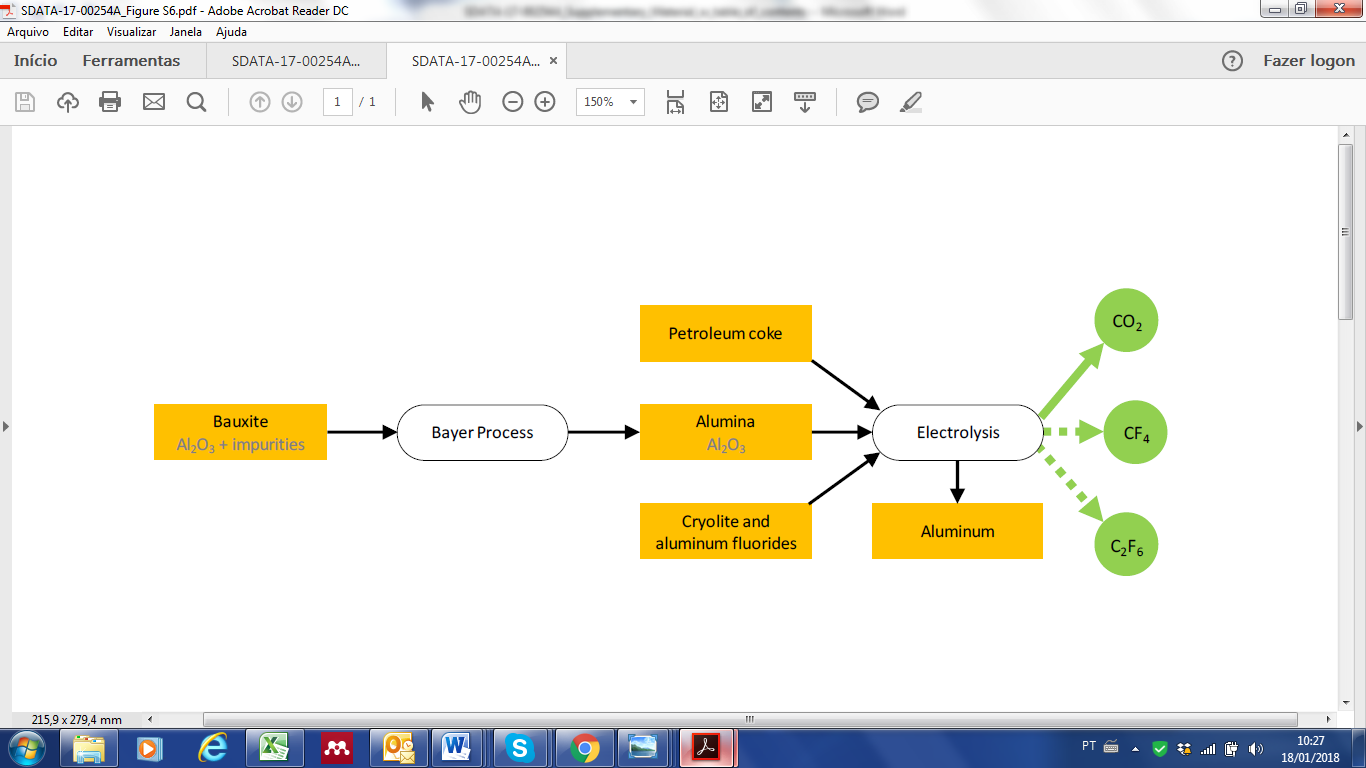


Figure S6. Metallic aluminum production and related emissions


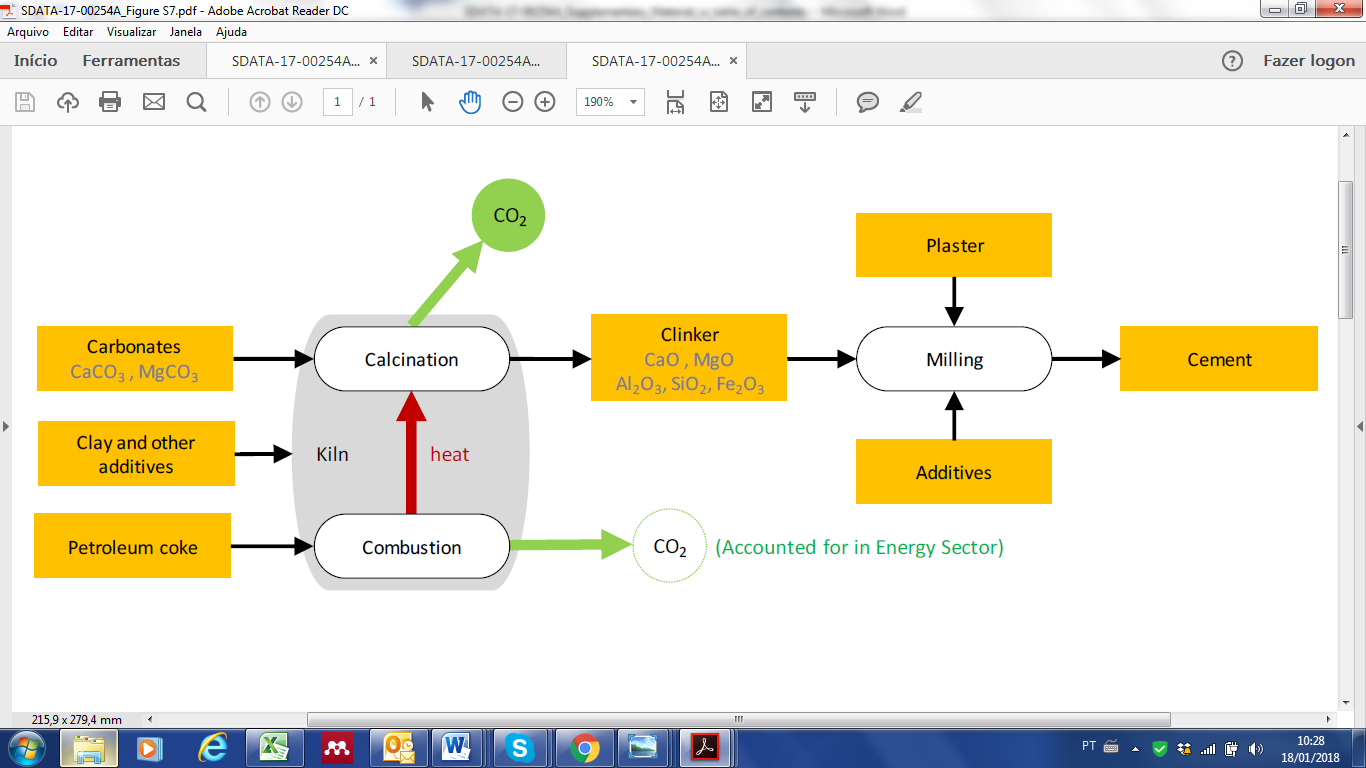


Figure S7. Cement production and related greenhouse gas (GHG) emissions


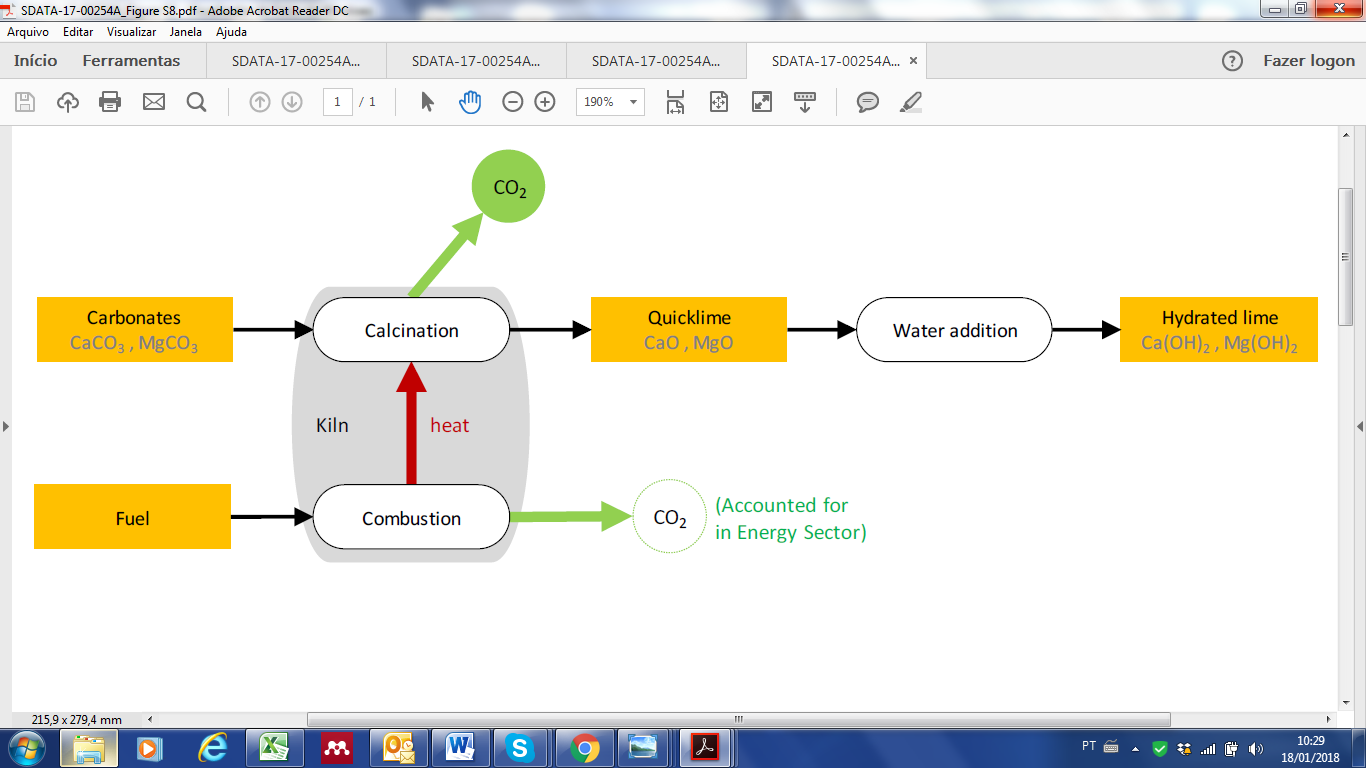


Figure S8. Lime production and related greenhouse gas (GHG) emissions


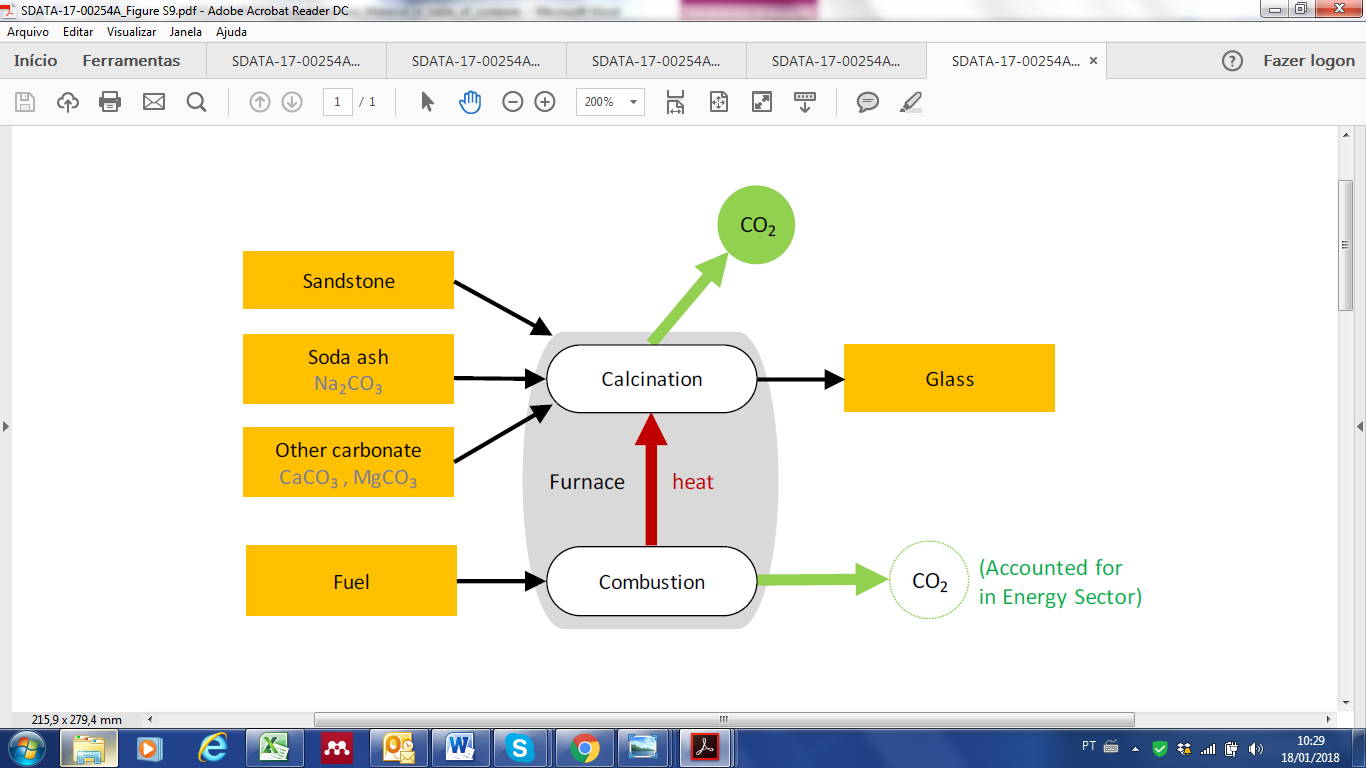


Figure S9. Glass production and related greenhouse gas (GHG) emissions


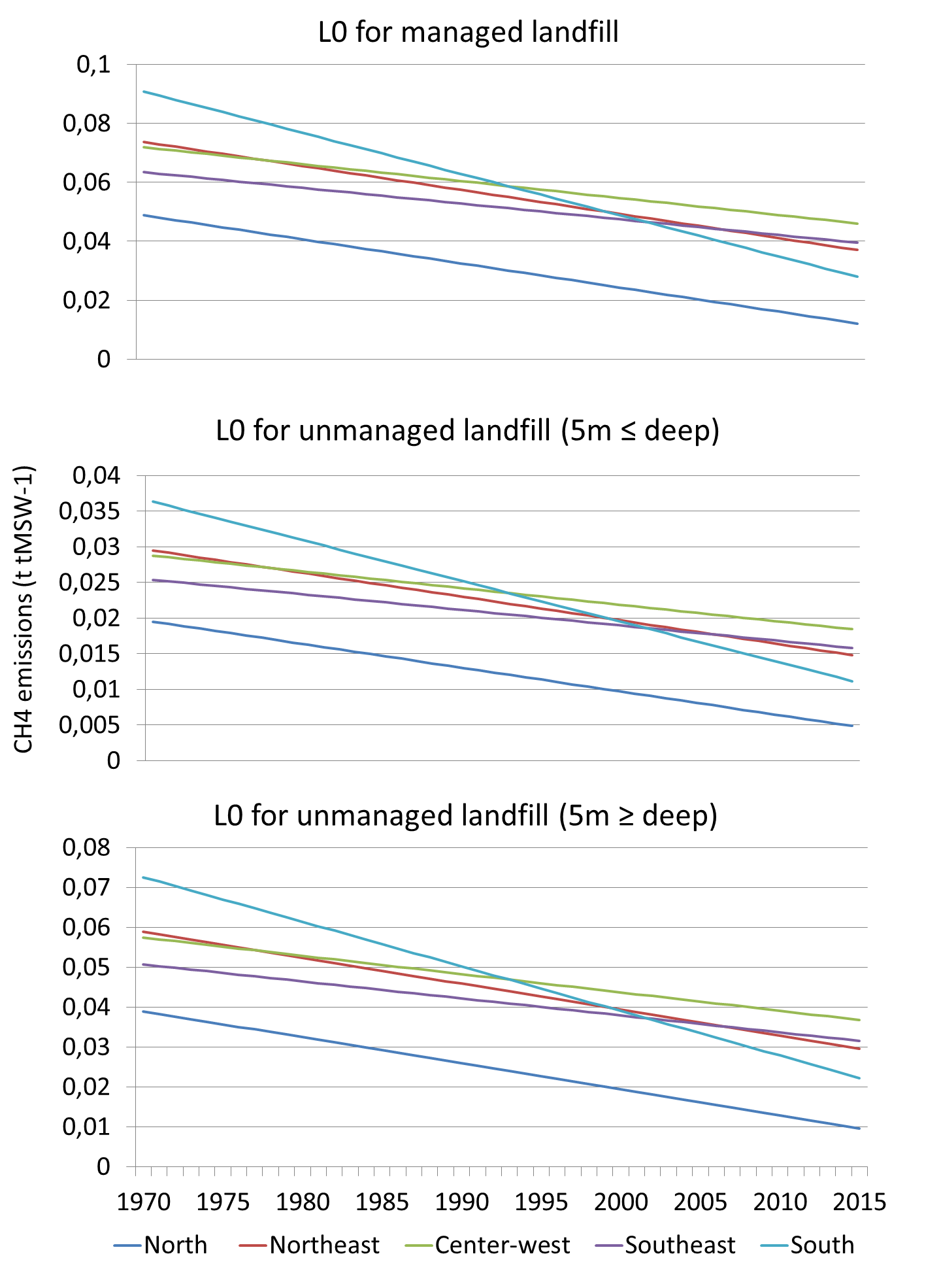


Figure S10. Regional methane generation potential (*L_0_*) for each type of solid waste disposal sites (SWDS) in Brazil* *MSWt: Total municipal solid waste


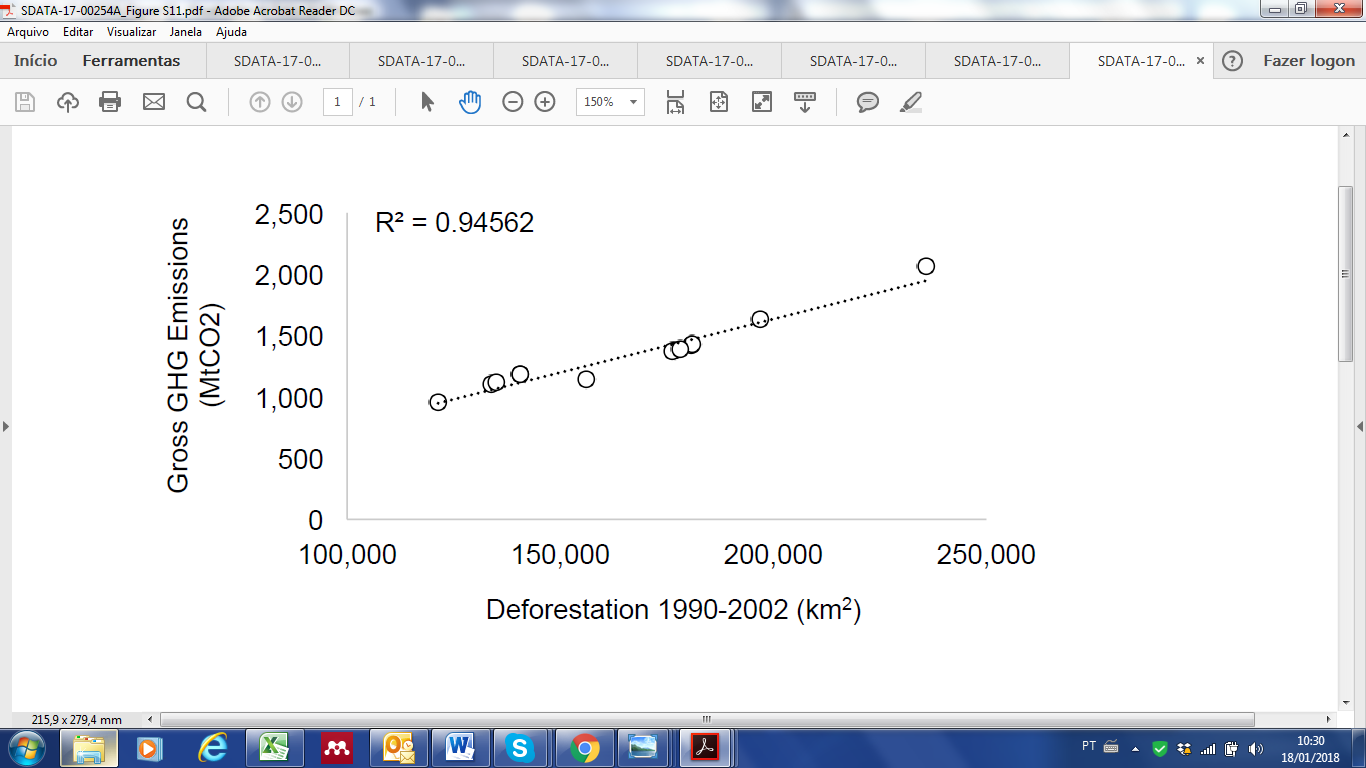


Figure S11. Relation between deforestation and emissions of CO_2_ from 1990 to 2002^2^

Table S1. Activity data for estimation of soil carbon variation under conventional and no-tillage cropping systems in Brazil from 1990 to 2015^3,4^

| Tillage System (million hectare) | 1990 | 1991 | 1992 | 1993 | 1994 | 1995 | 1996 | 1997 | 1998 | 1999 | 2000 | 2001 | 2002 | 2003 | 2004 | 2005 | 2006 | 2007 | 2008 | 2009 | 2010 | 2011 | 2012 | 2013 | 2014 | 2015 |
| --- | --- | --- | --- | --- | --- | --- | --- | --- | --- | --- | --- | --- | --- | --- | --- | --- | --- | --- | --- | --- | --- | --- | --- | --- | --- | --- |
| Conventional Tillage | 0.9 | 1 | 1.4 | 2 | 3 | 3.8 | 5.5 | 8.8 | 11.3 | 13.4 | 14.3 | 17.4 | 18.7 | 20.2 | 21.9 | 23.6 | 25.5 | *26.6* | *27.9* | *29.8* | *31.7* | *26.4* | 31.8 | *34.5* | *39.7* | *40.9* |
| No-Tillage | *38.0* | *36.9* | *37.1* | *33.6* | *36.1* | *34.7* | *31.5* | *27.8* | *23.7* | *23.5* | *23.5* | *20.4* | *21.5* | *23.7* | *25.5* | *25.5* | *22.4* | *19.7* | *19.5* | *17.8* | *15.7* | *23.5* | *19.1* | *19.0* | *17.4* | *17.0* |
| Total area | 38.9 | 37.9 | 38.5 | 35.6 | 39.1 | 38.5 | 37 | 36.6 | 35 | 36.9 | 37.8 | 37.8 | 40.2 | 43.9 | 47.4 | 49.1 | 47.9 | 46.2 | 47.4 | 47.7 | 47.4 | 49.9 | 50.9 | 53.6 | 57.1 | 57.9 |

Areas in italic represent estimations made by SEEG based on the previous references

Table S2. Activity data for estimation of the soil carbon variation of commercial planted forests in Brazil from 1990 to 2015^5-7^

Numbers in italic represent estimations made by SEEG based on the previous references; NA = not allocated

Table S3. Activity data and emission and removal factors for estimating soil carbon variation under different soil use and management in agriculture in Brazil for 2015

| Land use |  | Referência |
| --- | --- | --- |
| Area (mi ha) | | |
| Pasture | 175.0 | Expert consultation |
| Stable | 106.0 | Expert consultation |
| Degraded | 50.0 | Expert consultation |
| Well-managed | 19.0 | Expert consultation |
| Integrated Systems  (Crop-livestock-forest) | 1.6 | EMBRAPA^8^ |
| Planted Forest | 7.8 | IBÁ^5^; ABRAF^6,7^ |
| Cropping | 57.9 | CONAB^3^ |
| Conventional system (CT) | 17.0 | (difference between CT and NT) |
| No tillage system (NT) | 40.9 | FEBRAPDP^4^ |
| Emission and Removal factors (tCO_2_e ha-1 y-1)* | | |
| Pasture |  |  |
| Stable | 0.00 | - |
| Degraded | 4.00 | Observatório ABC^9^ |
| Well-managed | -5.51 | Bustamante et al.^10^ |
| Integrated Systems  (Crop-livestock-forest) | -6.24 | Carvalho et al.^11^ |
| Planted Forest | -0.81 | Lima et al.^12^ |
| Cropping |  |  |
| Conventional system (CT) | 1.47 | Costa Junior et al.^13^ |
| No tillage system (NT) | -1.84 | Cerri et al.^14^ |

- Negative values represent CO_2_ removal (soil carbon sequestration)

Table S4. Information sources for subnational emission estimations

*OEOP: Other Energy Oil Product; ONR: Other Non-Renewable;OR: Other Renewable

Table S5: Chemical production data sources

| Period | Information Source |
| --- | --- |
| 1970-1971 | Brazilian Chemical Industry Yearbook^23^ |
| 1972-1973 | Brazilian Chemical Industry Yearbook^24^ |
| 1974-1984 | Brazilian Chemical Industry Yearbook^25^ |
| 1985-1989 | Brazilian Chemical Industry Yearbook^26^ |
| 1990-2010 | MCTI^27^ |
| 2011-2014 | Brazilian Chemical Industry Yearbook^28^ |
| 2015 | 2014 production kept constant |

Table S6: Chemical substances and emitted GHG in its production processes

| Year | CO_2_ | CH_4_ | N_2_O | CO | NO_x_ | NMVOC |
| --- | --- | --- | --- | --- | --- | --- |
| Ammonia | X |  |  |  |  |  |
| Nitric Acid |  |  | X |  | X |  |
| Adipic Acid |  |  | X | X | X |  |
| Caprolactam |  |  | X |  |  |  |
| Calcium Carbide | X |  |  |  |  |  |
| Methanol | X | X |  |  |  |  |
| Ethylene | X | X |  |  |  | X |
| Dichloride Ethylene and Vinyl Chloride | X | X |  |  |  | X |
| Ethylene Oxide | X | X |  |  |  |  |
| Acrylonitrile | X | X |  |  |  | X |
| Petroleum Calcined Coke |  | X |  |  |  |  |
| Carbon Black | X | X |  |  | X |  |
| Phosphoric Acid | X |  |  |  |  |  |
| Other Chemical Products* |  |  |  |  |  | X |

*ABS resins, phthalic anhydride, styrene-butadiene rubber (SBR), styrene, ethylbenzene, formaldehyde, polyvinyl chloride (PVC), polystyrene, polyethylene (HDPE, LDPE, LLDPE), polypropylene and propylene production

Table S7: Coefficients for estimating DOC(t)^29^

| Region | Angular (a) | Linear (b) |
| --- | --- | --- |
| North | -0,00244391 | 4,96084385 |
| Northeast | -0,00244391 | 5,03562584 |
| Central-west | -0,00172315 | 3,61033161 |
| Southeast | -0,00159636 | 3,33517388 |
| South | -0,00419093 | 8,52847581 |

Table S8. Raw emissions of carbon dioxide per biome^30^

| Biomes | Total (tCO_2_) | Average 1994-2002 (tCO_2_) |
| --- | --- | --- |
| Amazon | 8,465,226,000 | 1,058,153,250 |
| Cerrado | 2,622,510,540 | 327,813,817 |
| Caatinga | 343,820,831 | 42,977,604 |
| Atlantic Forest | 728,886,016 | 91,110,752 |
| Pampa | 136,159,616 | 17,019,952 |
| Pantanal | 757,050 | 94,631 |

Table S9. Average annual deforestation for the period of from 1994 to 2002^30^

| Biomes | Average 1994-2002 (km²/year) |
| --- | --- |
| Amazon | 19,141 |
| Cerrado | 15,698 |
| Caatinga | 5,905 |
| Atlantic Forest | 2,617 |
| Pampa | 2 |
| Pantanal | 962 |

Table S10. Deforestation data available for the period of 2006 to 2015.

| Biome | 2006 | 2007 | | 2008 | | 2009 | 2010 | 2011 | 2012 | 2013 | 2014 | 2015 | Source |
| --- | --- | --- | --- | --- | --- | --- | --- | --- | --- | --- | --- | --- | --- |
| Amazon | 1 | 1 | | 1 | | 1 | 1 | 1 | 1 | 1 | 1 | 1 | Prodes^31^ |
| Caatinga | 2 | 2 | | 2 | | 1 | 3 | 3 | 3 | 3 | 3 | 3 | PMDBB S^32^ |
| Cerrado | 2 | 2 | | 2 | | 1 | 1 | 3 | 3 | 3 | 3 | 3 | PMDBB S^32^ |
| Atlantic F. Forest | 2 | 2 | | 2 | | 1 | 3 | 3 | 3 | 3 | 3 | 3 | SOS Mata Atlântica^33^ |
| Pampa | 2 | 2 | | 2 | | 1 | 3 | 3 | 3 | 3 | 3 | 3 | PMDBB S^32^ |
| Pantanal | 2 | 2 | | 2 | | 1 | 3 | 3 | 3 | 3 | 3 | 3 | PMDBB S^32^ |
| 1 |  | |  | | Annual deforestation data | | | | | | | | |
| 2 |  | |  | | Estimate of average annual deforestation | | | | | | | | |
| 3 |  | |  | | Nonexistent data – in this case we repeated the last available data | | | | | | | | |

Table S11. Proportion of the annual deforestation from PRODES^31^ based on the average deforestation for the same period from the Brazilian National Inventory^2^.

| Biome | Data | 2006 | 2007 | 2008 | 2009 | 2010 | 2011 | 2012 | 2013 | 2014 |
| --- | --- | --- | --- | --- | --- | --- | --- | --- | --- | --- |
| Amazon | Deforestation (km²) | 14,286 | 11,651 | 12,911 | 7,464 | 7,000 | 6,418 | 4,571 | 5,891 | 5,012 |
|  | Proportion of average annual deforestation (1994-2002) | 0.75 | 0.61 | 0.67 | 0.39 | 0.37 | 0.34 | 0.24 | 0.31 | 0.26 |

Table S12. Example of estimated gross emissions based on the National Inventory data for the Amazon biome, period 1994-2002

| Biome | Data | 2006 | 2007 | 2008 | 2009 | 2010 | 2011 | 2012 | 2013 | 2014 |
| --- | --- | --- | --- | --- | --- | --- | --- | --- | --- | --- |
|  | Raw |  |  |  |  |  |  |  |  |  |
| Amazon | emissions | 789.7 | 644.0 | 713.7 | 412.6 | 386.9 | 354.7 | 252.6 | 325.6 | 277.0 |
|  | (tCO_2_) | 40 | 80 | 30 | 20 | 70 | 93 | 89 | 66 | 73 |
|  |  |  |  |  |  |  |  |  |  |  |

**References**

1. Intergovernamental Panel on Climate Change. *IPCC Guidelines for National Greenhouse Gas Inventories* <http://www.ipcc-nggip.iges.or.jp/public/2006gl> (2006).

2. Ministério da Ciência e Tecnologia e Inovação (MCTI). *Emissões e Remoções de Gases de Efeito Estufa pela Mudança do Uso da Terra e Florestas. Relatório de Referência: Setor Uso da Terra, Mudança do Uso da Terra e Florestas. Terceiro Inventario Brasileiro de Emissões e Remoções Antrópicas de Gases de Efeito Estufa* http://sirene.mcti.gov.br/publicacoes (2015).

3.Companhia Nacional de Abastecimento. *Séries Históricas* <http://www.conab.gov.br/conteudos.php?a=1252&> (2015).

4. Federação Brasileira de Plantio Direto na Palha. *Área do Sistema Plantio Direto*. <http://febrapdp.org.br/download/PD_Brasil_2013.jpg>

5.Indústria Brasileira de Árvores. *Relatório Anual da IBÁ* <http://www.florestal.gov.br/snif/recursos-florestais/as-florestas-plantadas> (2016).

6. Associação Brasileira de Florestas Plantadas. *Anuário estatístico da ABRAF 2010 - ano base 2009* <http://www.ipef.br/estatisticas/relatorios/anuario-ABRAF-2010-BR.pdf> (2009).

7. Associação Brasileira de Florestas Plantadas. *Anuário estatístico da ABRAF: ano base 2005* <http://www.ipef.br/estatisticas/relatorios/anuario-ABRAF-2010-BR.pdf> (2006).

8. Empresa Brasileira de Pesquisa Agropecuária. *Marco Referencial; Integração lavoura-pecuária-floresta* <https://www.alice.cnptia.embrapa.br/alice/bitstream/doc/923530/1/balbino01.pdf> (2011)

9. Observatório ABC. *Agricultura de Baixa Emissão de Carbono: A evolução de um novo paradigma* <https://s3-sa-east-1.amazonaws.com/arquivos.gvces.com.br/arquivos_gvces/arquivos/275/abc_novoparadgima_completo.pdf> (2013).

10. Bustamante, M. M. C., Corbeels, M., Scopel, E. & Roscoe, R. *Soil carbon and sequestration potential in the Cerrado Region of Brazil*. In: Lal, R.; Cerri, C.C.; Bernoux, M.; Etchevers, J. & Cerri, C.E.P. Carbon sequestration in soils of Latin America. (New York, Haworth, 2006).

11. Carvalho, J. L. N. et al. Impact of pasture, agriculture and crop-livestock systems on soil C stocks in Brazil. *Soil Tillage Res.* **110,** 175-186 (2010).

12. Lima, A. M. N. et al. Soil organic carbondynamics following afforestation of degraded pastures with eucalyptus in southeastern Brazil. *Forest Ecol. Manag.* **235,** 219-231 (2006).

13. Costa Junior, C. et al. Assessing soil carbon storage rates under no-tillage: Comparing the synchronic and diachronic approaches. *Soil Tillage Res.* **134,** 207-212 (2013).

14. Cerri, C. E. P. et al. *Tropical agriculture and global warming: Impacts and mitigation options*. Sci. Agric. **64**, 83-99 (2007).

15. Ministério de Minas e Energia (MME). *Balanço de Energia Útil* (Brasília, 2005).

16. Ministério de Minas e Energia (MME). *Referência do Plano Duodecenal de Geologia, Mineração e Transformação Mineral 2030* (Brasília, 2009).

17. Ministério de Minas e Energia (MME) *Anuário Estatístico do Setor de Transformação de Não Metálicos 2015* (Brasília, 2015).

18. Agência Nacional do Petróleo, Gás Natural e Biocombustíveis. *Anuário Estatístico 2015.* (ANP, 2015)

19.Balanço Energético Nacional. *Séries Completas* <https://ben.epe.gov.br/BENSeriesCompletas.aspx>. (2016)

20. Associação Brasileira das Empresas Distribuidoras de Gás Canalizado. *Estatísticas* http://www.abegas.org.br/Site/?cat=27 (2016)

21. Ministério da Ciência e Tecnologia e Inovação (MCTI).  *Emissões Fugitivas na Mineração e Beneficiamento do Carvão Mineral. Relatórios de Referência: Energia. Terceiro Inventário Brasileiro de Emissões e Remoções Antrópicas de Gases de Efeito Estufa* http://sirene.mcti.gov.br/publicacoes (2015).

22. Associação Brasileira do Carvão Mineral. *Estatísticas*. (ABCM, 2016).

23. Associação Brasileira da Indústria Química. *Anuário da Indústria Química Brasileira 1973*. (ABIQUIM, 1973).

24. Associação Brasileira da Indústria Química. *Anuário da Indústria Química Brasileira 1974*. (ABIQUIM, 1974).

25. Associação Brasileira da Indústria Química. *Anuário da Indústria Química Brasileira 1985*. (ABIQUIM, 1985).

26. Associação Brasileira da Indústria Química. *Anuário da Indústria Química Brasileira 1990*. (ABIQUIM, 1990).

27. Ministério da Ciência e Tecnologia e Inovação (MCTI).  *Emissões de Gases de Efeito Estufa nos Processos Industriais: Indústria Química. Relatórios de Referência: Processos Industriais e Uso de Produtos. Terceiro Inventário Brasileiro de Emissões e Remoções Antrópicas de Gases de Efeito Estufa* http://sirene.mcti.gov.br/publicacoes (2015).

28. Associação Brasileira da Indústria Química. *Anuário da Indústria Química Brasileira 2015*. (ABIQUIM, 2015).

29. Ministério da Ciência e Tecnologia e Inovação (MCTI). *Emissões de Gases de Efeito Estufa no Tratamento e Disposição de Resíduos. Relatórios de Referência: Setor de Tratamento de Resíduos. Terceiro Inventário Brasileiro de Emissões e Remoções Antrópicas de Gases de Efeito Estufa* http://sirene.mcti.gov.br/publicacoes (2015).

30. Fundação de Ciência, Aplicações e Tecnologia Espaciais. *Emissões de dióxido de carbono no setor uso da terra, mudança do uso da terra e florestas* http://cetesb.sp.gov.br/inventario-gee-sp/emissoes-do-setor-de-uso-da-terra-mudanca-do-uso-da-terra-e-florestas/ (2010).

31. Instituto Nacional de Pesquisas Espaciais. *Projeto PRODES – Monitoramento da Floresta Amazônica por Satélite* http://www.obt.inpe.br/prodes (2012).

32. Instituto Brasileiro do Meio Ambiente. *Projeto de Monitoramento do Desmatamento dos Biomas Brasileiros por Satélite – PMDBBS* http://www.siscom.ibama.gov.br/monitoramentobiomas (2013).

33.Fundação SOS Mata Atlântica. *Publicações* https://www.sosma.org.br/quem-somos/publicacoes/ (2016)
